# Imbalance of Peripheral Lymphocyte Subsets in Patients With Ankylosing Spondylitis: A Meta-Analysis

**DOI:** 10.3389/fimmu.2021.696973

**Published:** 2021-07-06

**Authors:** Dong Liu, Budian Liu, Churong Lin, Jieruo Gu

**Affiliations:** ^1^ Department of Rheumatology, The Third Affiliated Hospital of Sun Yat-Sen University, Guangzhou, China; ^2^ Radiology Department, The Third Affiliated Hospital of Sun Yat-Sen University, Guangzhou, China

**Keywords:** Ankylosing spondylitis, lymphocyte, immune system, flow cytometry, Th17 & Treg cells

## Abstract

Ankylosing spondylitis is a complicated consequence of genetic predisposition and environmental factors. Enthesitis is believed to be the hallmark of ankylosing spondylitis, and the chronic inflammatory state of this disease is perpetuated by the disturbances of both the innate immune system and the acquired immune system. To clarify the alteration of immune system in patients with AS, we conducted a meta-analysis concerning the proportions of major lymphocyte subsets in the peripheral blood of AS patients. We systematically searched PubMed and China National Knowledge Infrastructure (CNKI) for articles related to this subject. A total of 95 articles involving 4,020 AS patients and 3,065 healthy controls were included in the analysis. This meta-analysis is performed on R platform using R package “meta”, and Egger’s tests were used to determine the presence of publication bias. Results showed that the percentages of T cells, NK cells and NKT cells were not significantly different between AS patients and healthy controls, but B cells were significantly increased. Among the subsets of T cells, the proportions of CD4+ T cells, Th17 cells, Tfh cells as well as Th1/Th2 ratio were significantly increased, while Tregs were significantly decreased. Subgroup analysis showed that the proportions of Th17 among both PBMCs, T cells and CD4+ T cells were significantly elevated, while Tregs were only significantly lower in PBMCs. Subgroup analysis also demonstrated that Tregs defined by “CD4+CD25+FoxP3+”, “CD4+CD25+CD127low”or “CD4+CD25+CD127-”were significantly downregulated, indicating that the selection of markers could be critical. Further study is warranted in order to elucidate the complicated interactions between different lymphocyte subsets in AS patients. This study implied that the disequilibrium between Th17 and Tregs, as well as between Th1 and Th2 could contribute to the pathogenesis of ankylosing spondylitis, further cementing the understanding that ankylosing spondylitis is a consequence of disrupted balance of innate immune system and acquired immune system.

## Introduction

Ankylosing spondylitis belong to the group of diseases known as spondyloarthrpathies, which is a spectrum of diseases encompassing psoriatic arthritis, reactive arthritis and undifferentiated spondyloarthritis (1). Clinical manifestations of ankylosing spondylitis include articular manifestations and extra-articular manifestions. The articular manifestations mainly involve axial skeleton presenting as inflammatory back pain, with peripheral oligoarthritis present in some of the patients, while the extra-articular manifestations include uveitis, gut inflammation and dactylitis (2–4). To date, the pathogenesis of ankylosing spondylitis has not yet been fully elucidated. Previous studies have revealed that ankylosing spondylitis is a consequence of genetic background and environmental factors, with HLA-B27 stepping into the limelight of research upon the discovery that HLA-B27 can be present in as many as 90% of patients with AS (5).

How HLA-B27 causes the disease of ankylosing spondylitis remains unclear, though several hypotheses have been put forward attempting to connect the dots (6, 7). Yet, it is undisputed that the disturbances of the immune system eventually perpetuate this disease (8–10). Unlike autoimmune diseases like systemic lupus erythematosus and rheumatoid arthritis, it is not the autoreactive B cells secreting auto-antibodies that should be held accountable, since no antibody is widely acknowledged to be detected in patients with ankylosing spondylitis (11). Instead, such disturbances in the immune system in patients with AS are the result of complicated interactions between the innate immune system and the adaptive immune systems (10). The successful application of biologics, especially TNF-α inhibitors, provide substantial evidence that by blocking cytokines characteristic of the innate immune system, the inflammatory status can be greatly alleviated (12, 13). On the other hand, numerous studies have added to the confirmation of the fact that AS is driven by the imbalances of lymphocyte subsets, especially the Th17/Tregs and Th1/Th2 imbalances, disrupting the equilibrium of the immune system (14, 15). The specific CD4+ T cell subset of Tregs possess immunosuppressive features (16), and the incapability of Tregs may allow the over-secretion of pro-inflammatory cytokines, especially IL-17, which is a potent pro-inflammatory cytokine secreted by Th17 and plays an important role in mediating bone damage (17). Meanwhile, the hyperactivation of the Th1 effector T cell lineage may secrete abundant IFNγ and TNF-α (18), leading to the chronic inflammatory state of the disease.

However, different studies have provided conflicting data regarding the direction and extent of the imbalance of lymphocytes. Most studies suggested that the percentages of Tregs were significantly decreased in patients with AS, yet a few studies found that Tregs might be increased in the peripheral blood of AS patients, arguing that the increase of Tregs might be the result of an attempt to enhance immune tolerance to control the immune response. More intriguingly, the proportions of NK cells is the peripheral blood of AS patients were heavily debated. It has been hypothesized that KIR3DL2, an inhibitory receptor expressed on NK cells, might inhibit apoptosis of NK cells once ligated with HLA-B27, leading to an excess of NK cells in the peripheral blood. In the meanwhile, a few studies observed a significant decrease in the proportions of NK cells in AS patients. Based on previous studies, we hypothesized that the elevation of Th17 and the downregulation of Tregs were pivotal in the pathogenesis of AS, while the th1/th2 polarization might also be involved. In order to clarify the actual proportions of different subsets of lymphocytes, we conducted a meta-analysis concerning the lymphocyte imbalances in the peripheral blood in patients with AS, with healthy donors as the control.

## Methods

### Data Sources and Searches

We searched the relevant studies using PubMed, Cochrane, Medline and China National Knowledge Infrastructure (CNKI). The literature search strategy used the following terms: (“ankylosing spondylitis”) AND (“lymphocyte subsets” OR “T cell” OR “B cell” OR “Th1” OR “Th2” OR “Th17” OR “Treg” OR “NK cell” OR “NKT cell” OR “gamma delta T cell” OR “flow cytometry”). The publication date was set before April 1, 2021, and all potential eligible studies were screened except for animal experiments or reviews. Some of the studies listed in the reference were retrieved through reference literature in related articles.

### Study Selection

The inclusion criteria were as follows: (a) original research; (b) human research; (c) studies with full text available; (d)studies that provided data concerning proportions of certain lymphocyte subsets in peripheral blood of AS patients; (e) studies that provided information concerning flow cytometry experiment protocol and subject characteristics.

The following criteria is used to exclude studies from the final analysis: (a) Studies that did not provide data in the form of mean and standard deviation, or data that could not be transformed; (b) Studies focusing on certain tissue instead of peripheral blood; (c)Duplicates already included once in the analysis.

Two independent researchers (Dong Liu and Budian Liu) extracted data from eligible articles according to the inclusion criteria, while a third investigator settled any disagreements (Churong Lin). Extracted data included author’s name, publication year, baseline characteristics, number of patients and healthy controls, markers of lymphocytes, diagnostic criteria and proportions of each lymphocyte subset in PBMC or T cells or CD4+ T cells. Data were recorded as mean and standard deviation. If the percentages of lymphocytes were presented as median or interquartile range yet no obvious skewing is identified, the data is transformed to mean and standard deviation. (SD = IQR/1.35) The Newcastle-Ottawa Quality Assessment Scale was used to assess the quality of studies.

### Statistical Analysis

This meta-analysis was perfomed on the R platform, using R package “meta” [v4.13-0; (19)]. The Cochrane chi-squared test was used to assess the heterogeneity of the included studies. If the heterogeneity of the studies were high (), then the random-effects model was employed to conduct the analysis. Subgroup analysis was performed when it was deemed necessary to break down the analysis on levels of comparison (PBMC, T cells or CD4+ T cells) or based on different markers. Considering the heterogeneity of the literature since different classification criteria were applied, and disease activity of the patients varied across studies, we also conducted subgroup analysis based on classification criteria and disease activity. Publication bias was assessed by the Egger’s test (p≥0:05). Sensitivity analyses was conducted to test the robustness of the results.

## Results

### Study Characteristics

**Figure 1:**
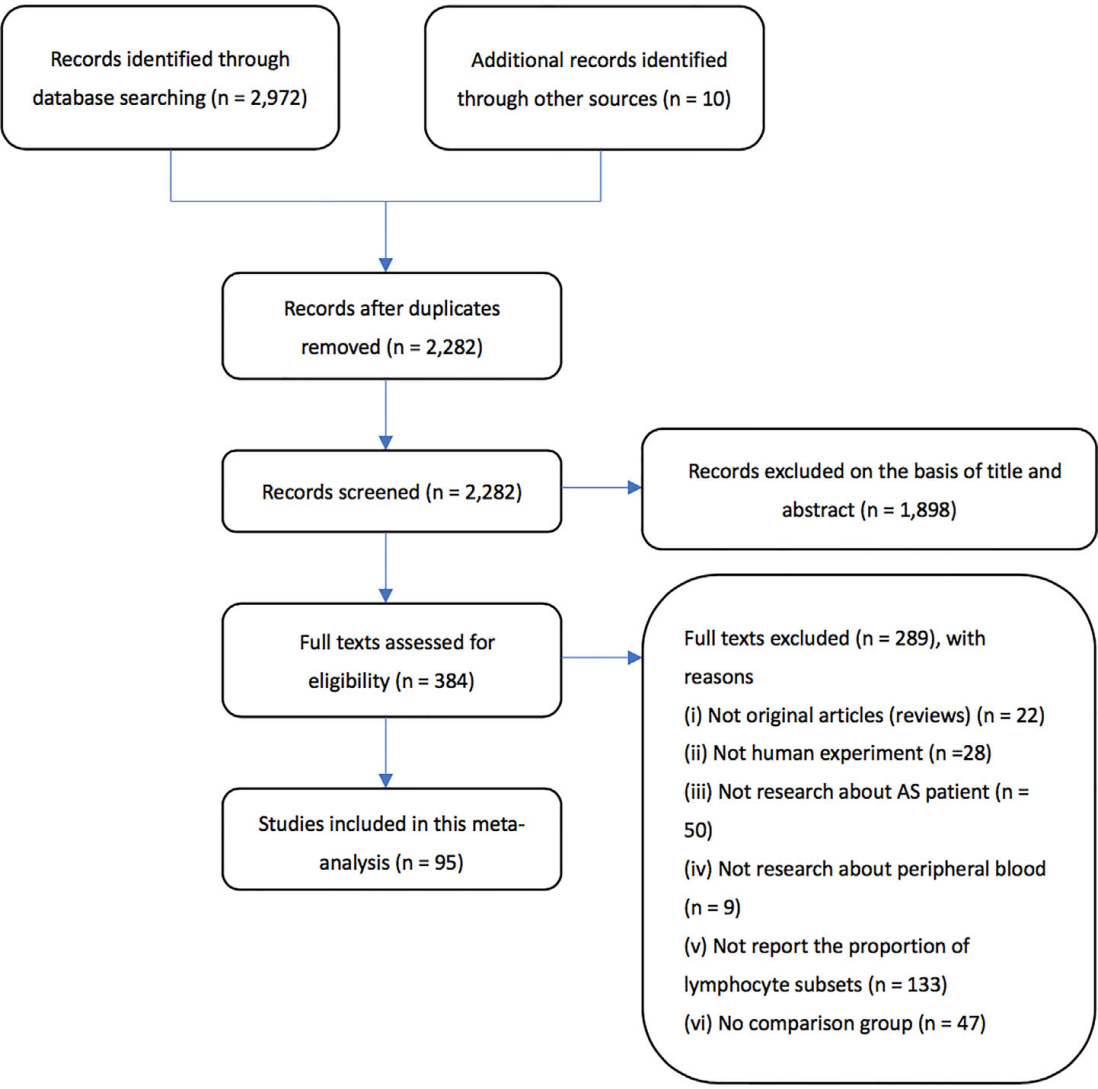
Flow chart of the literature search and study selection process.

Based on the methods stated above, a total of 2,982 articles were retrieved. We excluded 700 articles since they were duplicates. Next, through screening the abstracts of the articles, a total of 384 articles were included. After carefully examining the articles, articles without full text or failing to provide original data were excluded, leaving 95 articles eligible to be included in the final meta-analysis. Flow chart of the literature search process can be seen in **Figure 1**.

This meta-analysis included 4020 AS patients and 3065 healthy controls from 95 eligible studies. The features of these studies can be seen in **Table 1**. Of all the studies, 19, 19, 13 and 3 studies provided data on the proportion of T cells, B cells, NK cells and NKT cells. As for the subsets of T cells, 37, 32 and 3 studies focused on CD4+ T cells, CD8+ T cells and γδ T cells. Delving into the CD4+ T cells, 19, 12, 28, 46 and 3 studies presented data on the proportions of Th1, Th2, Th17, Tregs and Tfh cells. Six and 7 studies further discussed the Th1/Th2 proportions and Th17/Treg proportions. All studies had a NOS score of 3-7; the qualities of these studies were moderate. The original data can be seen in **Supplemental Material 1**.

**Table 1:** Characteristics of 95 studies included in this meta-analysis.

Author (Ref.)	Publish year	Country	Case numbers (AS/HC)	Lymphocyte subsets discussed	Age (year) AS/HC	Disease activity	Diagnosis criteria	NOS score	Database
An et al. (20)	2019	China	73/85	Th17/Treg ratio	nr	nr	mNY1984	5	Medline; PubMed
Appel et al. (21)	2011	Germany	19/20	Treg	40.9 ± 13.8/nr	nr	mNY1984	4	Medline; PubMed
Bautista et al. (22)	2014	Norway	25/50	Tfh	56 ± 14.8/nr	nr	mNY1984	5	Medline; PubMed
Bidad et al. (23)	2013	Iran	18/18	Th17; Treg	34 ± 2/33 ± 1	BASDAI≥4	mNY1984	6	Medline; PubMed
Brand et al. (24)	1997	Germany	21/29	B; CD4+T; CD8+T	42 ± 14/47 ± 16	nr	mNY1984	5	Medline; PubMed
Cai and Xiao (25)	2013	China	40/20	Treg	29 ± 9.4/28.4 ± 10.3	nr	mNY1984	7	CNKI
Cai et al. (26)	2005	China	30/20	B; T; CD4+T; CD8+T	nr	nr	mNY1984	4	CNKI
Cao et al. (27)	2004	Sweden	10/29	Treg	nr	nr	mNY1984	4	PubMed
Chen et al. (28)	2013	China	61/36	Th1; Th17; Treg	25 ± 8.2/25 ± 7	nr	mNY1984	7	CNKI
Chen et al. (29)	2011	Taiwan (China)	23/25	B; T; CD4+T; Treg	nr	nr	mNY1984	6	Medline; PubMed
Cheng (30)	2007	China	25/21	CD4+T	28 ± 9/27 ± 6	nr	mNY1984	6	CNKI
Dejaco et al. (31)	2010	Austria	22/17	Treg	40.9 ± 12.7/40.3 ± 23.4	nr	nr	3	Medline; PubMed
Deng et al. (32)	2019	China	49/100	Th1; Th2; Th1/Th2 ratio	28.31 ± 6.72/27.38 ± 6.39	nr	mNY1984	7	CNKI
Deng et al. (33)	2018	China	91/50	T; CD4+T; CD8+T	nr	nr	mNY1984	6	CNKI
Dong et al. (34)	2006	China	30/30	T; CD4+T; CD8+T	nr	nr	mNY1984	5	CNKI
Duan et al. (35)	2017	China	21/16	T; CD4+T; CD8+T; Treg	37 ± 9.8/34.6 ± 10.1	BASDAI≥4	mNY1984	6	Medline; PubMed
Dulic et al. (14)	2017	Hungary	7/10	CD4+T; CD8+T; Th1; Th2; Th1/Th2 ratio; Th17; Treg; Th17/Treg ratio	nr	nr	mNY1984	6	Medline; PubMed
Fattahi et al. (36)	2018	Iran	30/15	Th17; Treg	31.4 ± 9.1/32.1 ± 8.2	BASDAI≥4	mNY1984	6	Medline; PubMed
Forger et al. (37)	2009	Switzerland	15/18	Treg	nr	nr	mNY1984	5	PubMed
Gao et al. (38)	2012	China	40/37	Th17; Treg	29.1 ± 8.6/26.7 ± 6.9	nr	mNY1984	6	CNKI
Guo et al. (39)	2012	China	98/76	CD4+T; CD8+T	nr	nr	nr	5	CNKI
Hajialilo et al. (40)	2019	Iran	24/35	Th17	nr	BASDAI≥4	ASAS2009	6	Cochrane; Medline; PubMed
Han et al. (41)	2006	China	69/50	B; T; CD4+T; CD8+T	nr	nr	mNY1984	5	CNKI
He et al. (42)	2012	China	32/50	B; CD4+T; NK	nr	nr	nr	4	CNKI
Hu et al. (43)	2019	China	60/40	B; CD4+T; CD8+T; NK	nr	nr	mNY1984	5	CNKI
Hu et al. (44)	2013	China	32/30	T; CD4+T; CD8+T; Th1; Th2	34 ± 3.89/36 ± 3.76	nr	mNY1984	4	CNKI
Huang et al. (45)	2009	China	20/9	CD4+T; Treg	nr	nr	mNY1984	4	CNKI
Huang et al. (46)	1990	China	9/9	CD4+T; CD8+T	nr	nr	mNY1984	4	CNKI
Ji et al. (47)	2014	China	20/20	Treg	nr	nr	mNY1984	7	Medline; PubMed
Kenna et al. (48)	2012	Australia	17/20	γδT; Th17	39.47 ± 13.6/nr	nr	mNY1984	6	Medline; PubMed
Kim et al. (49)	2019	South Korea	49/53	CD4+T; CD8+T; NK	36.4 ± 10.8/34.9 ± 9	nr	mNY1984	6	Medline; PubMed
Klasen et al. (50)	2019	Germany	14/5	Th17	42.7 ± 3.15/nr	nr	mNY1984	5	Medline; PubMed
Li (51)	2019	China	64/60	Th17; Treg	33.26 ± 5.74/35.84 ± 6.19	nr	mNY1984	7	CNKI
Li et al. (52)	2013	China	222/68	Th17; Treg	33.6 ± 8/34.1 ± 10.6	BASDAI≥4	mNY1984	6	Medline; PubMed
Li et al. (53)	2009	China	30/10	Th1; Th2; Th1/Th2 ratio	nr	BASDAI≥4	mNY1984	5	CNKI
Li et al. (54)	2008	China	50/21	T; CD8+T	25 ± 8/25 ± 5	nr	mNY1984	6	CNKI
Liao et al. (55)	2015	Taiwan (China)	69/30	Treg	39.6 ± 12.7/44.3 ± 10.5	nr	mNY1984	7	Medline; PubMed
Limon-Camacho et al. (56)	2012	Mexico	39/25	Th1; Th2; Th17; Treg	32 ± 13/32 ± 8	BASDAI≥4	mNY1984	4	PubMed
Lin et al. (57)	2008	China	66/30	CD4+T; CD8+T	29.7 ± 9.6/26.7 ± 6.7	nr	mNY1984	6	CNKI
Lin et al. (58)	2009	China	66/30	B	29.7 ± 9.6/26.7 ± 6.7	nr	mNY1984	6	Cochrane; Medline; PubMed
Liu and Feng et al. (59)	2017	China	38/38	Th1; Th17	39.3 ± 3.4/40.4 ± 3.9	nr	mNY1984	6	CNKI
Liu et al. (60)	2016	China	60/20	Treg	35 ± 10.7/41.9 ± 11.7	nr	mNY1984	5	CNKI
Liu et al. (61)	2012	China	60/30	Treg	31.5 ± 9.1/nr	nr	mNY1984	6	CNKI
Liu et al. (62)	2010	China	30/20	Th1/Th2	26 ± 3.69/25.15 ± 3.79	nr	mNY1984	6	CNKI
Long et al. (63)	2018	China	65/20	CD4+T; Tfh	27.8 ± 8.5/31.4 ± 7.4	nr	mNY1984	6	Medline; PubMed
Ma et al. (64)	2011	China	36/32	B; CD4+T; CD8+T; NK	23.1 ± 4.8/25.8 ± 3.6	nr	mNY1984	5	CNKI
Ma et al. (65)	2011	China	43/20	B; T; CD4+T; CD8+T; NK	nr	nr	mNY1984	4	CNKI
Ma et al. (66)	2004	China	25/30	B; T; CD4+T; CD8+T; NK	nr	nr	mNY1984	5	CNKI
Meng et al. (67)	2015	China	42/20	CD8+T;	32.4 ± 9.3/29.5 ± 8.4	nr	mNY1984	6	CNKI
Mo et al. (68)	2019	China	30/23	B; CD4+T; CD8+T; γδT; NK	40.7 ± 3.18/45.71 ± 2.6	nr	mNY1984	7	CNKI
Pishgahi et al. (69)	2020	Iran	31/35	Th17; Treg	nr/41.89 ± 11.29	nr	ASAS2009	5	Medline; PubMed
Shan et al. (70)	2015	China	20/10	Treg	nr	BASDAI≥4	mNY1984	6	Medline; PubMed
Shen et al. (71)	2009	China	10/16	Th17	46.05 ± 11.51/nr	nr	mNY1984	3	Medline; PubMed
Suen et al. (72)	2008	Taiwan (China)	23/26	Treg	43 ± 12/37 ± 12	nr	nr	4	Medline; PubMed
Szalay et al. (15)	2012	Hungary	13/9	CD4+T; CD8+T; Th1; Th2; Th1/Th2 ratio; Th17/Treg ratio	43.7 ± 9.2/nr	BASDAI≥4	mNY1984	4	Medline; PubMed
Szanto et al. (73)	2008	Hungary	42/52	B; T; CD4+T; CD8+T; Th1; Th2; NK	nr	BASDAI≥4	mNY1984	5	Medline; PubMed
Thoen et al. (74)	1987	Norway	31/15	CD4+T; CD8+T	32 ± 1.8/nr	nr	mNY1984	3	Medline; PubMed
Toussirot et al. (75)	2009	France	32/15	Treg	42.9 ± 1.1/44.4 ± 0.8	nr	nr	4	PubMed
Wang et al. (76)	2020	China	90/90	Th17; Treg; Th17/Treg ratio	43.27 ± 8.19/43.55 ± 8.6	nr	nr	5	CNKI
Wang et al. (77)	2018	China	30/30	Th1; Th17; Treg	31.2 ± 4.1/nr	nr	mNY1984	6	CNKI
Wang et al. (78)	2018	China	26/26	Treg	33.5 ± 8.4/31.5 ± 10.2	ASDAS ≥ 2.1	mNY1984	4	Medline; PubMed
Wang et al. (79)	2016	China	50/50	CD4+T; γδT	28.53 ± 8.15/27.93 ± 8.52	nr	mNY1984	7	Medline; PubMed
Wang et al. (80)	2015	China	78/30	Treg; Th17/Treg ratio	26 ± 7.8/25 ± 8	nr	mNY1984	6	CNKI
Wang et al. (81)	2015	China	45/20	T; CD8+T; Th17; Treg	nr/55.05 ± 6.42	nr	nr	5	Medline; PubMed
Wang et al. (82)	2012	China	60/44	B; T; CD4+T; CD8+T; NK	nr	nr	mNY1984	6	CNKI
Wang et al. (83)	2008	China	30/20	CD4+T; CD8+T; Th1; Th2	26 ± 3.69/25.15 ± 3.79	nr	mNY1984	6	CNKI
Wei et al. (84)	2017	China	131/127	B; T; CD4+T; CD8+T; Treg; NK	27 ± 8/26 ± 9	nr	mNY1984	6	CNKI
Wu (85)	2014	China	60/60	Tfh	26.9 ± 7.8/24.3 ± 5.6	nr	mNY1984	6	CNKI
Wu et al. (86)	2011	China	51/49	Treg	nr	BASDAI≥4	mNY1984	6	Medline; PubMed
Wu et al. (87)	2011	China	24/30	B	35 ± 14/33 ± 12	nr	mNY1984	6	CNKI
Xu et al. (88)	2019	China	18/9	Th17; Treg	39.4 ± 2.3/42.6 ± 4.3	nr	mNY1984	6	Medline; PubMed
Xu et al. (89)	2018	China	69/22	CD4+T; CD8+T; NKT	nr	nr	mNY1984	6	CNKI
Xu (90)	2013	China	24/22	Th1; Th17; Treg	24.3 ± 8.5/27.9 ± 8.6	nr	mNY1984	6	CNKI
Xu et al. (91)	2011	China	78/50	B; T; CD4+T; CD8+T; Th1; Th2	nr	nr	nr	5	CNKI
Xue et al. (92)	2015	China	38/30	Th17; Treg	29.93 ± 9.82/30.58 ± 8.39	nr	mNY1984	6	CNKI
Xue et al. (93)	2008	China	89/42	T; CD4+T; CD8+T	nr	nr	nr	5	CNKI
Yang et al. (94)	2020	China	67/50	B; Th1; Th2; Th17	nr	ASDAS ≥ 1.3	mNY1984	6	Medline; PubMed
Yang et al. (95)	2018	China	30/30	T; NKT	29.3 ± 5.9/31.1 ± 6.7	nr	mNY1984	6	CNKI
Yang et al. (96)	2017	China	40/40	Treg	32.53 ± 9.76/33.7 ± 10.06	nr	mNY1984	6	CNKI
Yang et al. (97)	2016	China	38/31	Treg	28.9 ± 10.8/29.1 ± 8.1	nr	mNY1984	7	CNKI
Yang et al. (98)	2007	China	60/30	B; T; CD4+T; CD8+T	nr	nr	mNY1984	6	CNKI
Ye et al. (99)	2013	China	21/27	Treg	36.6 ± 10.2/37.9 ± 9.1	nr	mNY1984	3	Medline; PubMed
Zhang et al. (100)	2019	China	60/30	Th17; Treg; Th17/Treg ratio	43 ± 11/32 ± 12	nr	mNY1984	5	CNKI
Zhang et al. (101)	2019	China	39/41	B; T; CD4+T; CD8+T; NK	28.87 ± 8.31/27.05 ± 6.63	nr	mNY1984	6	CNKI
Zhang et al. (102)	2014	China	60/60	Th1; Th17; Treg	39 ± 3.2/39.2 ± 3.1	nr	mNY1984	6	CNKI
Zhang et al. (103)	2014	China	10/10	Th17	nr	nr	mNY1984	4	CNKI
Zhang et al. (104)	2012	China	32/20	Th1; Th17	36.6 ± 10.2/37.9 ± 9.1	nr	mNY1984	6	Medline; PubMed
Zhang et al. (105)	2008	China	78/50	Treg; NK	26.1 ± 6.8/25.5 ± 3.8	BASDAI≥4	mNY1984	7	CNKI
Zhao and Li (106)	2013	China	21/20	Th17; Treg; Th17/Treg ratio	nr/26 ± 8	BASDAI≥5	mNY1984	5	CNKI
Zhao et al. (107)	2011	China	14/18	Treg	26.4 ± 6.1/28.2 ± 9.4	nr	mNY1984	5	Medline; PubMed
Zhao et al. (108)	2009	China	30/30	CD4+T; CD8+T	nr	nr	mNY1984	5	CNKI
Zhong and Ma (109)	2014	China	78/30	Th1; Th2; Th17	nr	nr	mNY1984	6	CNKI
Zhu et al. (110)	2017	China	42/42	CD4+T	nr	nr	nr	3	CNKI
Zhu et al. (111)	2016	China	30/30	NK	nr	nr	mNY1984	5	CNKI
Zhu et al. (112)	2000	China	14/7	Th1; Th2; Th1/Th2 ratio	nr	nr	mNY1984	4	CNKI

AS, ankylosing spondylitis; HC, healthy control; nr, not reported; BASDAI, Bath Ankylosing Spondylitis Activity Disease Activity Index; ASDAS: Ankylosing Spondylitis Disease Activity Score; mNY1984: 1984 Modified New York AS Criteria; ASAS2009: 2009 Assessment of SpondyloArthritis international Society (ASAS) Criteria.

### Proportions of T Cells

**Figure 2:**
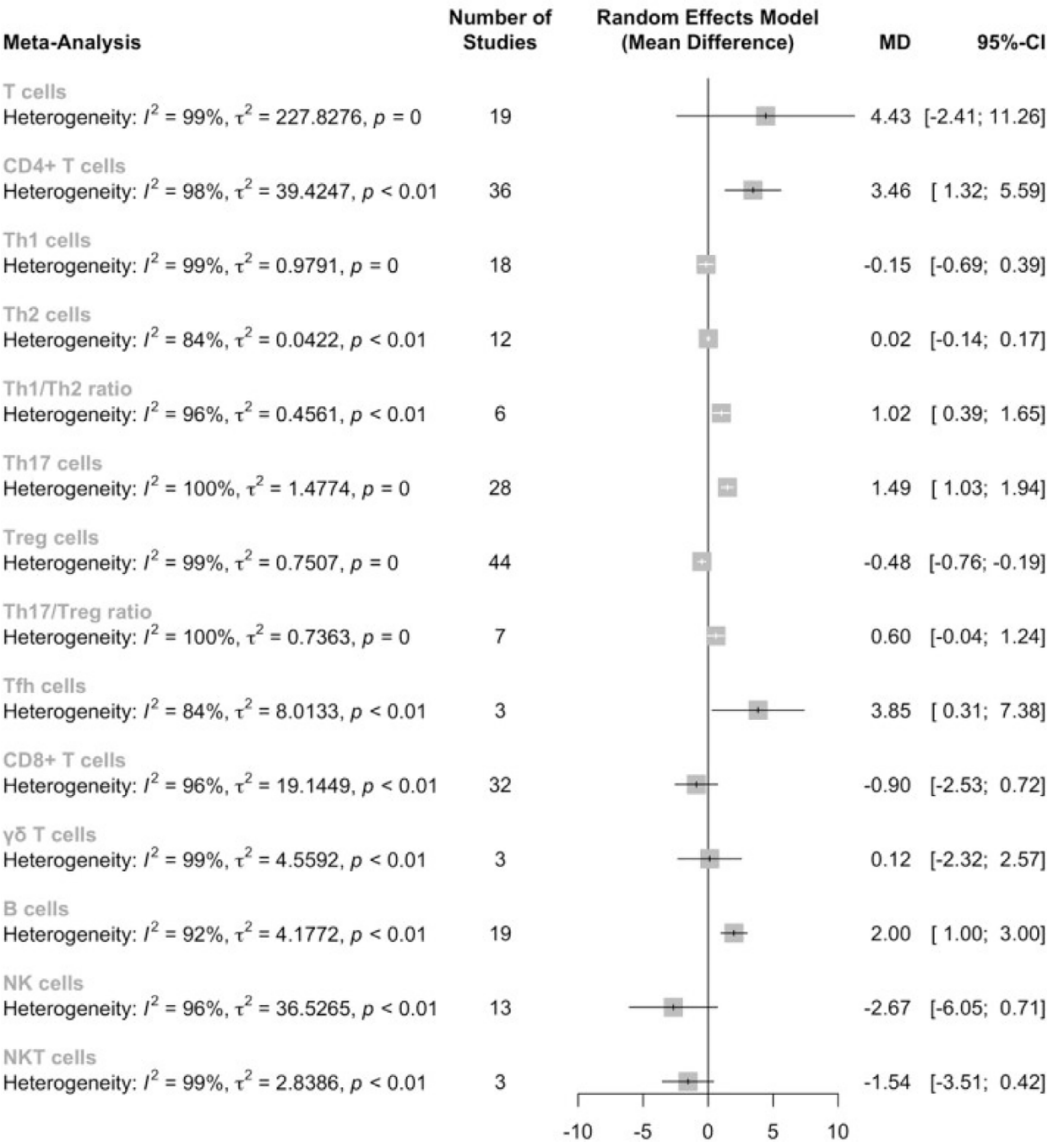
Proportions of major lymphocyte subsets in the peripheral blood of AS patients.

Firstly, we conducted a meta-analysis on the proportions of T cells in PBMC between AS patients and healthy controls, as well as the subsets of T cells in the corresponding category (**Figure 2**). Results showed that there is no significant difference in the T cell proportion between AS patients and healthy controls [4.43, (-2.41,11.26), p<0.01]; however, the proportion of CD4+ T cells was significantly elevated [3.32. (1.21,5.43), p<0.01]. When examining the subsets of the CD4+ T cells, we identified significant increases in the proportion of Th17 cells[1.49, (1.03,1.65), p<0.01], Tfh cells[3.85, (0.31,7.38), p<0.01] and Th1/Th2 ratio[1.02, (0.39,1.65), p<0.01], while the proportion of Tregs was significantly decreased[-0.43, (-0.71,-0.15), p<0.01]. However, sensitivity analysis indicated that the significantly lower proportions of Tfh cells could be insignificant by omitting either Long et al, or Wu et al. No significant difference was found in the level of Th1, Th2 cells. Noteworthy, Th17/Treg ratio was increased but did not reach statistical significance [0.60, (-0.04,1.24), p<0.01]. According to the sensitivity analysis, if omitting Wang et al, the Th17/Treg ratio could be significantly elevated (See **Supplemental Material 2**).

**Figure 3:**
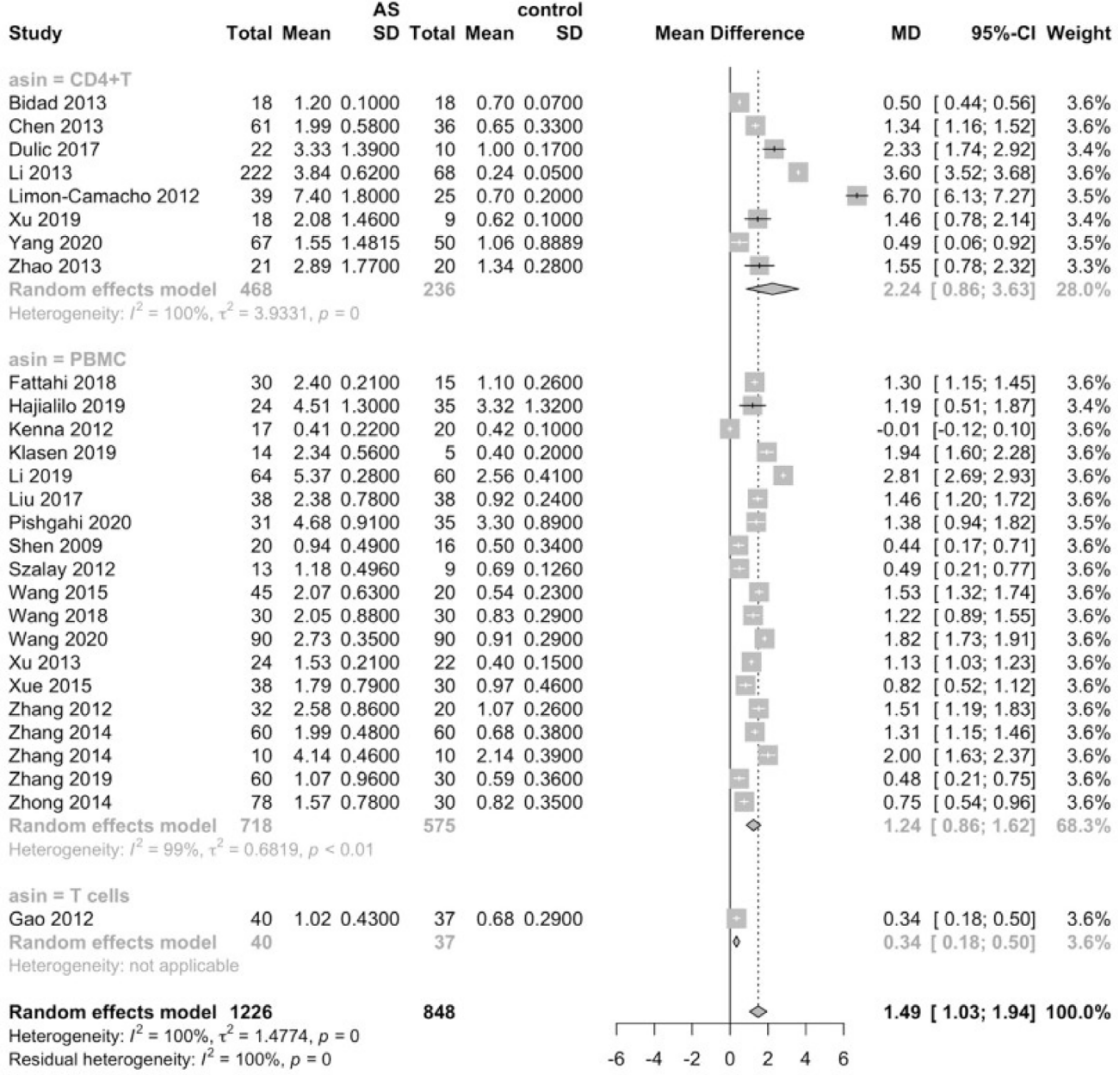
Proportions of Th17 cells among PBMCs, T cells and CD4+ cells.

**Figure 4:**
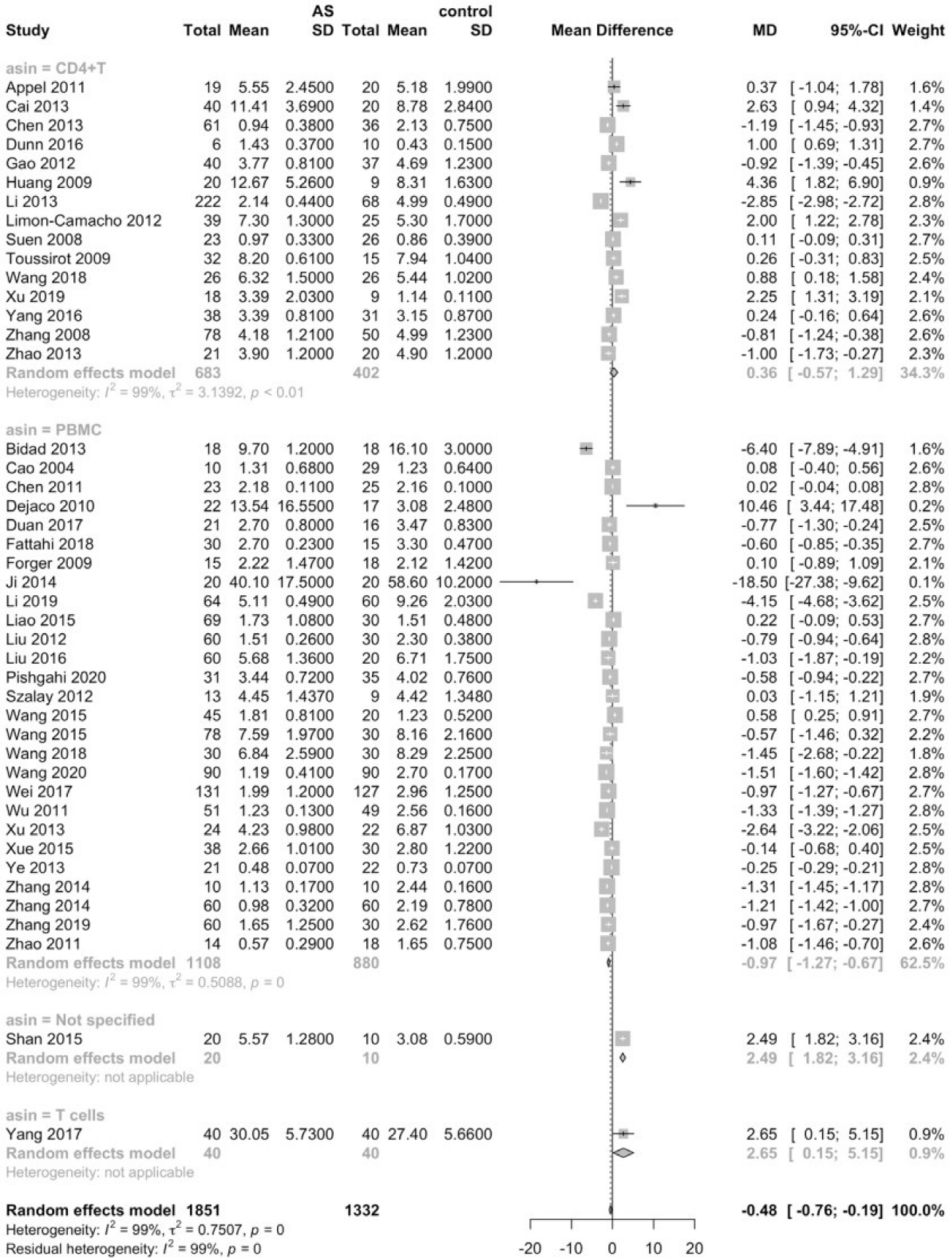
Proportions of Tregs among PBMCs, T cells and CD4+ cells.

**Figure 5:**
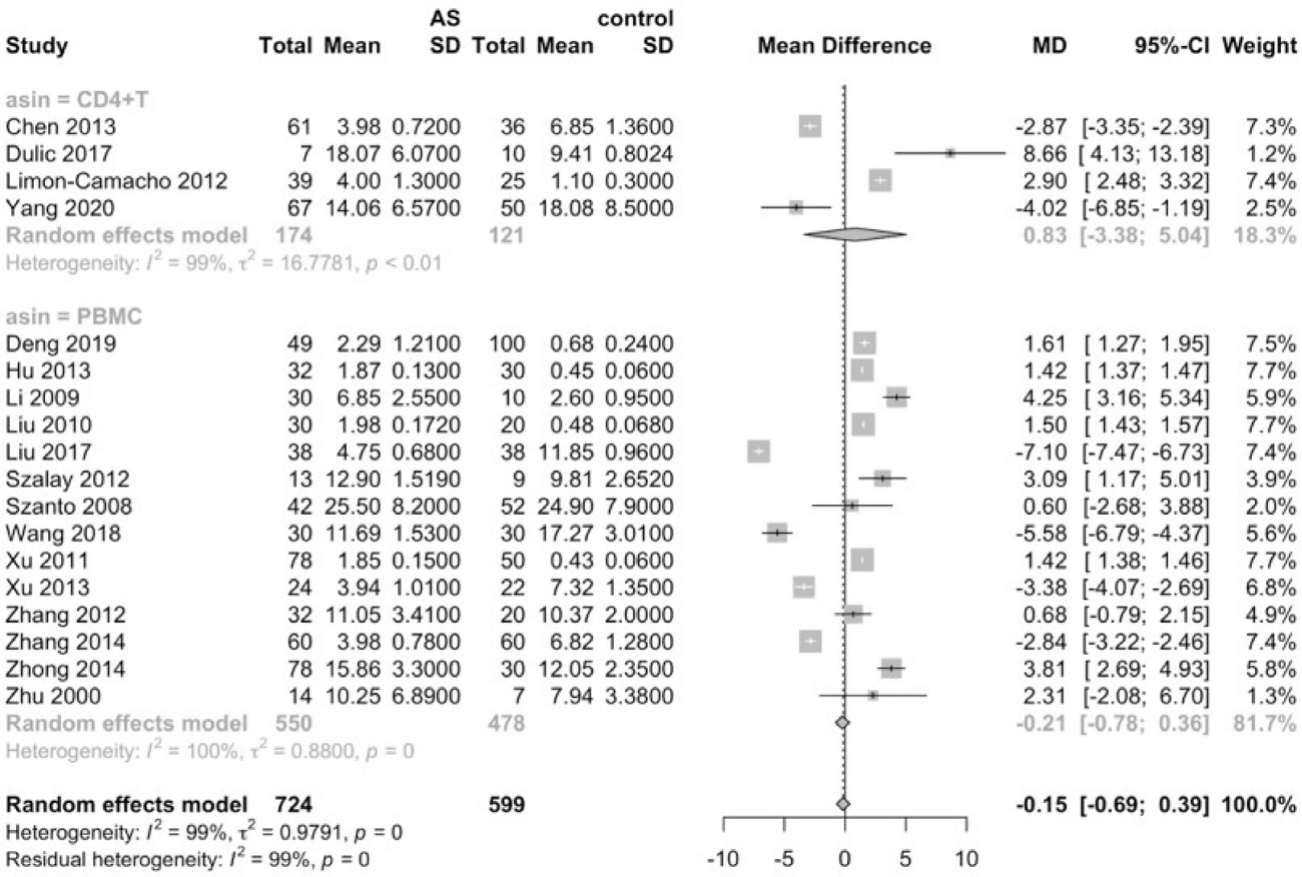
Proportions of Th1 cells among PBMCs and CD4+ cells.

**Figure 6:**
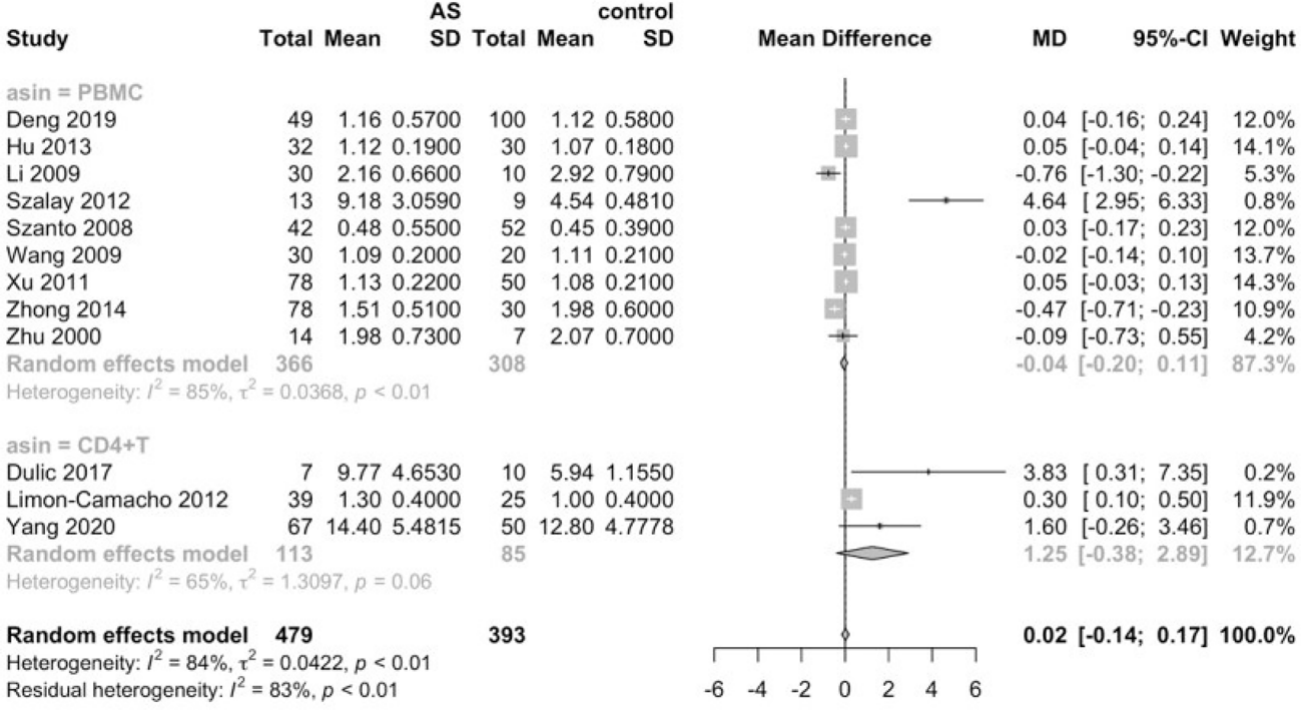
Proportions of Th2 cells among PBMCs and CD4+ cells.

Considering that the proportions of these lymphocytes were compared to PBMCs, T cells or CD4+ cells, we deemed it necessary to conduct subgroup analysis based on the level of comparisons. Subgroup analysis revealed that Th17 cells were increased on all the levels of PBMC, T cells and CD4+ cells (**Figure 3**), while Tregs were only significantly decreased on the level of PBMC (**Figure 4**). Still no significant difference was found in the proportions of Th1 and Th2 cells on all levels (**Figures 5**, **6**). On the other hand, due to the heterogeneity of the markers used to define Tregs, we also conducted a subgroup analysis of Tregs (**Table 2**). Results showed that Tregs defined by “CD4+CD25+FoxP3+”, “CD4+CD25+CD127low”or “CD4+CD25+CD127-”were significantly downregulated. No significant difference was detected in the proportions of CD8+ T cells and γδ T cells.

**Table 2:** Egger’s tests by different lymphocyte subsets.

Lymphocyte subset	t	df	p-value
T cells	0.49132	17	0.6295
CD4+ T cells	-1.9812	34	0.0557
Th1 cells	-1.6584	16	0.1167
Th2 cells	0.82077	10	0.4309
Th1/Th2 ratio	-0.55844	4	0.6063
Th17	0.23055	26	0.8195
Tregs	-0.086137	42	0.9318
Th17/Tregs	0.71323	5	0.5076
Tfh	0.16592	1	0.8953
CD8+ T cells	1.1247	30	0.2696
γδ T cells	-0.55802	1	0.676
B cells	1.7184	17	0.1039
NK cells	1.812	11	0.09735
NKT cells	-1.51	1	0.3724

Subgroup analysis further suggested that T cells were significantly elevated in patients with high disease activity, and that CD4+ T cells were still significantly increased in AS patients strictly defined by 1984 modified New York criteria. Furthermore, the proportion of Th17 cells remained elevated regardless of the classification criteria or disease activity, indicating the robustness of this result. Tregs were only significantly decreased in AS patients strictly defined by 1984 modified New York criteria. Intriguingly, though previous analysis failed to detect any alterations of Th1 proportions, subgroup analysis revealed that the Th1 lineage was elevated in AS patients with high disease activity, Still no alterations were observed in the proportions of Th2 cells and CD8+ T cells in AS patients (See **Supplemental Material 2**).

Egger’s tests showed that there was no obvious publication bias in all the subgroups of lymphocytes (**Table 3**).

**Table 3:** Subgroup analysis of Tregs proportions based on markers.

Treg definition	Number of studies (n)	SMD	95%CI	(%)	P
CD4+CD25+	3	1.11	(-1.77,3.98)	97	<0.01
CD4+CD25HI	6	0.23	(-0.26,0.71)	72	<0.01
CD4+FoxP3+	3	-1.32	(-6.12,3.49)	98	<0.01
CD4+CD25+FoxP3+	12	-0.75	(-1.28,-0.22)	99	<0.01
CD4+CD25+CD127LO	6	-2.18	(-3.55,-0.81)	97	<0.01
CD4+CD25+CD127-	4	-0.74	(-0.91,-0.57)	8	0.36
CD4+CD25HIFoxP3+	2	0.55	(-.33,1.42)	95	<0.01
CD4+CD25+CD127LO/-	4	-0.57	(-1.46,0.32)	84	<0.01
CD4+CD25+FoxP3+CD127-	1	0.88	(0.18,1.58)	/	/
Not specified	1	-1.51	(-1.6,-1.42)	/	/

### Proportion of B cells

According to the results of the meta-analysis, the proportion of B cells was significantly increased [2.00, (1.00,3.00), p<0.01]. Egger’s test found no publication bias in this result (**Table 3**). Sensitivity analysis indicated that this result was robust.

### Proportions of NK Cells and NKT Cells

No significant difference was found in the proportions of NK cells [-2.67, (-6.05,0.71)] and NKT cells[-1.54, (-3.51,0.42)]. Subgroup analysis based on classification criteria and disease activity still failed to detect any differences in the proportions of NK cells between AS patients and healthy controls (See **Supplemental Material 2**). Egger’s test found no publication bias in this result (**Table 3**).

Results of the sensitivity analyses can be found in the **Supplemental Material 2**.

## Discussion

This is the first meta-analysis to systemically examine the skewing of functional subgroups of lymphocytes, encompassing the major lymphocyte subsets, namely T cells, B cells, NK cells and NKT cells. Previous meta-analyses done by Li et al. and Lai et al. focused on regulatory T cells, arriving at the conclusion that the proportions of Tregs are significantly lower in both PBMCs and CD4+ T cells in patients with AS, though the markers used to define Tregs may have an impact on the proportions of Tregs (113, 114). In line with the results of previous studies, our study further confirmed that the levels of Tregs significantly decreased in patients with AS in PBMCs.

Tregs have been recognized as the essential subgroup of lymphocytes in charge of maintaining immune homeostasis and preventing autoimmunity. Immunosuppressive cytokines such as TGF-β and IL-10 secreted by Tregs may function as a negative regulator of immune responses and down-regulate excessive inflammatory status (115). For example, IL-10 secreted by Tregs may act directly on the IL-10 receptor on Th17 cells, thereby inhibiting the expansion of the inflammatory Th17 cells, or suppress the antigen-presenting cells and eventually suppress the responses of effector T cells (116). It has been reported that in patients with active AS, Tregs in peripheral blood fail to utilize IL-2 and cannot suppress naïve T cell proliferation (117). Moreover, application of TNF-α inhibitors can restore the proportion of Tregs, and the increase in Tregs is positively correlated with the decrease in CRP levels (94). Of note, different markers have been employed to identify the subgroup of Tregs, which may exert an influence on the proportions of Tregs measured in different studies, sometimes yielding contradictory results. Initially, Tregs is defined as CD4+CD25+, yet it was disputed since CD25 may also be expressed on cells without regulatory functions (118). Afterwards, the intracellular transcription factor (FOXP3) was proved to be exclusively expressed in Tregs and indispensable in the development of Tregs (119). The most common marker used to identify Tregs currently is CD4+CD25highCD127low or CD4+CD25highCD127-, of which CD127 is considered to be down-regulated on Tregs (113, 120). In our meta-analysis, we discovered that merely CD4+CD25+ did not produce significant outcomes regarding the proportions of Tregs, while Tregs defined by CD4+CD25+FoxP3+ and CD4+CD25+CD127low or CD4+CD25+CD127- were significantly lowered. This result of the subgroup analysis indicated that the CD127 could be a specific marker when trying to identify Tregs.

Upon the discovery of IL-23/IL-17 axis, the Th17 cells are moving center stage in the research of pathogenesis of spondyloarthropathies (121, 122). It has been widely acknowledged that enthesitis is the hallmark of spondyloarthropathies including AS, and recent research revealed that enthesitis is likely to be driven by the IL-23/IL-17 axis (123). IL-23, produced by myeloid cells either enthesis-resident or tissue infiltrating, may bind to the IL-23 receptors on Th17 cells as well as other lymphoid populations, and the activated Th17 cells can secrete IL-17, a powerful pro-inflammatory cytokine (123). Of the IL-17 family, IL-17A/IL-17F may act on stromal cells and other lymphocytes, which initiates the inflammatory process (17). It has also been reported that IL-17A may mediate bone damage by inducing the expression of RANK on the cell surface of osteoclasts, while also increasing the production of RANKL from mesenchymal stem cells (124). Apart from IL-17, Th17 lymphocytes are known to produce other pro-inflammatory mediators, such as IL-22, GM-CSF and TNF (125). All these studies further cemented the significance of Th17 cells in the pathogenesis of enthesitis, and, in a bigger picture, spondyloarthropathies. Our study substantiated that the levels of Th17 cells were significantly elevated, adding more concrete evidence to the critical role Th17 lineage plays in the pathogenesis in AS. Subgroup analysis further verified the robustness of this result, since Th17 cells were elevated on all levels of comparison, regardless of the classification criteria applied or disease activity of the patients.

In addition to Th17 cells, γδ T cells may also participate in the IL-23/IL-17 axis (123). γδ T cells are a specific population of T lymphocytes characterized by the highly diverse TCR on the cell surface, formulating TCR repertoire (126). Studies show that there is a 3-fold increase in the proportions of IL-23R-positive γδ T cells in AS patients, and such γδ T cells are also heavily skewed towards IL-17 production (48). Another study shows that IL-23R+ γδ T cells are the main producers of IL-17 in a mice model (127). More recent studies have revealed that IL-17 may also be produced in an IL-23-independent fashion (128). Therapies targeting IL-23 have failed in patients with SpA, while the downstream inhibition of IL-17 by IL-17A inhibitor Secukinumab and IL-17A/IL-17F inhibitor bimekizumab has yielded promising results in patients with SpA (129–131). Such phenomenon pointed to a possible pathway that IL-17 may be secreted without the stimulus of IL-23. It has been proved that γ/δ T cells may still secrete IL-17 despite the homozygous deletion of IL-23R (128). However, our study failed to recognize any alteration in the levels of γ/δ T cells. It could be attributed to the limited number of studies included, or that it was not the elevated number but the hyperactivity that was to blame for the IL-23 independent IL-17 secretion. Furthermore, RORγt+ iNKT cells were also reported to be able to secrete IL-17 with and without the effect of IL-23 (132).

In the meanwhile, the Th1/Th2 polarization of T helper cells is also a widely researched area in the immunity of AS (9). Th1 cells are known to mount immune responses against intracellular pathogens *via* secretion of IFNγ, which acts as a macrophage-activating factor (133). In addition to IFNγ, Th1 cells are also capable of producing IL-2, IL-10 and TNF-α, many of which participate in the inflammatory process (134, 135). Th2 cells, on the other hand, mainly assist in the humoral immune response (136). Cytokines secreted by Th2 cells include IL-4, IL-5 and IL-13, which facilitate the isotope switching of antibodies, mucus secretion and eosinophilia (137). Data concerning the Th1/Th2 skewing in the peripheral blood of AS patients has been highly inconsistent. Some studies reported that T helper cells in AS were skewed towards Th1 lineage suggesting that Th1 cells contributed to the excessive inflammation (56), while others failed to observe such elevations in proportions of Th1 cells (73). Our meta-analysis concluded that there was no significant alteration in the proportions of Th1 and Th2 cells overall, yet subgroup analysis revealed a significant increase in the percentages in AS patients with high disease activity, indicating that the Th1 lineage might be relevant in the acute phase. Meanwhile, the Th1/Th2 ratio was also significantly elevated. More recent research provides evidence that the plasticity of Th17 cells allows this subset of CD4+ T cells to partly assume phenotype of Th1 lineage or Th2 lineage, blurring the boundaries between Th1, Th2 and Th17 cells. It has been argued that the categorical dichotomy of Th1/Th2 should be rendered obsolete.

Another intriguing finding of our study is that the proportions of Tfh cells and B cells are significantly elevated in the peripheral blood of AS patients. Both Tfh cells and B cells participate in humoral immunity (136). After migrating to the B cell follicles, CD40L expressed on the cell surface of Tfh cells may interact with the CD40 on B cells serving as a stimulus signal, thereby facilitating the formation of germinal center, differentiation of B cells and ultimately the production of antibodies (63, 138). The relevance of humoral immunity in the pathogenesis of ankylosing spondylitis has long been underestimated, since no auto-antibody is universally acknowledged as the specific marker of AS (11). Although several studies have put forward that anti-CD74 antibody may serve as a potential biomarker for AS, its diagnostic utility awaits further confirmation (139). Another possible mechanism of the B lymphocyte involvement in the pathogenesis of AS is that B lymphocytes might mediate bone destruction through production of RANKL, as was previously reported in rheumatoid arthritis (140), How Tfh cells and B cells are involved in the pathogenesis of AS still requires more research.

As a pivotal component in the innate immunity, NK cells possess cytotoxic activity and the ability of producing pro-inflammatory cytokines, such as IFNγ (141). There is mounting evidence that NK cells, with its expression of KIR superfamily on the cell surface, may contribute to the pathogenesis of ankylosing spondylitis. Different KIRs may interact with HLA alleles in various forms, creating sophisticated genotypes of NK cells. It is hypothesized that the HLA-Bw4 group of alleles, notably HLA-B27, may bind to the KIR antigens with varying affinities, displaying inhibitory or stimulatory activities through downstream signal pathways (141–143). In particular, being an inhibitory receptor, KIR3DL2 ligation with HLA-B27 may inhibit apoptosis of NK cells and protect them from activation-induced cell death (142). However, studies regarding the frequency of NK cells in the peripheral blood of AS patients have been highly inconsistent. Azuz-Lieberman et al. found that AS patients have significantly higher percentages of NK cells in PB, while the inhibitory receptor CEACAM1 is highly expressed on the surface indicating suppressed function of NK cells (144). Another study also confirmed a higher frequency of NK cells expressing KIR3DL1 in SpA patients, with an impaired IFN-γ intracellular production in stimulated NK cells (145). However, such alteration of NK cell proportions was not observed in another study by Park, et al. (146) A more recent study found that NK cells in the peripheral blood was significantly reduced (84). Due to the inconsistencies of the data, our study failed to recognize a shift in the proportions of NK cells in the peripheral blood of AS patients.

There is no denying that there were some limitations to this study. Though being an all-encompassing meta-analysis attempting to include all the major subsets of lymphocytes, this study failed to conduct a more in-depth look into the more subtle minor subsets of lymphocytes, such as Th22 in CD4+ T cells, Tc1 and Tc2 in CD8+ T cells, Bregs, naïve B cells and memory B cells in the B cell lineage. This study originally intended to include Th22 subset in the meta-analysis, considering recent discovery that Th22 cells might have the capacity to promote osteoclast differentiation though production o IL-22 (147). To our chagrin, there were not enough studies to conduct an appropriate analysis. Second, lymphocytes may assume complicated phenotypes by expressing various antigens on the cell surface. Therefore, this crude classification of lymphocytes may not be adequate to explain the exact shifting of immune system in AS patients. However, further investigation was impeded by the insufficiency of the data. Third, there was notable heterogeneity in the studies considering the selected patients might have undergone different treatments and might be in different phases, it might be more appropriate to look into the effects of different treatments on the lymphocyte subsets. Moreover, this study only targeted lymphocytes in the peripheral blood, which could not adequately reflect the inflammatory status of the tissue.

In conclusion, our meta-analysis concluded that CD4+ T, Th17, Tfh and B cells were significantly elevated in the peripheral blood of AS patients, while Tregs were significantly reduced. Our study further cemented the understanding that the nature of ankylosing spondylitis is a hybrid of innate immunity and acquired immunity dysfunction.

## Data Availability Statement

The original contributions presented in the study are included in the article/**Supplementary Material**. Further inquiries can be directed to the corresponding author.

## Author Contributions

JG conceived of the presented idea. DL and BL conducted the literature search, while DL performed the analytic calculation and drafted the final version of this manuscript. CL settled any disagreements concerning the inclusion of literature. All authors contributed to the article and approved the submitted version.

## Conflict of Interest

The authors declare that the research was conducted in the absence of any commercial or financial relationships that could be construed as a potential conflict of interest.
