# Supplementary material for: Imbalance of Peripheral Lymphocyte Subsets in Patients With Ankylosing Spondylitis: A Meta-Analysis
Source: Front Immunol. 2021 Jul 6;12:696973. doi: 10.3389/fimmu.2021.696973 (PMC8291033; doi:10.3389/fimmu.2021.696973)
Supplement: Supplementary file 1 [file DataSheet_1.docx]

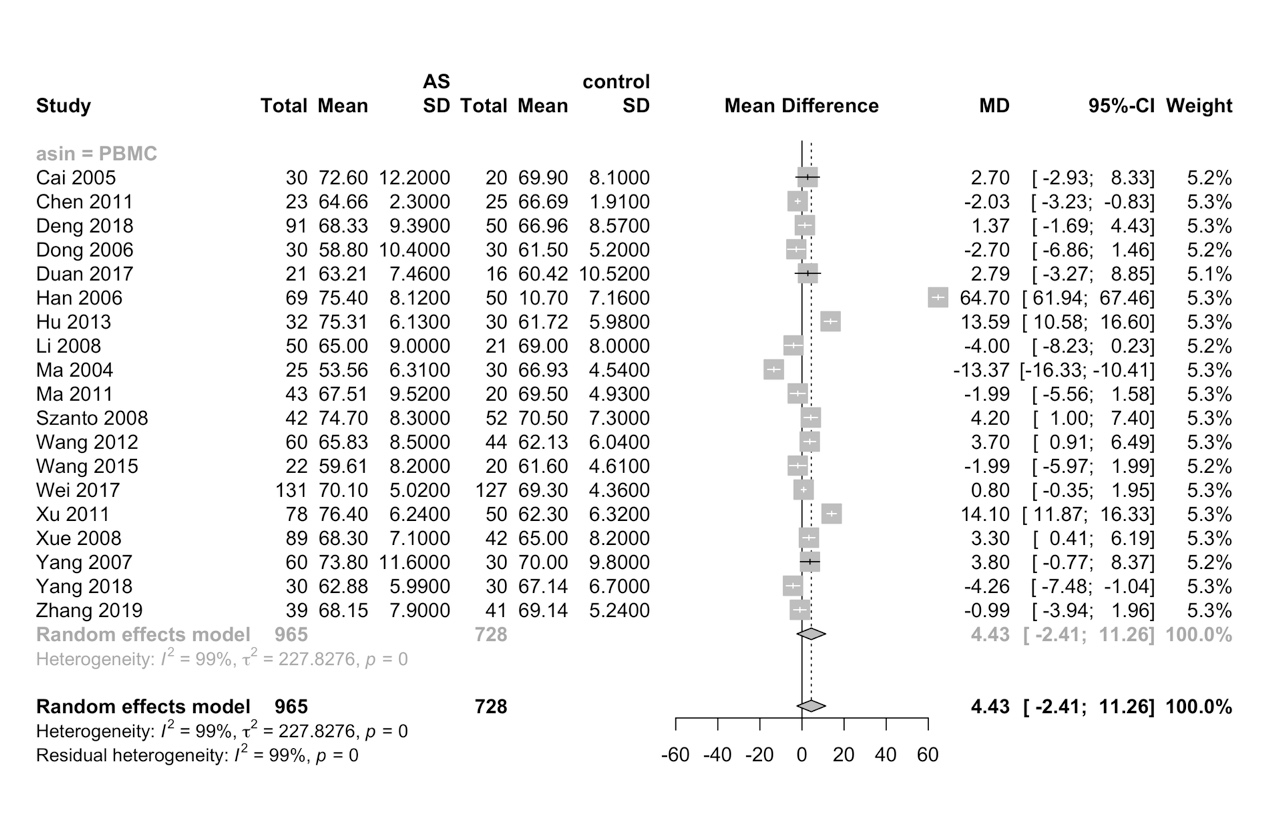


Figure 1. Proportions of CD3+ T cells in peripheral blood in AS patients


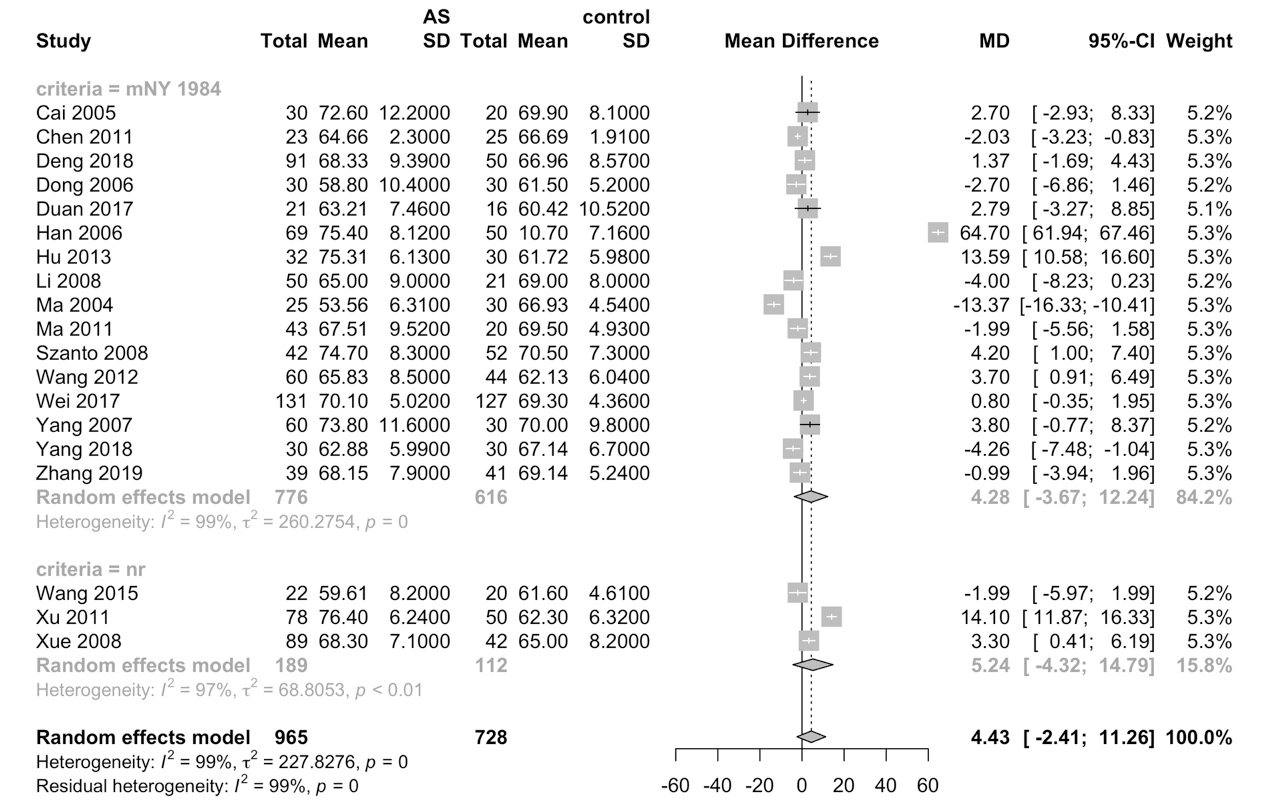


Figure 2. Subgroup analysis of proportions of CD3+ T cells in peripheral blood in AS patients by classification criteria


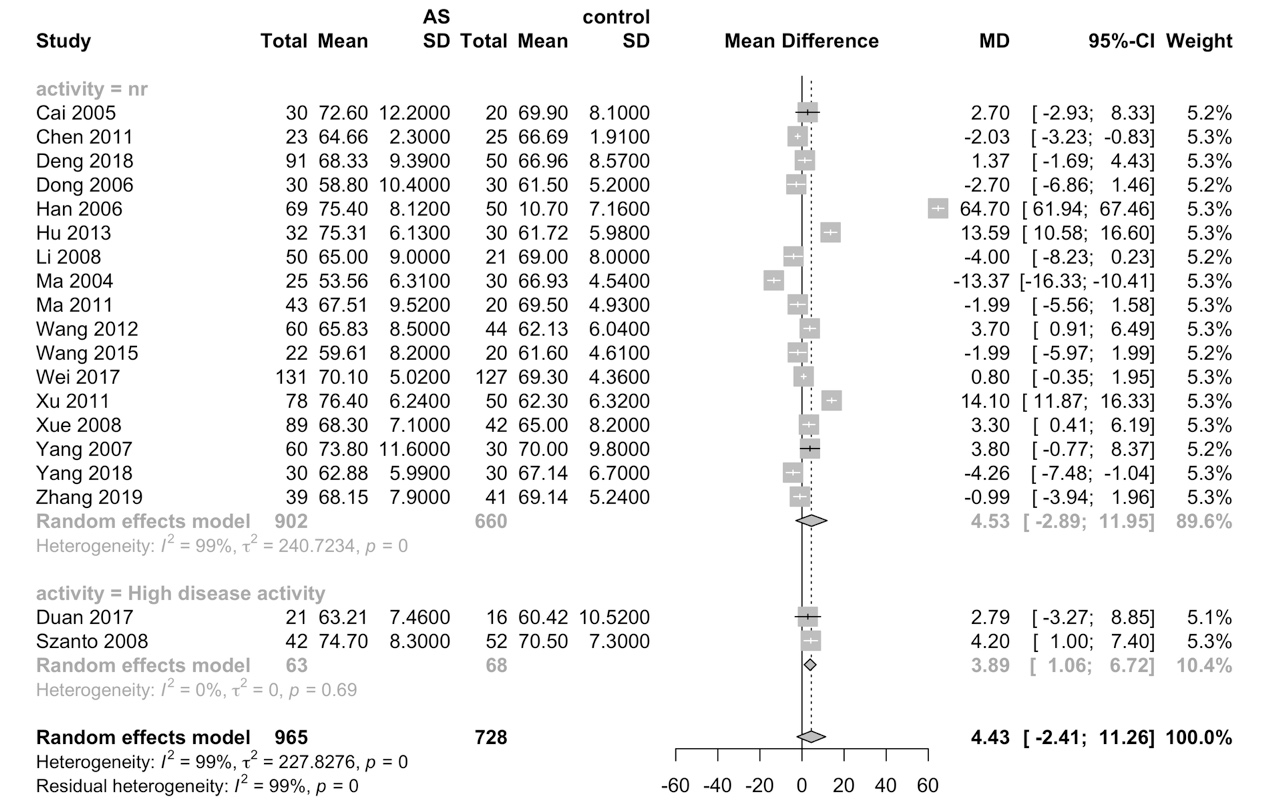


Figure 3. Subgroup analysis of proportions of CD3+ T cells in peripheral blood in AS patients by disease activity


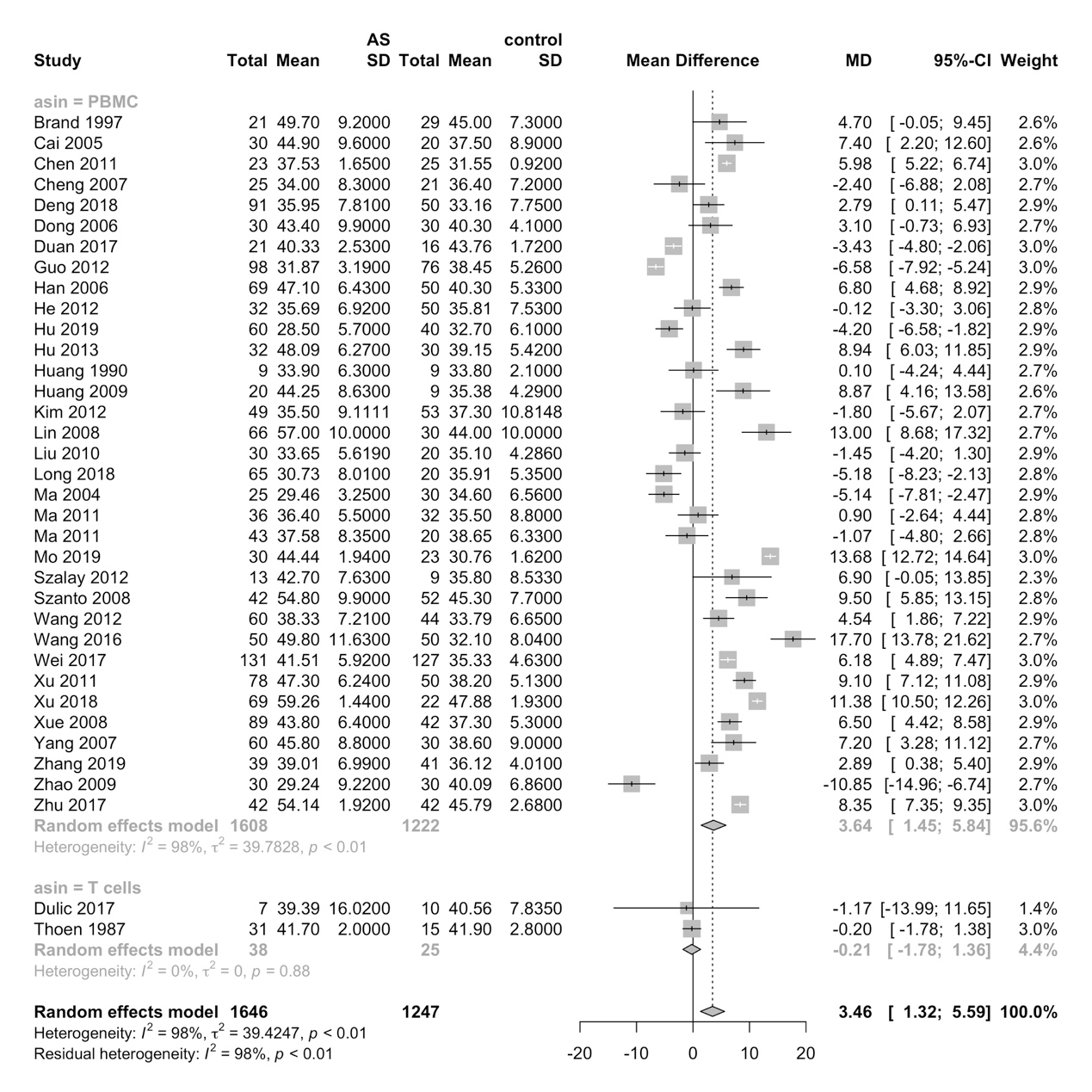


Figure 4. Proportions of CD4+ T cells in peripheral blood in AS patients


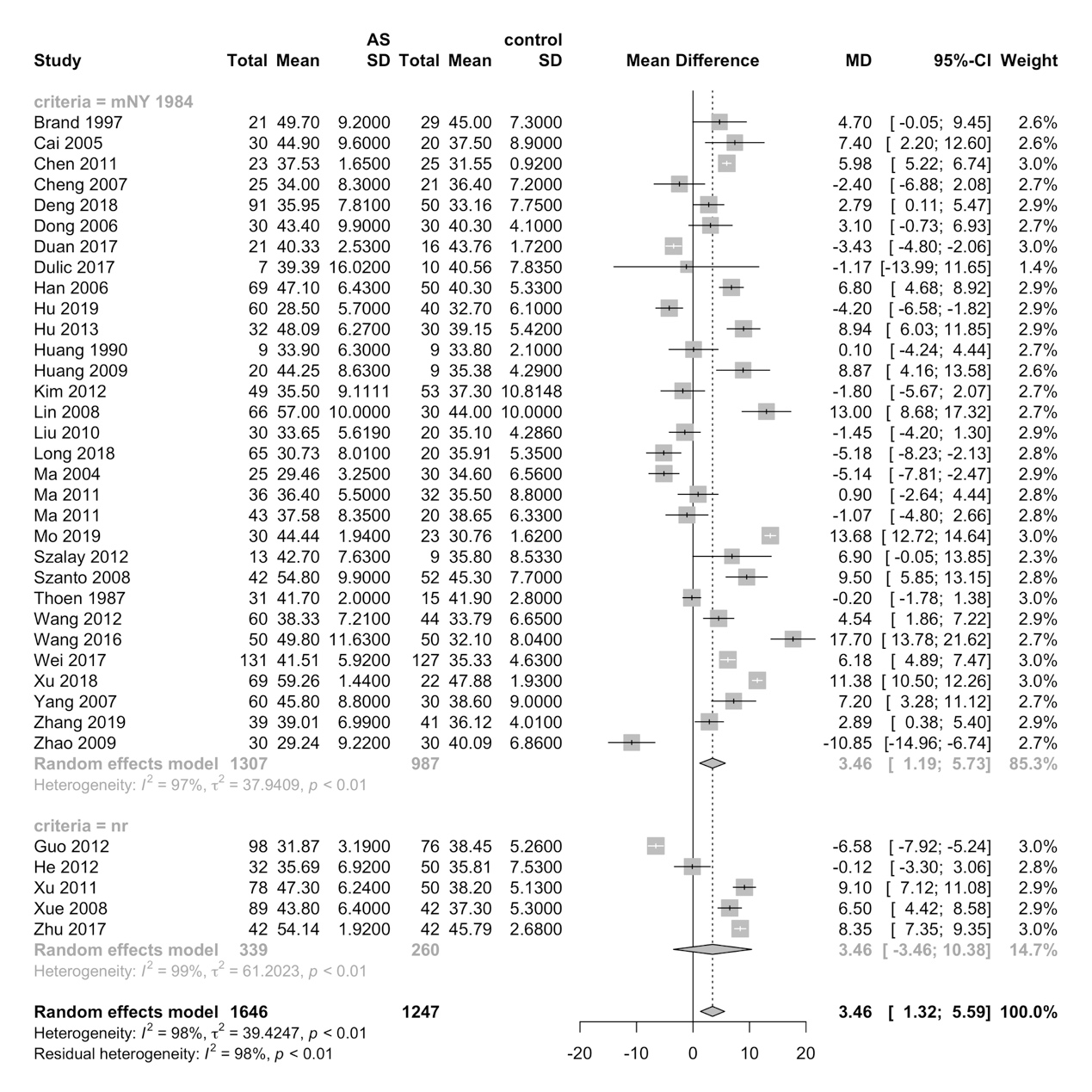


Figure 5. Subgroup analysis of proportions of CD4+ T cells in peripheral blood in AS patients by classification criteria


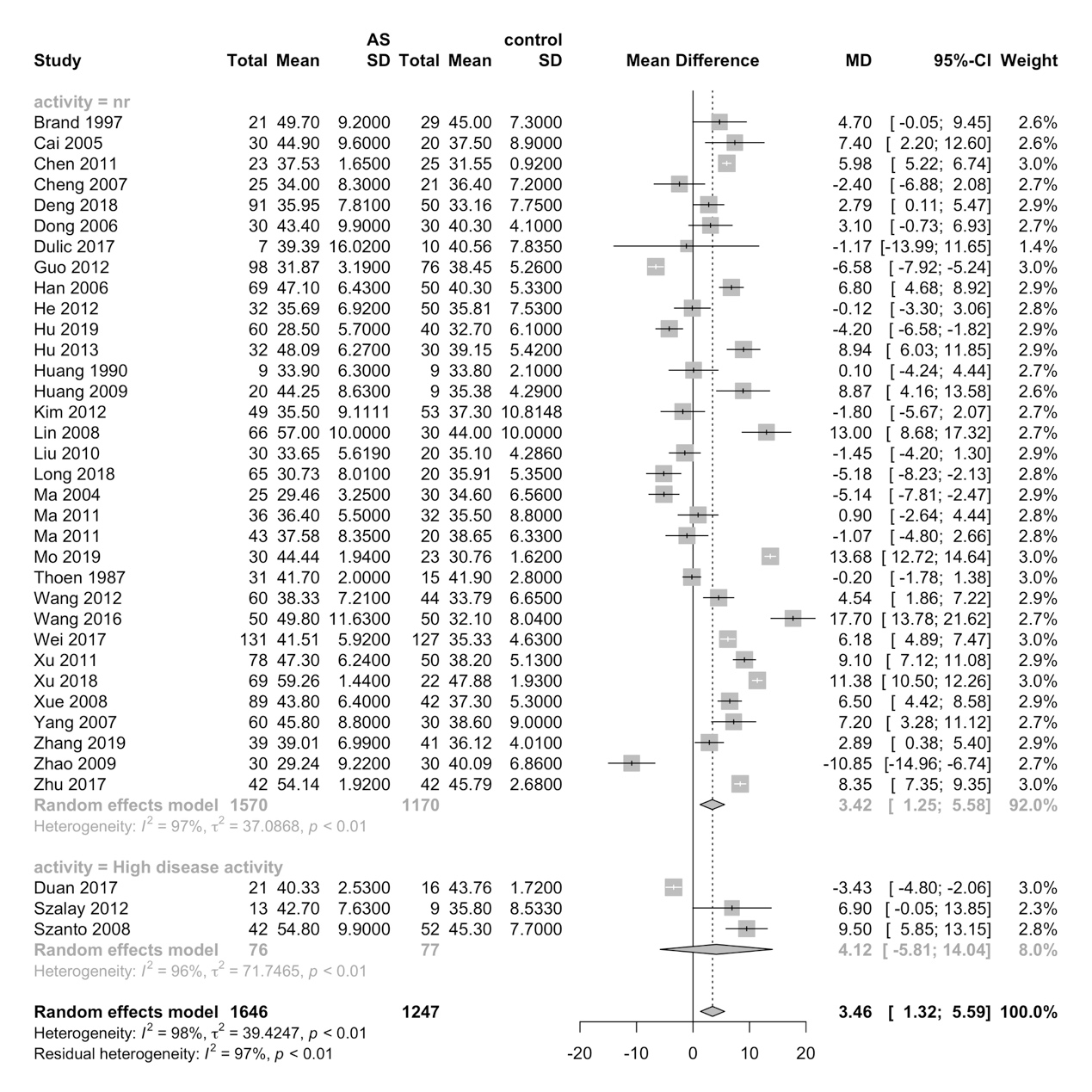


Figure 6. Subgroup analysis of proportions of CD4+ T cells in peripheral blood in AS patients by disease activity


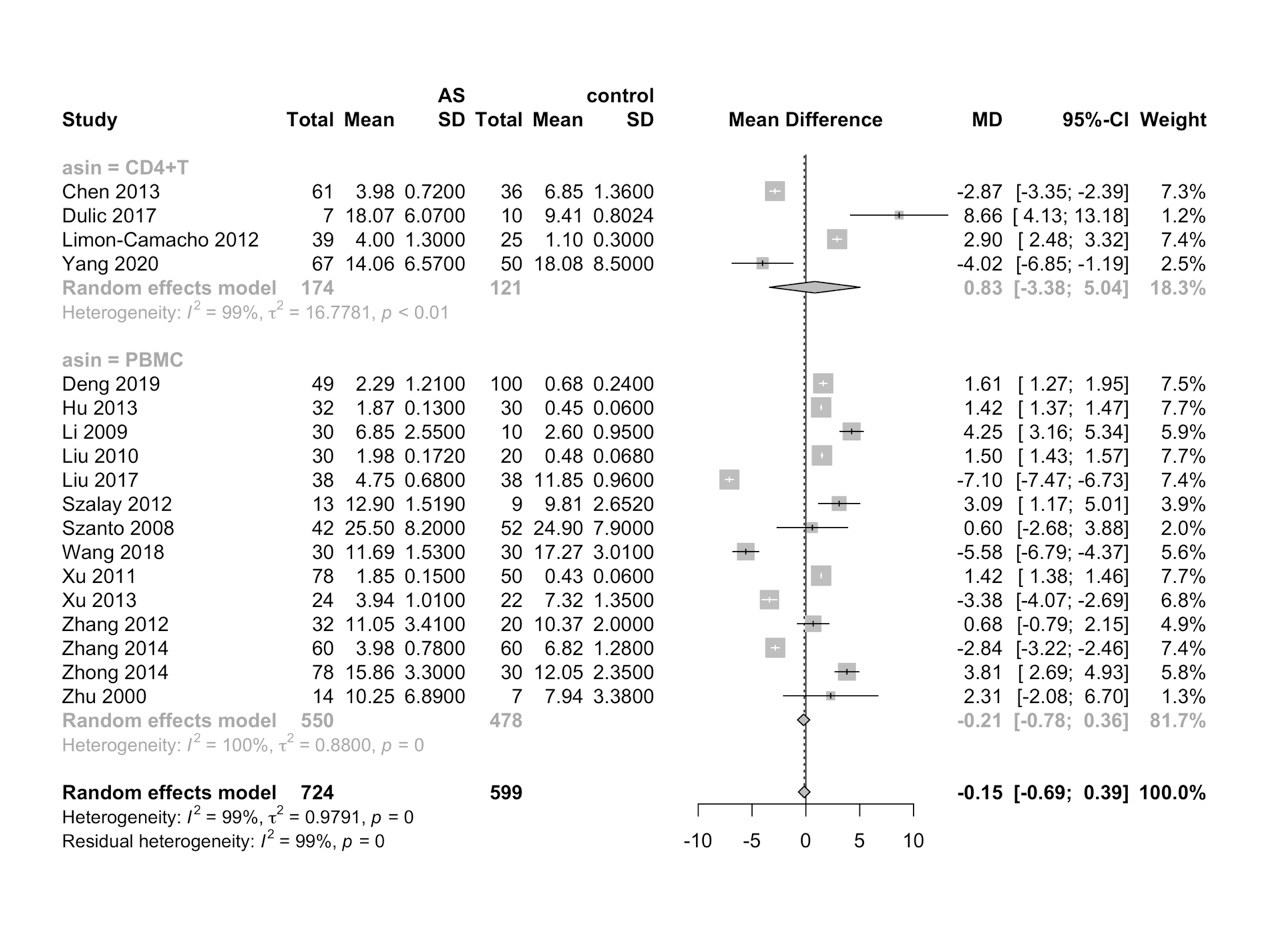


Figure 7. Proportions of Th1 cells in peripheral blood in AS patients


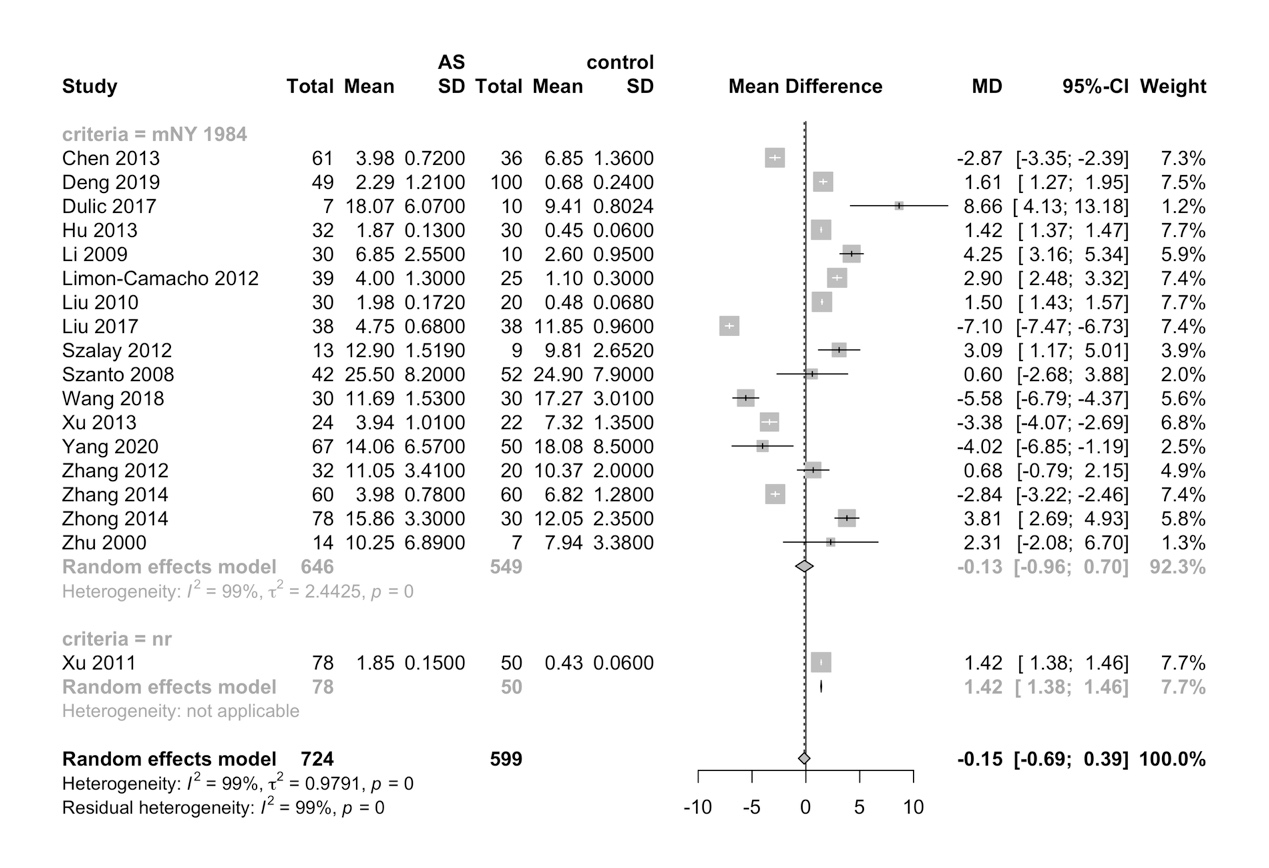


Figure 8. Subgroup analysis of proportions of Th1 cells in peripheral blood in AS patients by classification criteria


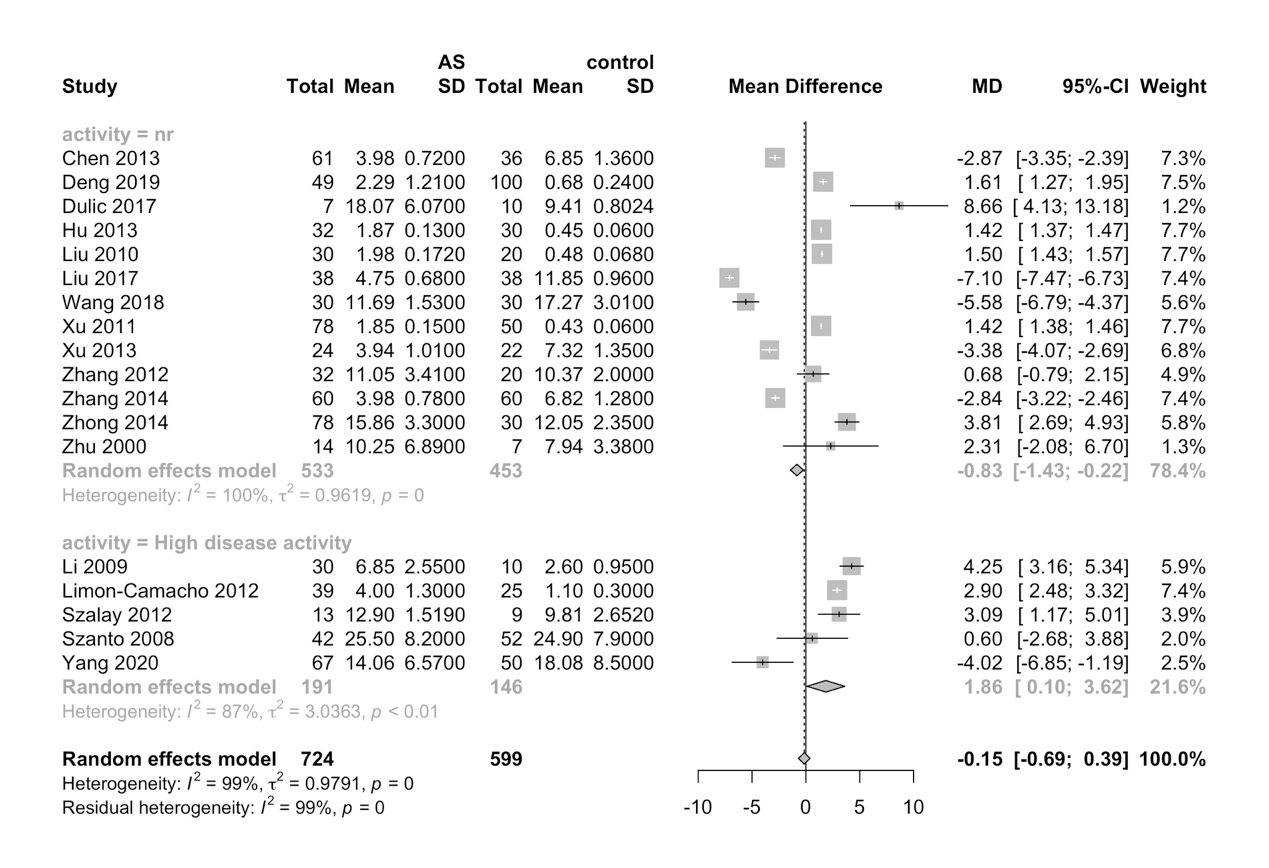


Figure 9. Subgroup analysis of proportions of Th1 cells in peripheral blood in AS patients by disease activity


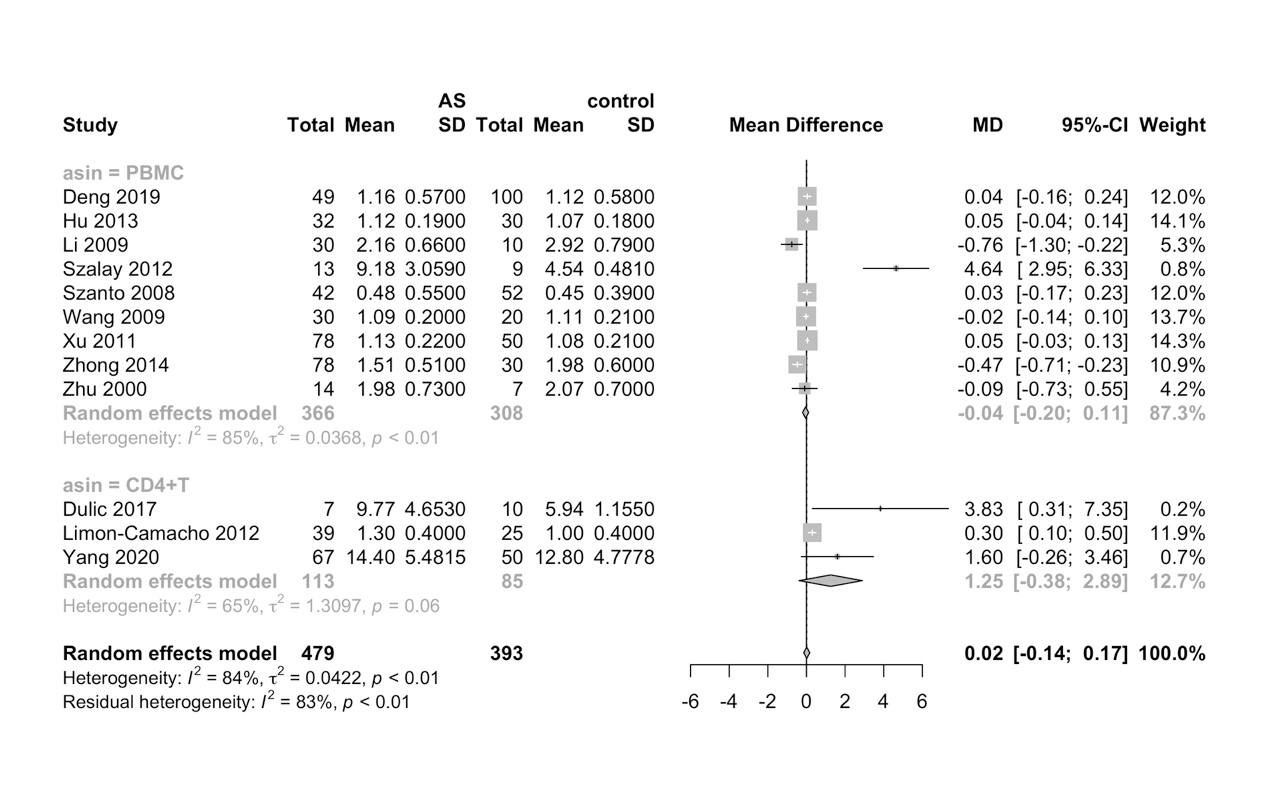


Figure 10. Proportions of Th2 cells in peripheral blood in AS patients


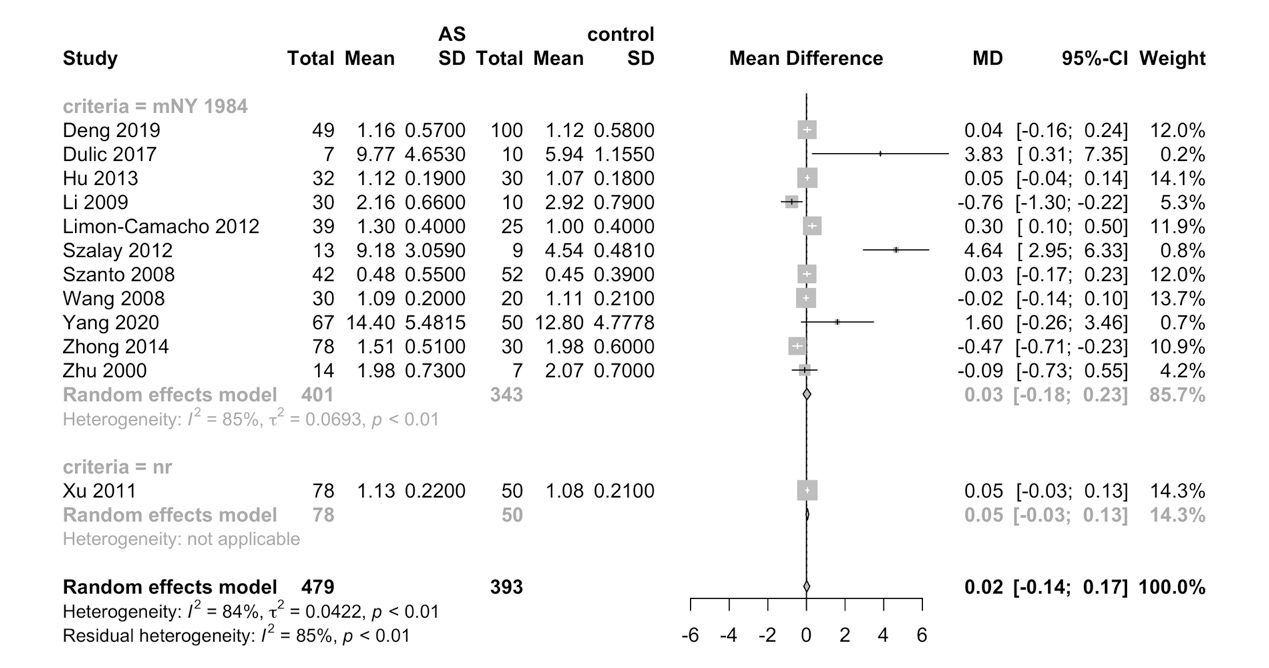


Figure 11. Subgroup analysis of proportions of Th2 cells in peripheral blood in AS patients by classification criteria


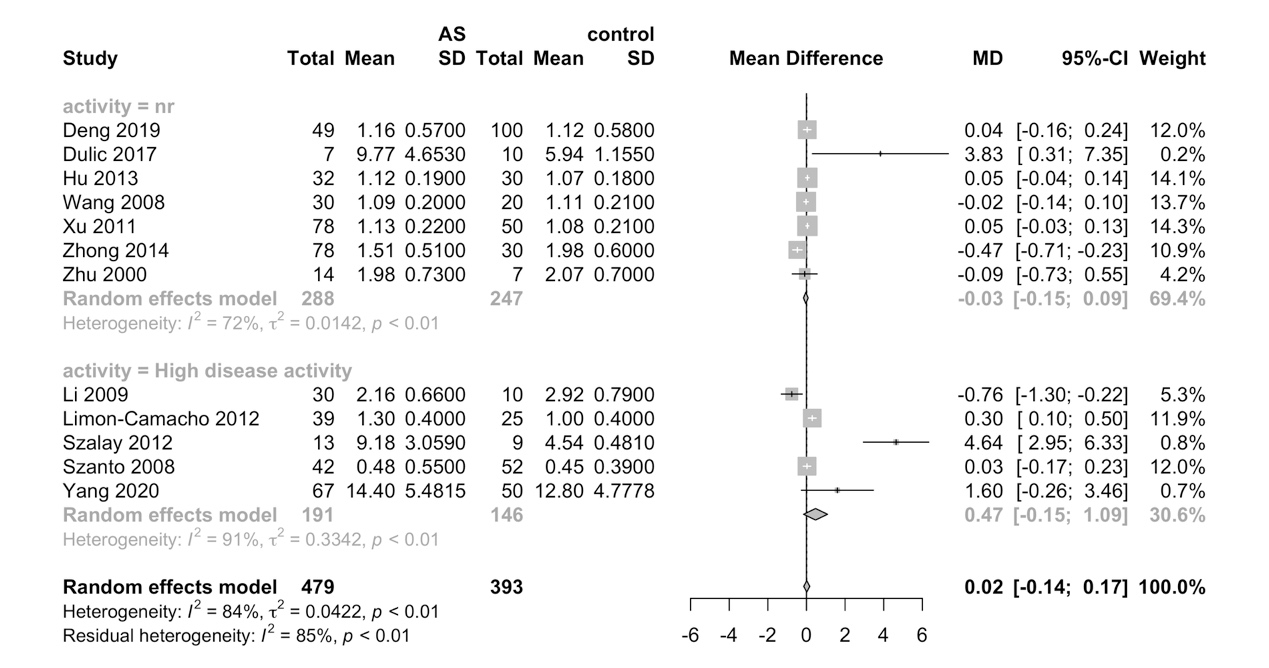


Figure 12. Subgroup analysis of proportions of Th2 cells in peripheral blood in AS patients by disease activity


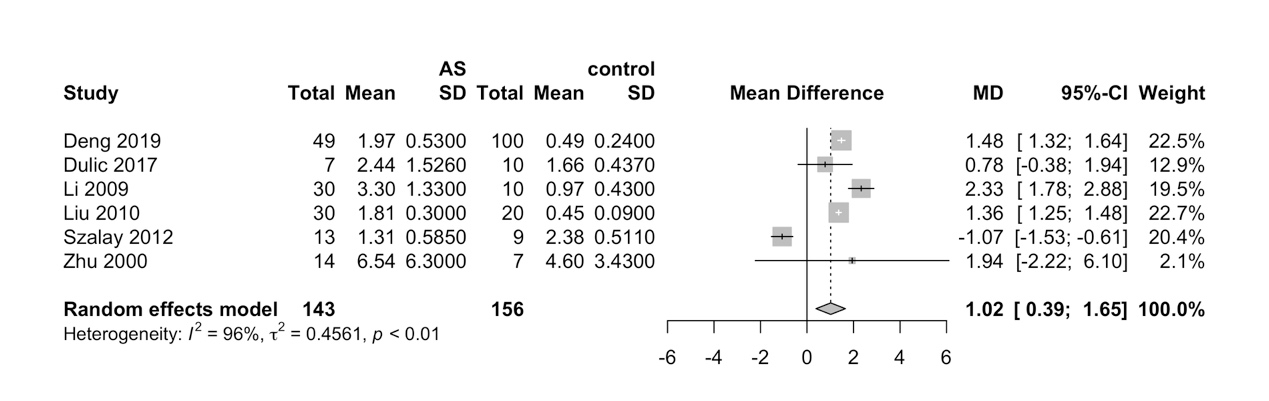


Figure 13. Th1/Th2 ratio in peripheral blood in AS patients


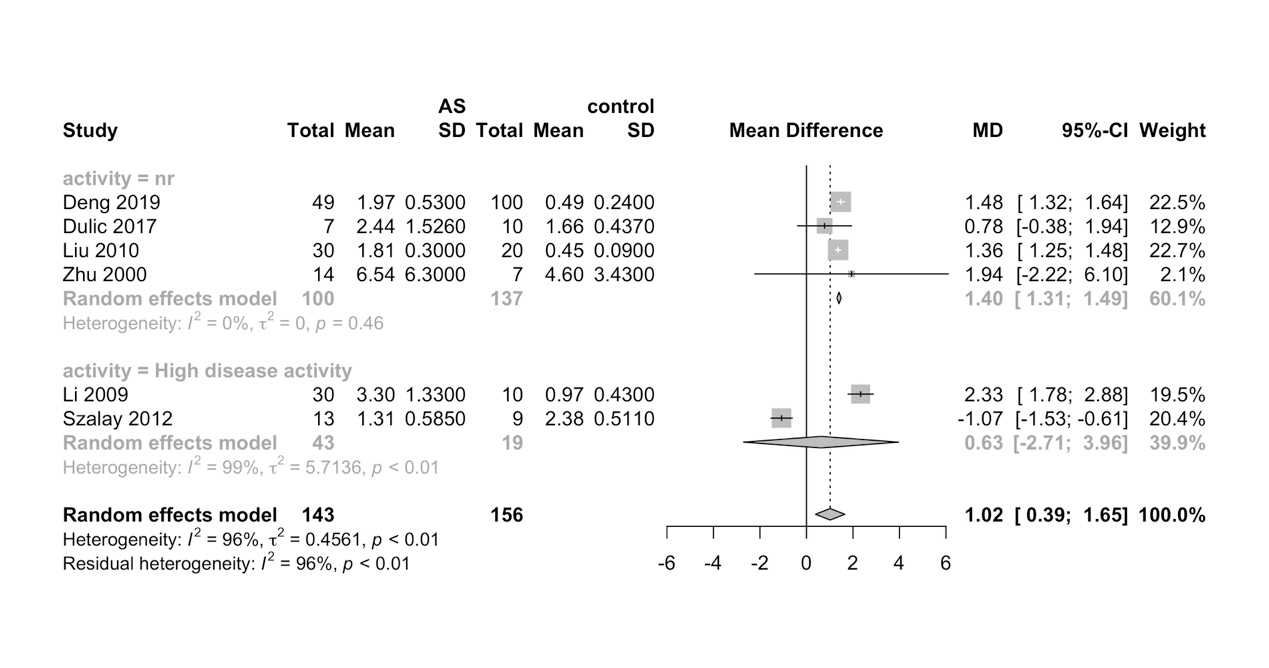


Figure 14. Subgroup analysis of Th1/Th2 ratio in peripheral blood in AS patients by disease activity


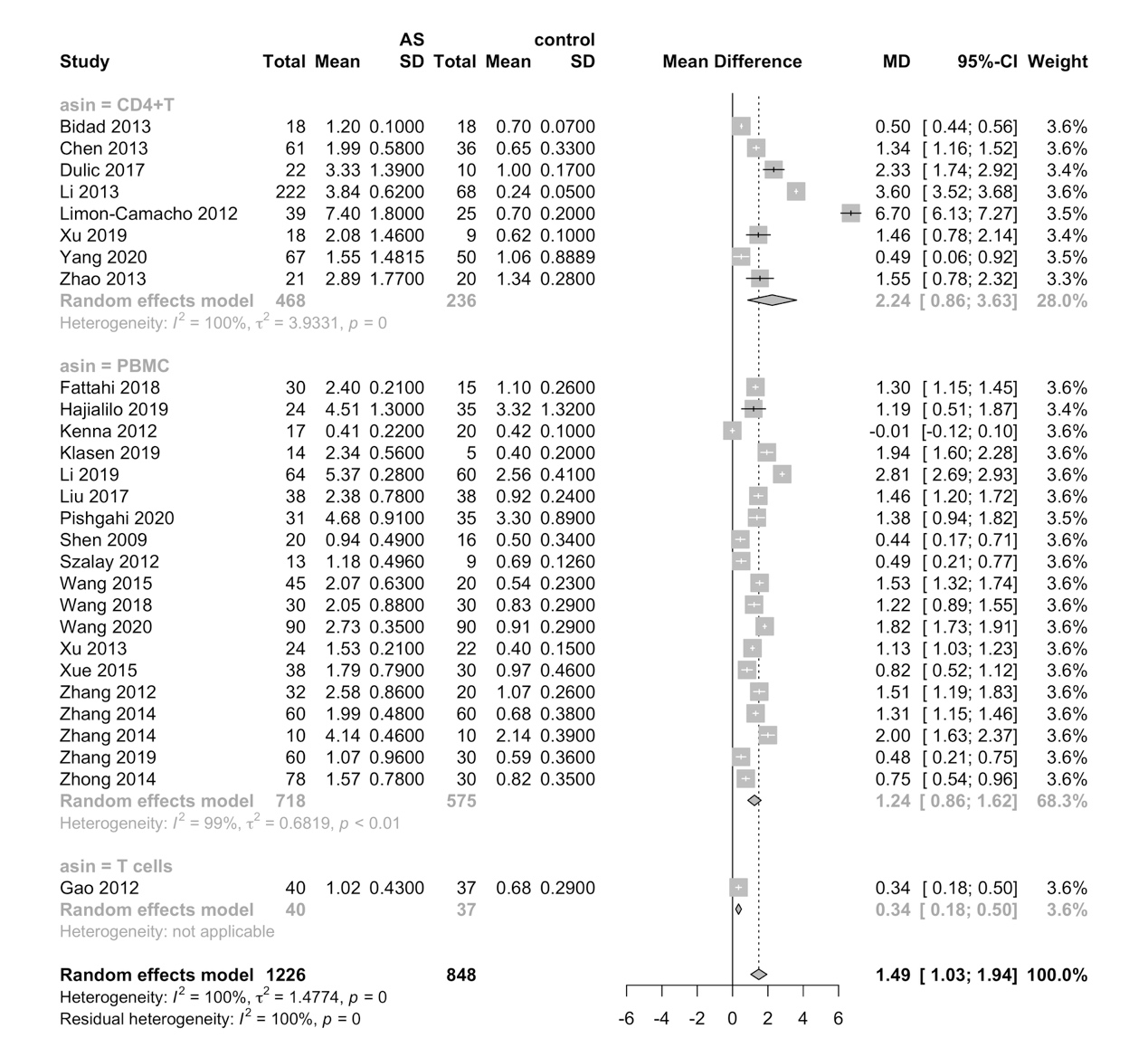


Figure 15. Proportions of Th17 cells in peripheral blood in AS patients


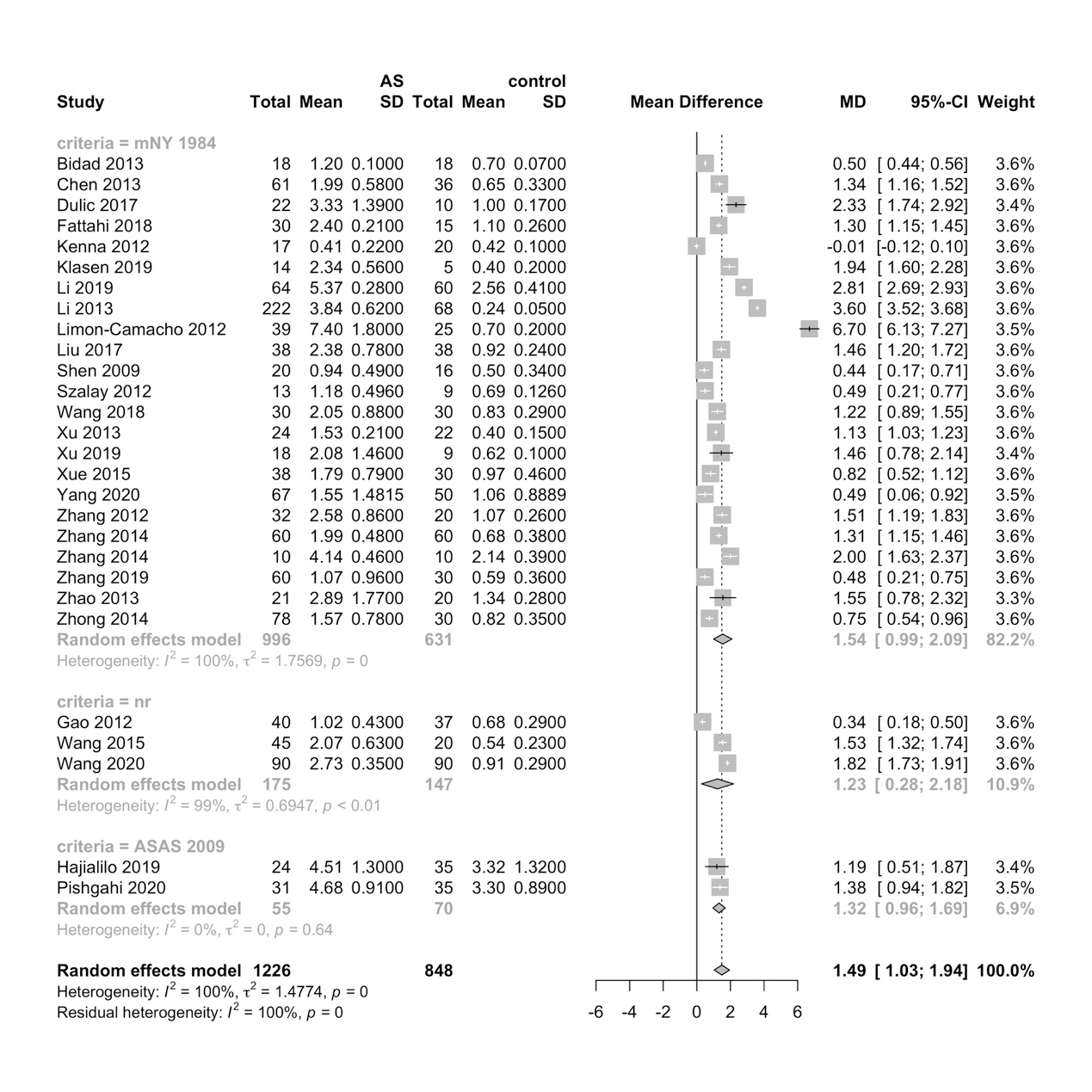


Figure 16. Subgroup analysis of proportions of Th17 cells in peripheral blood in AS patients by classification criteria


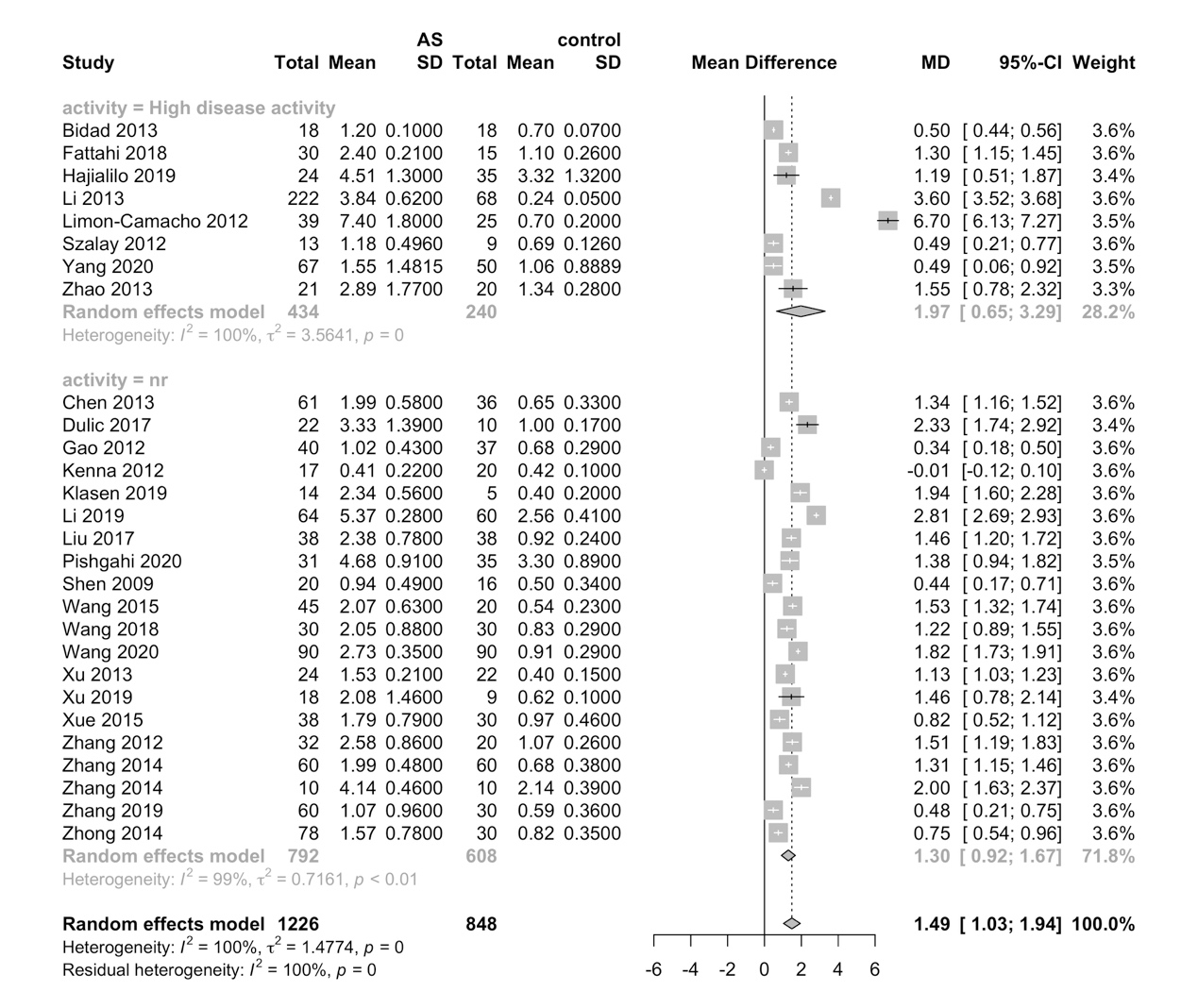


Figure 17. Subgroup analysis of proportions of Th17 cells in peripheral blood in AS patients by disease activity


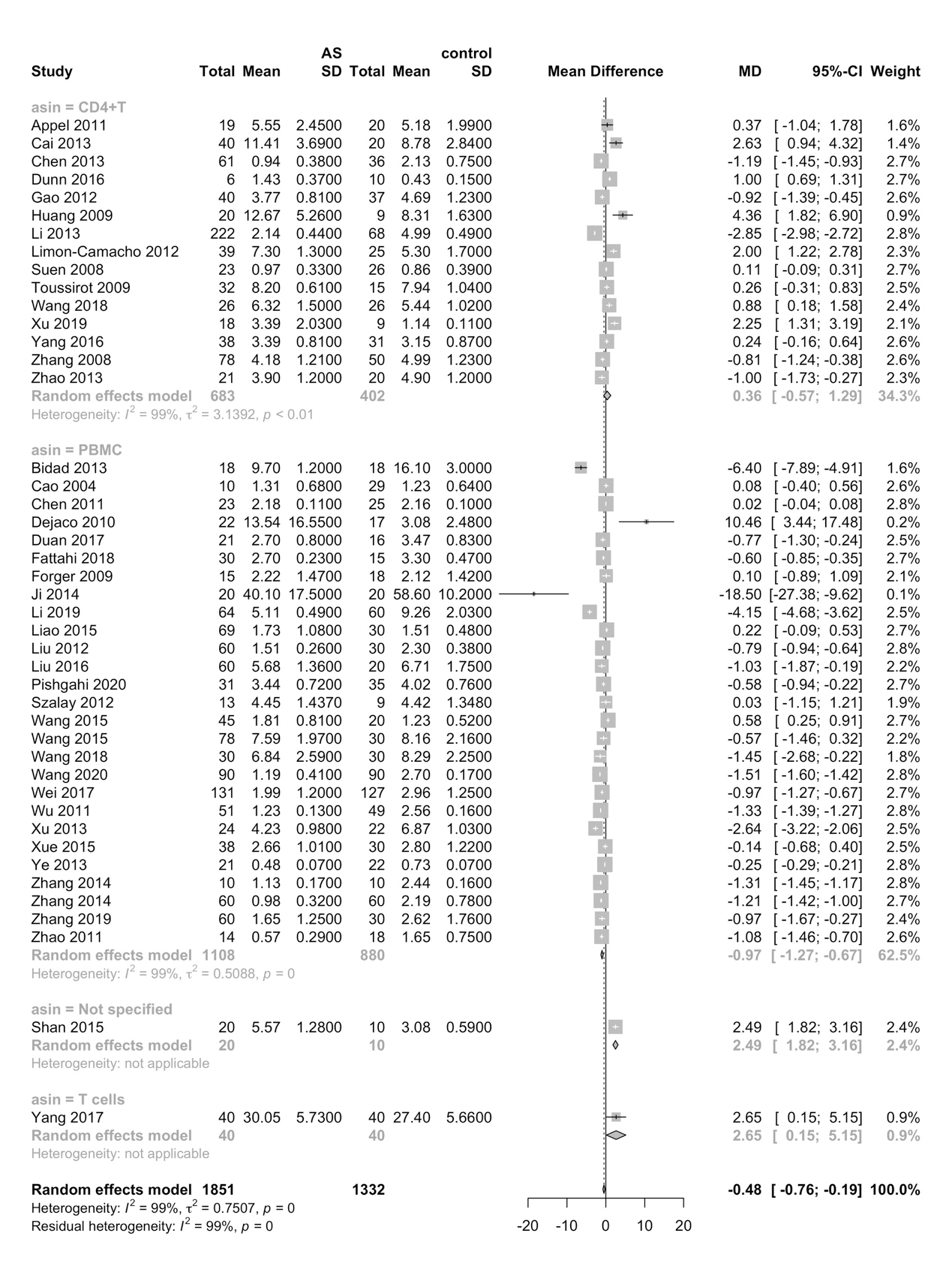


Figure 18. Proportions of Tregs in peripheral blood in AS patients


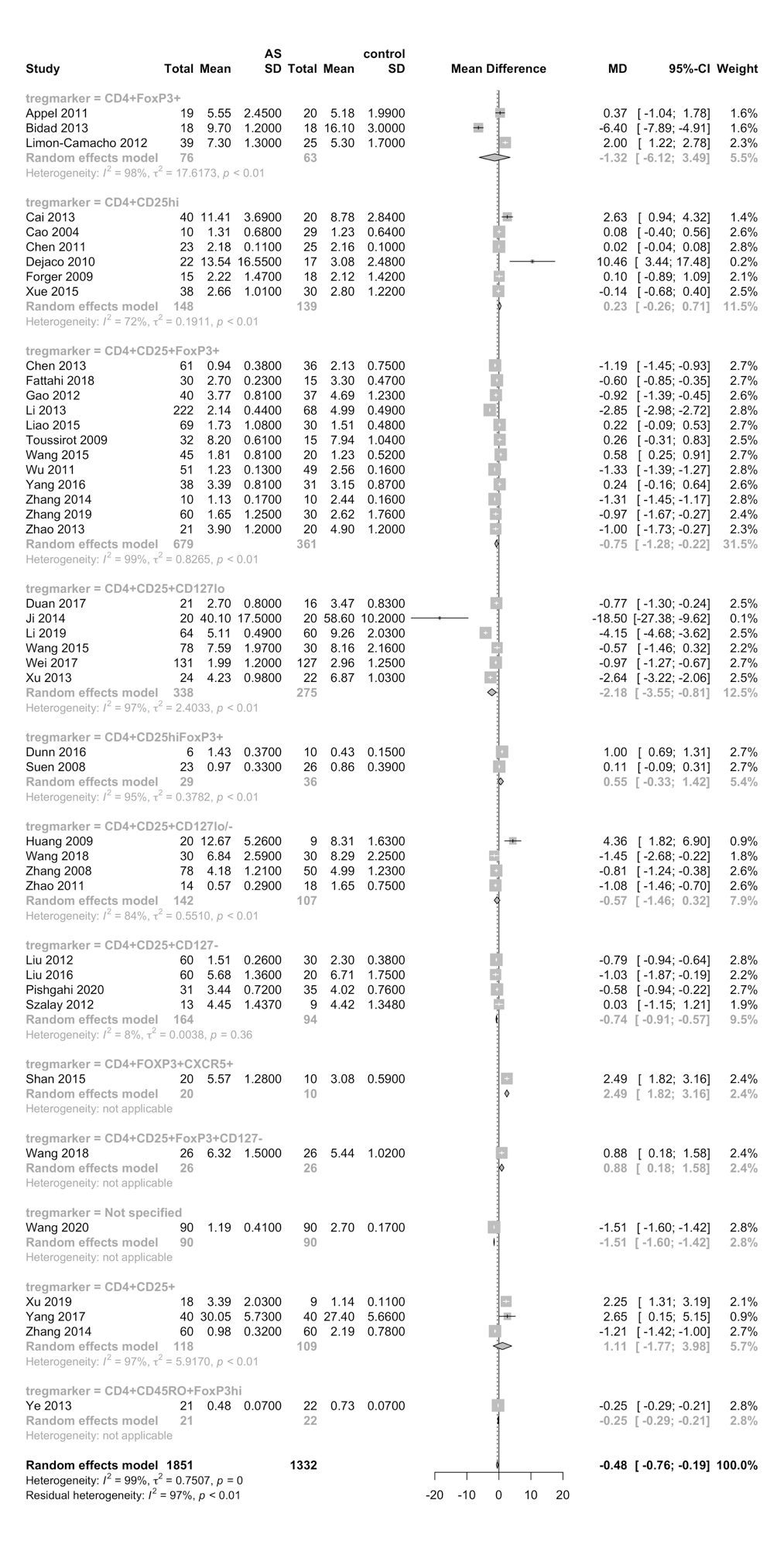


Figure 19. Proportions of Tregs by different markers in peripheral blood in AS patients


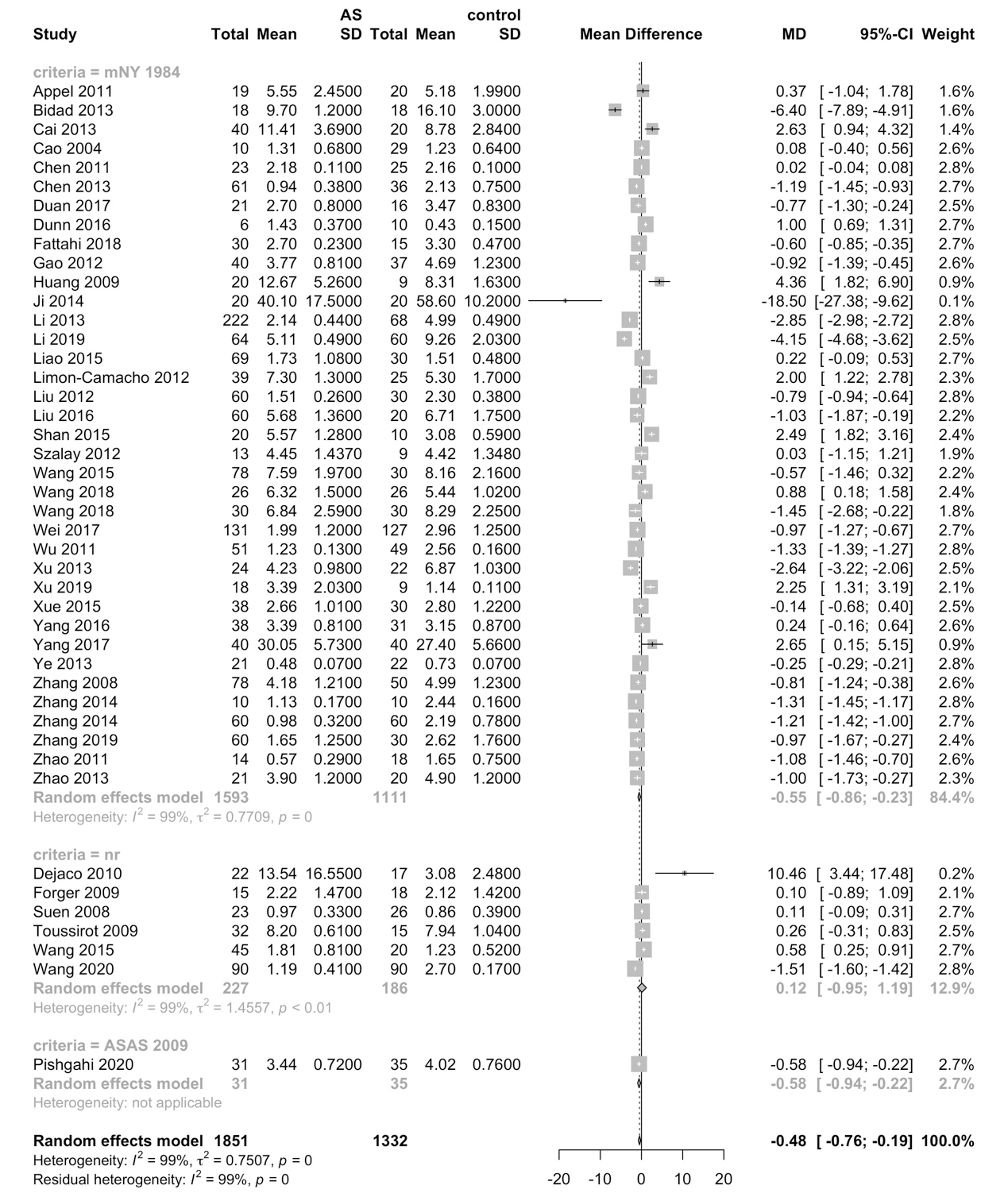


Figure 20. Subgroup analysis of proportions of Tregs in peripheral blood in AS patients by classification criteria


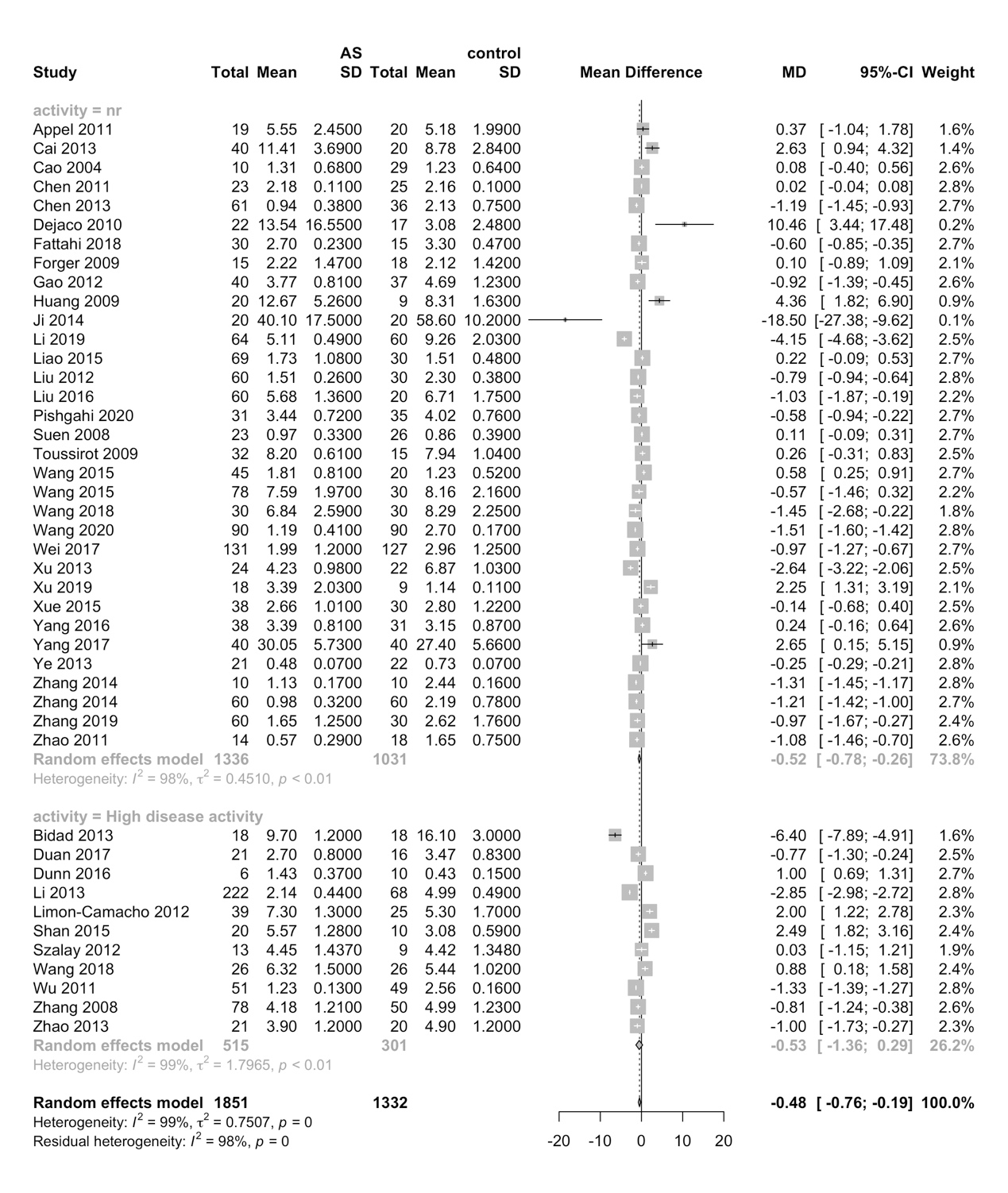


Figure 21. Subgroup analysis of proportions of Tregs in peripheral blood in AS patients by disease activity


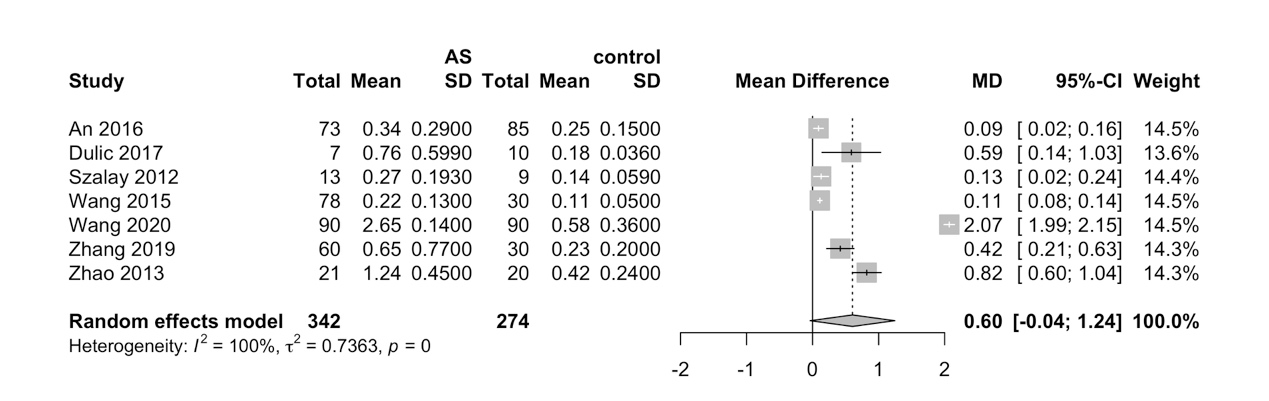


Figure 22. Th17/Tregs ratio in peripheral blood in AS patients


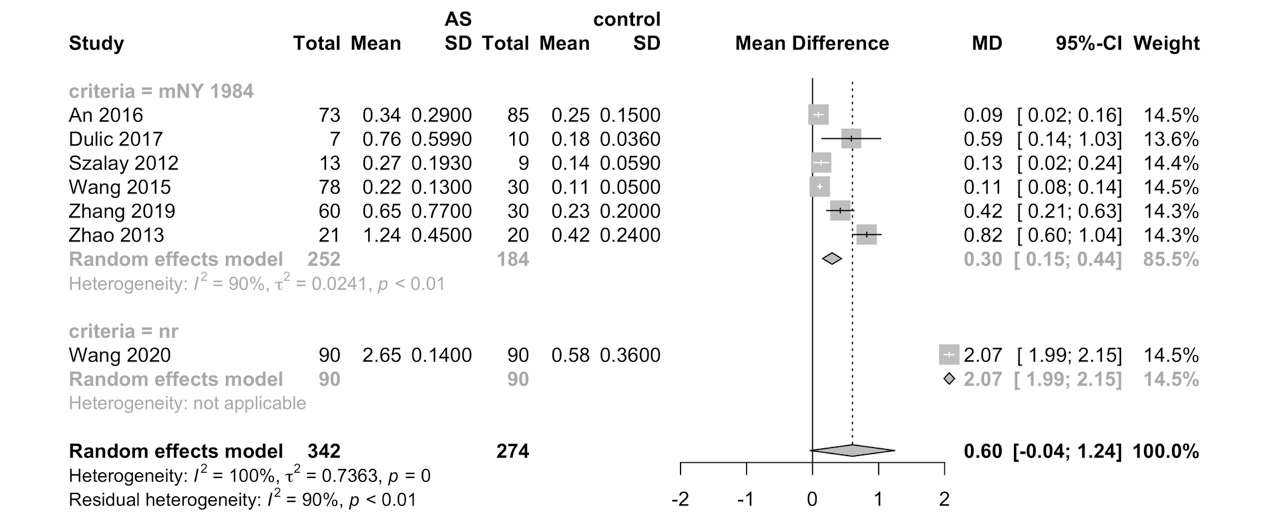


Figure 23. Subgroup analysis of proportions of Th17/Tregs ratio in peripheral blood in AS patients by classification criteria


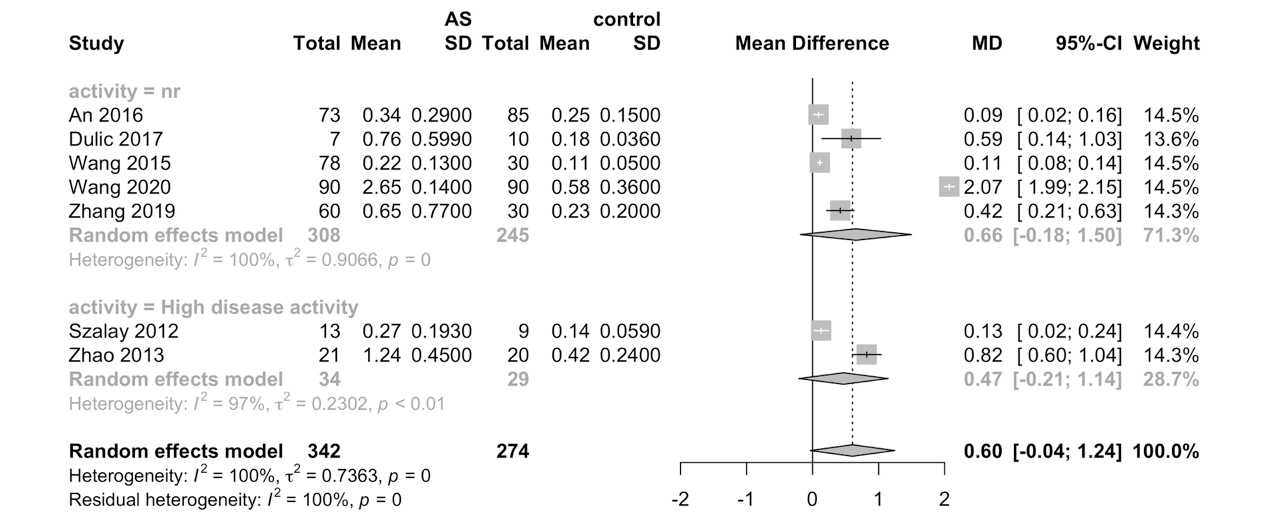


Figure 24. Subgroup analysis of proportions of Th17/Tregs ratio in peripheral blood in AS patients by disease activity


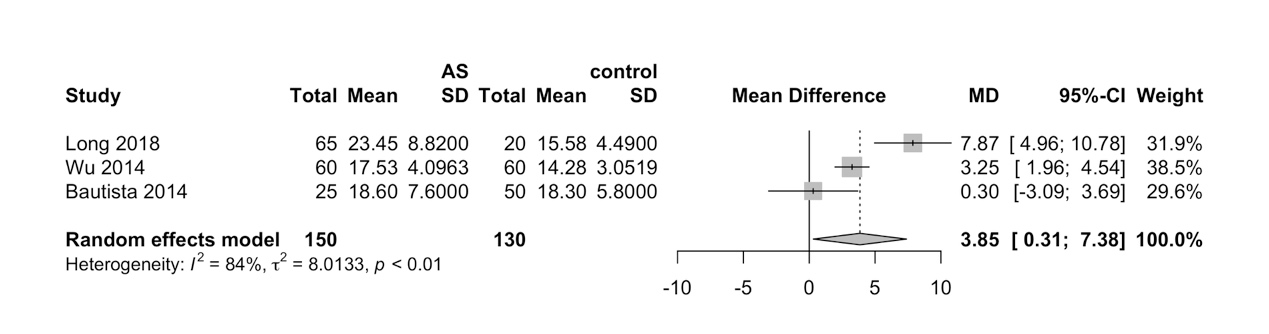


Figure 25. Proportions of Tfh cells in peripheral blood in AS patients


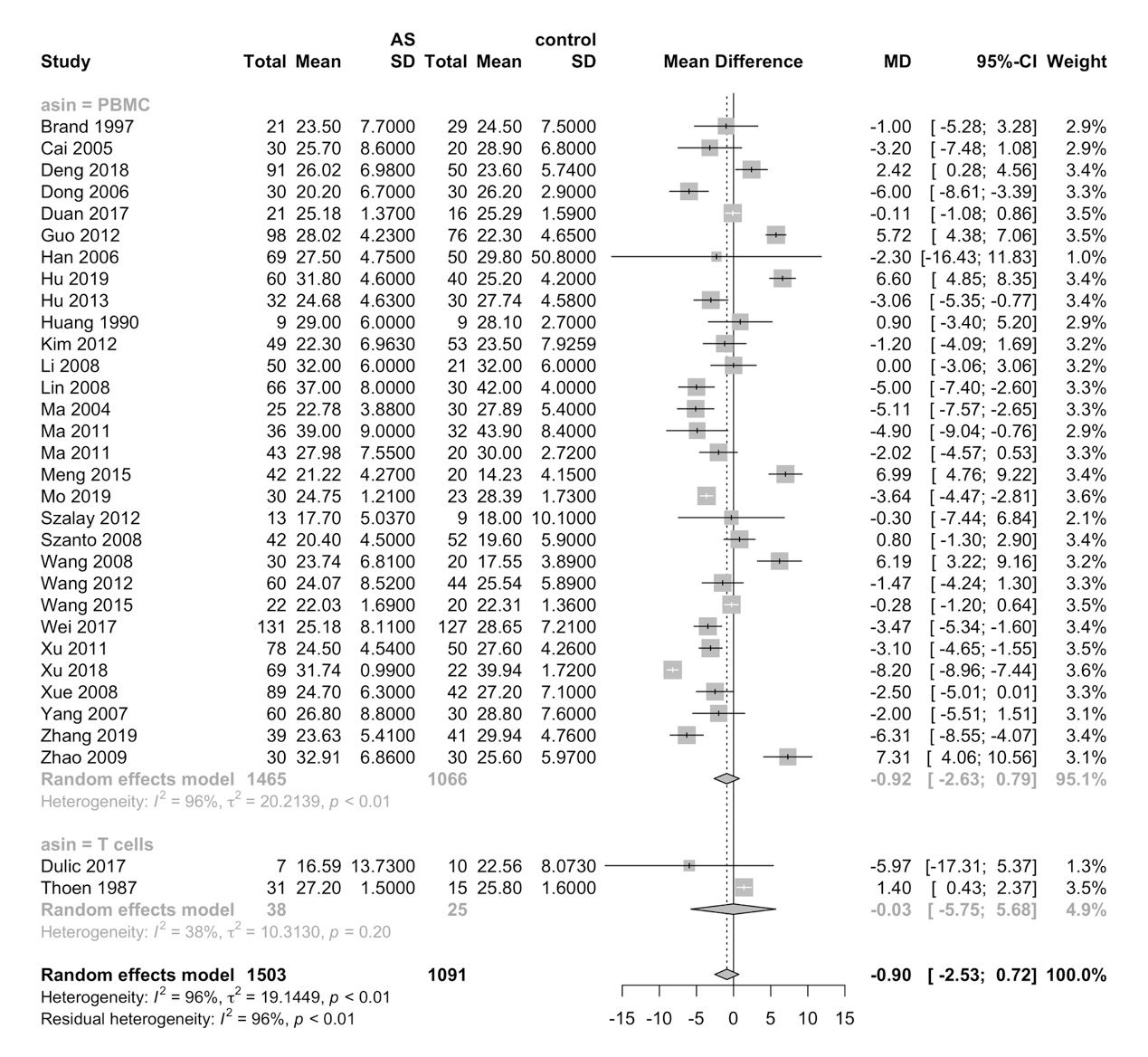


Figure 26. Proportions of CD8+ T cells in peripheral blood in AS patients


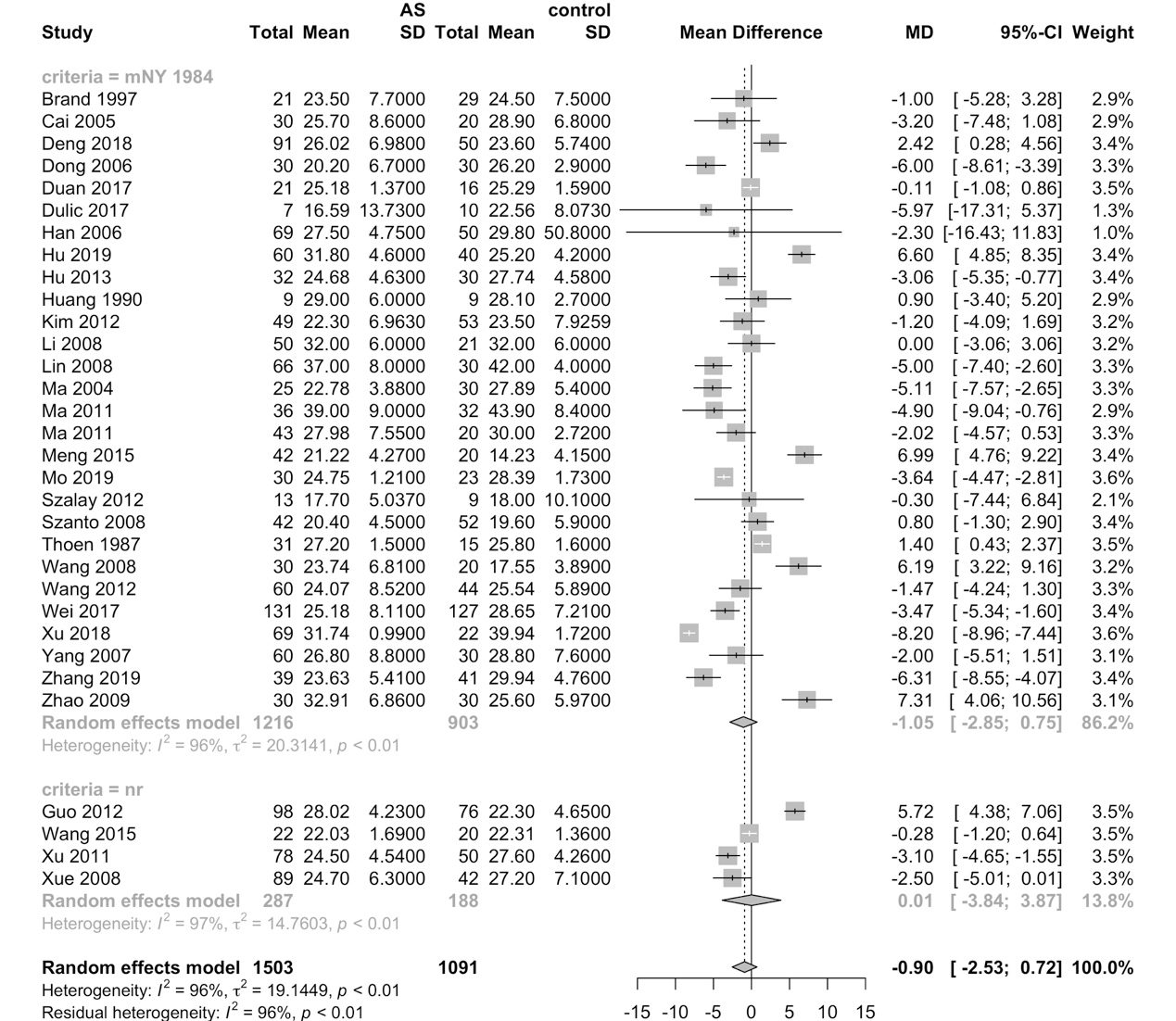


Figure 27. Subgroup analysis of proportions of CD8+ T lymphocytes in peripheral blood in AS patients by classification criteria


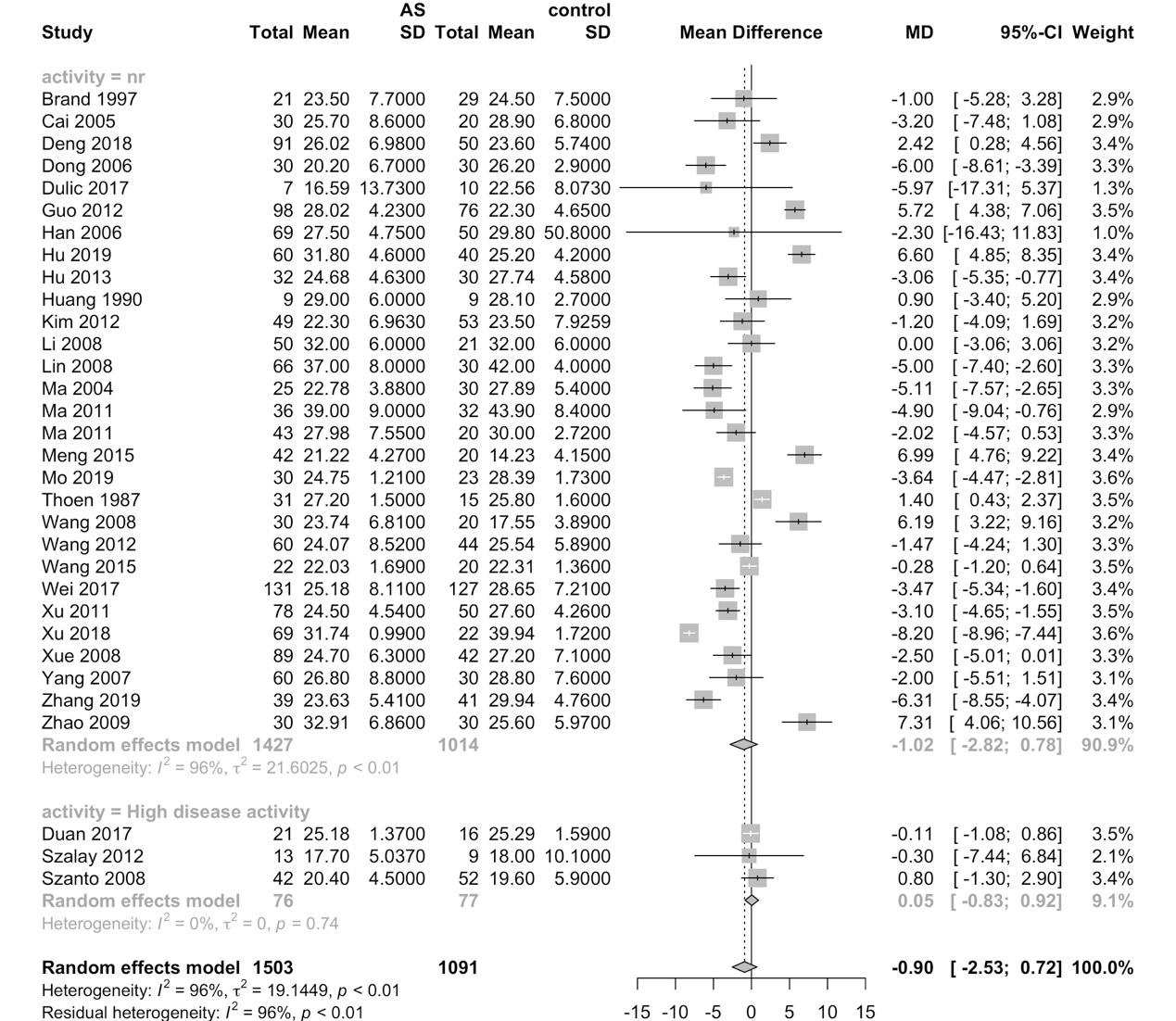


Figure 28. Subgroup analysis of proportions of CD8+ T cells in peripheral blood in AS patients by disease activity


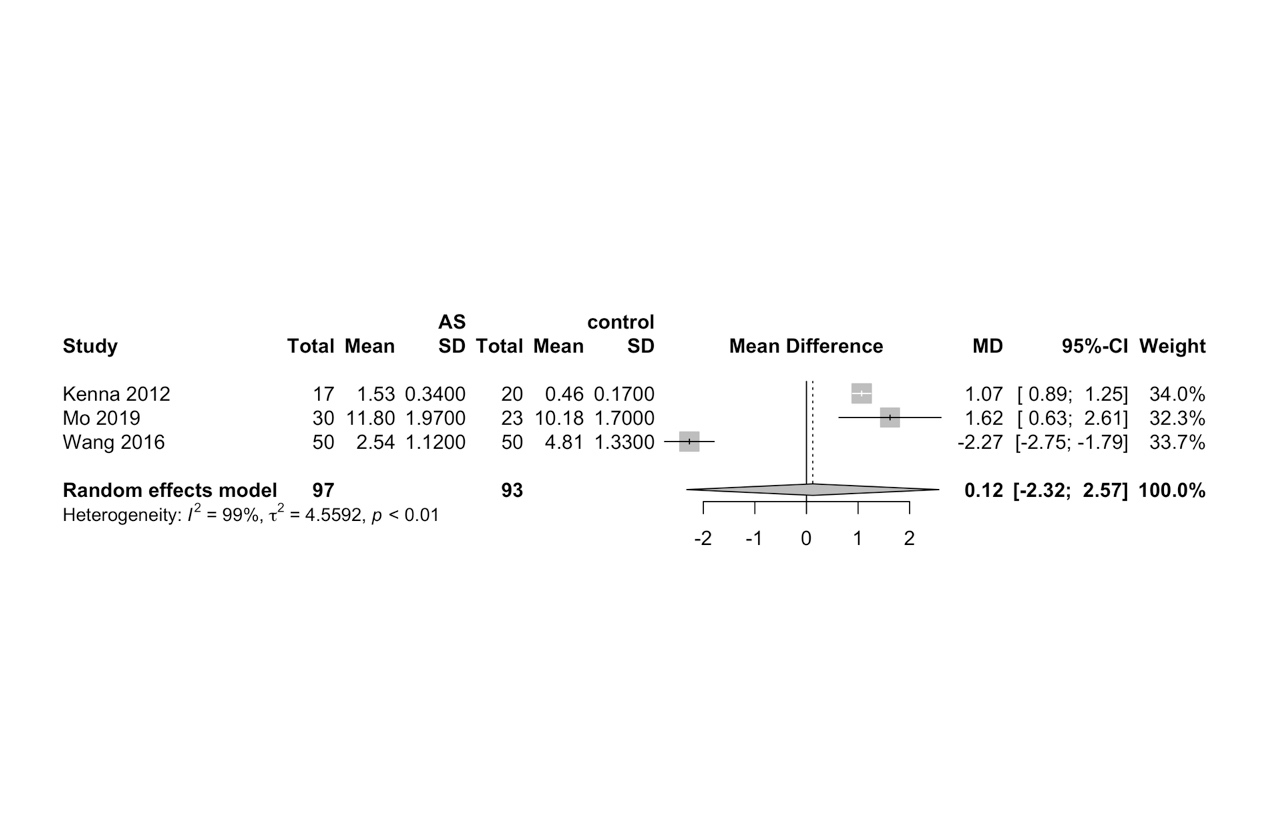


Figure 29. Proportions of γδ T cells in peripheral blood in AS patients


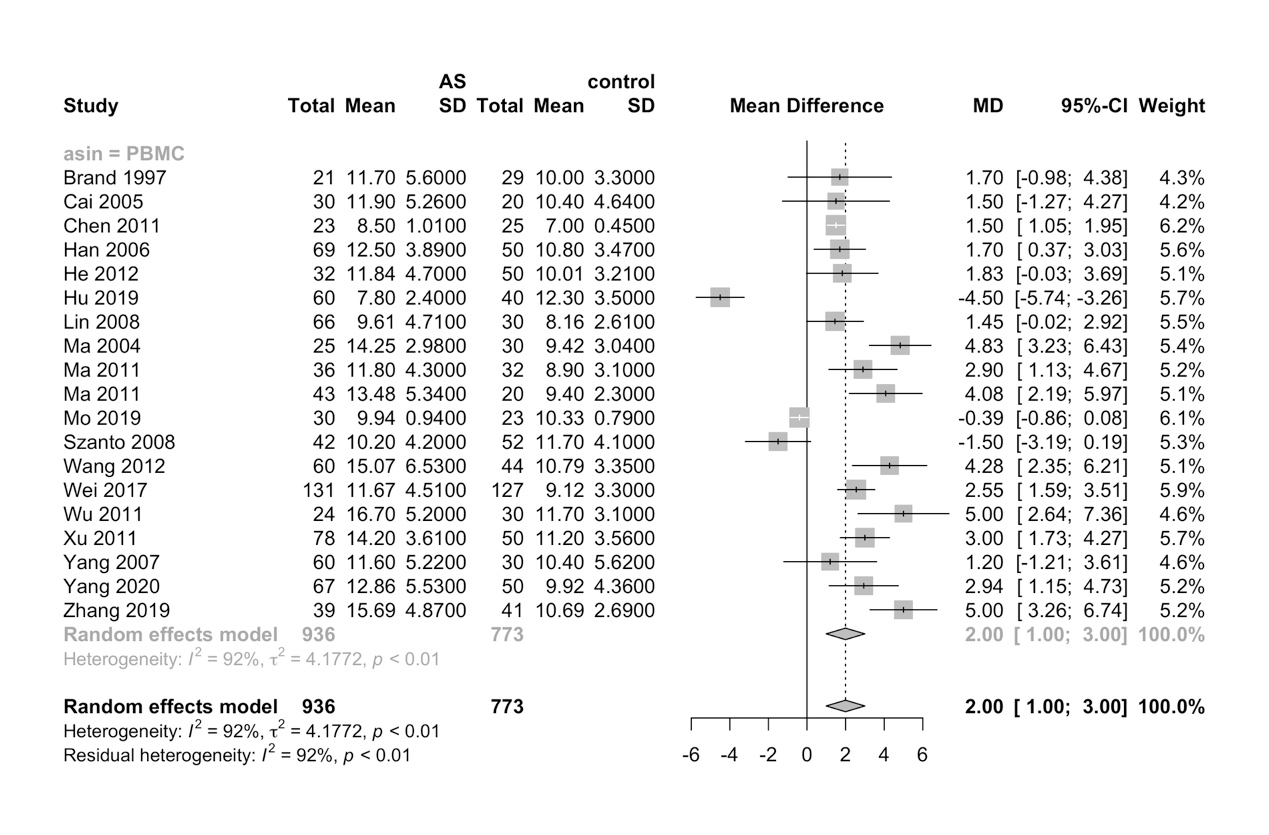


Figure 30. Proportions of B cells in peripheral blood in AS patients


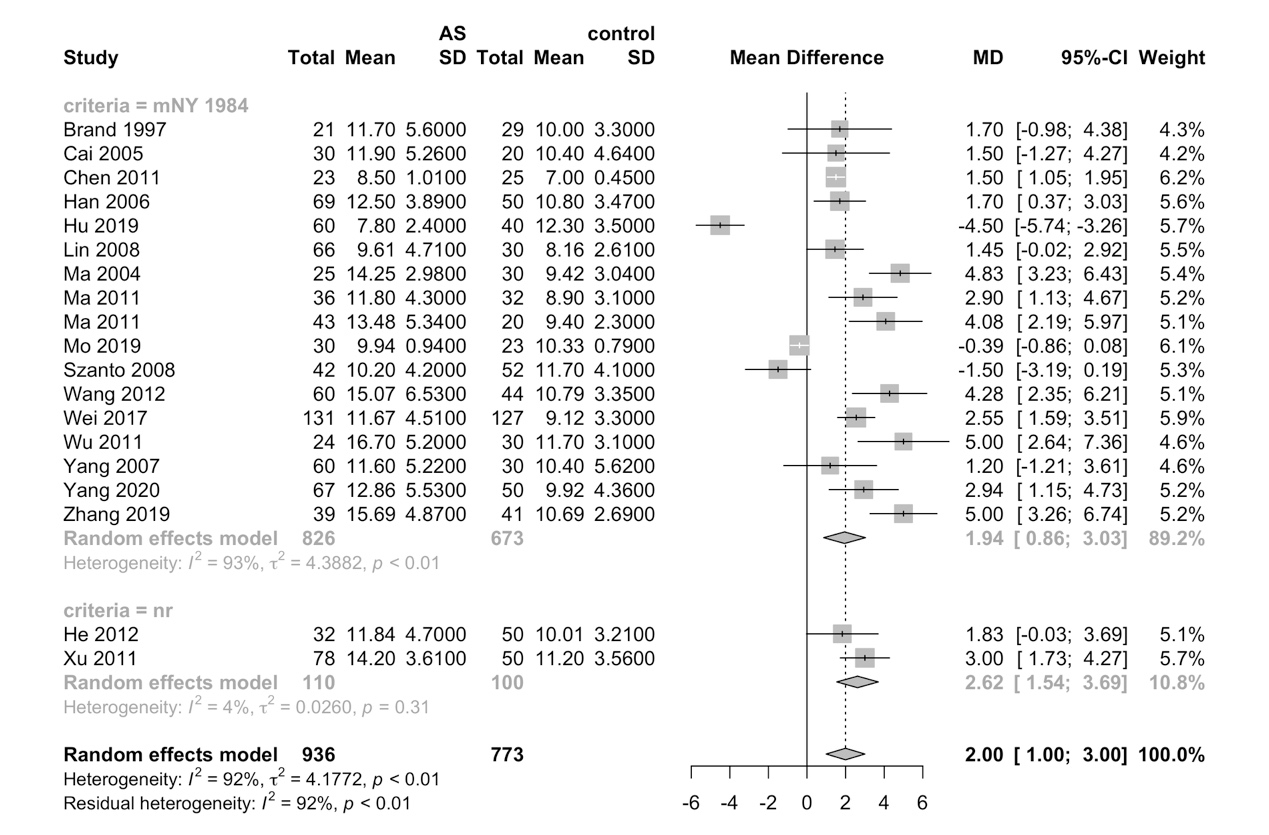


Figure 31. Subgroup analysis of proportions of B lymphocytes in peripheral blood in AS patients by classification criteria


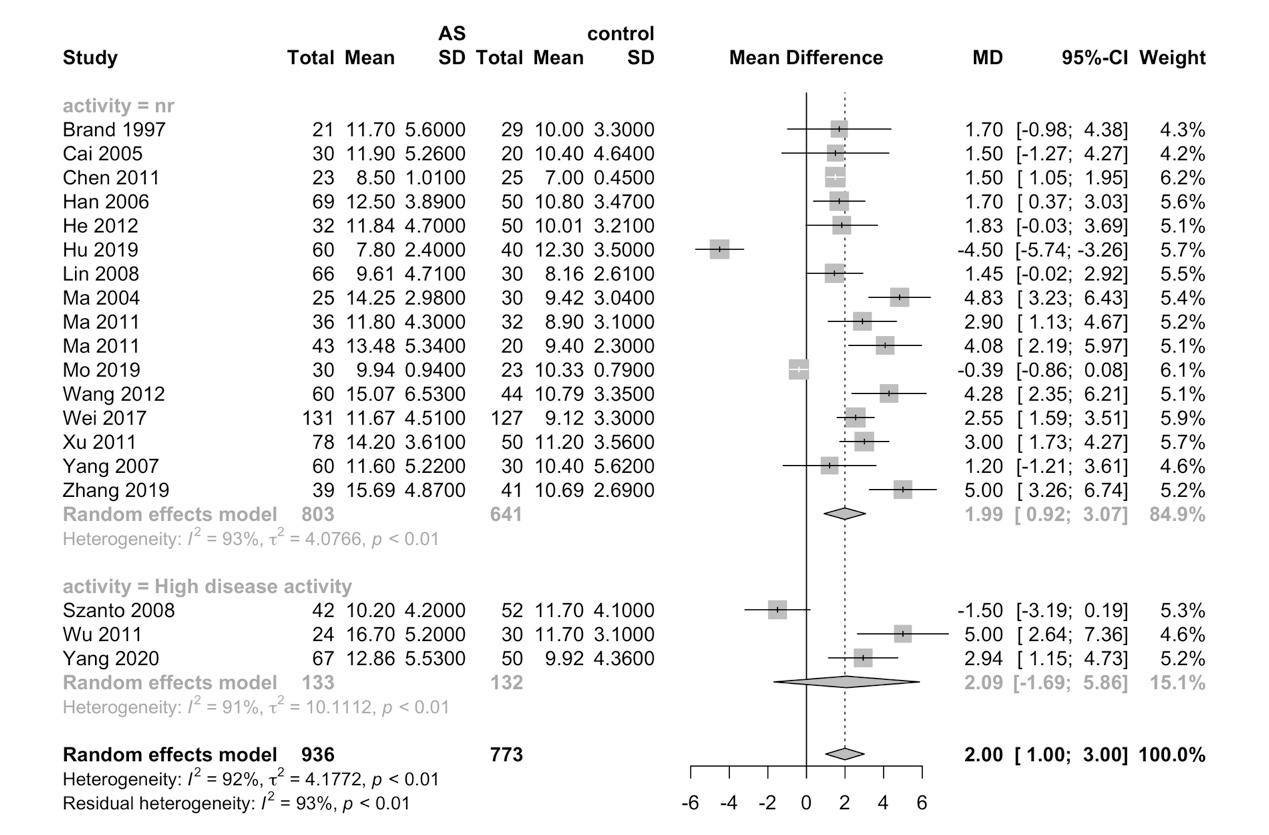


Figure 32. Subgroup analysis of proportions of B lymphocytes in peripheral blood in AS patients by disease activity


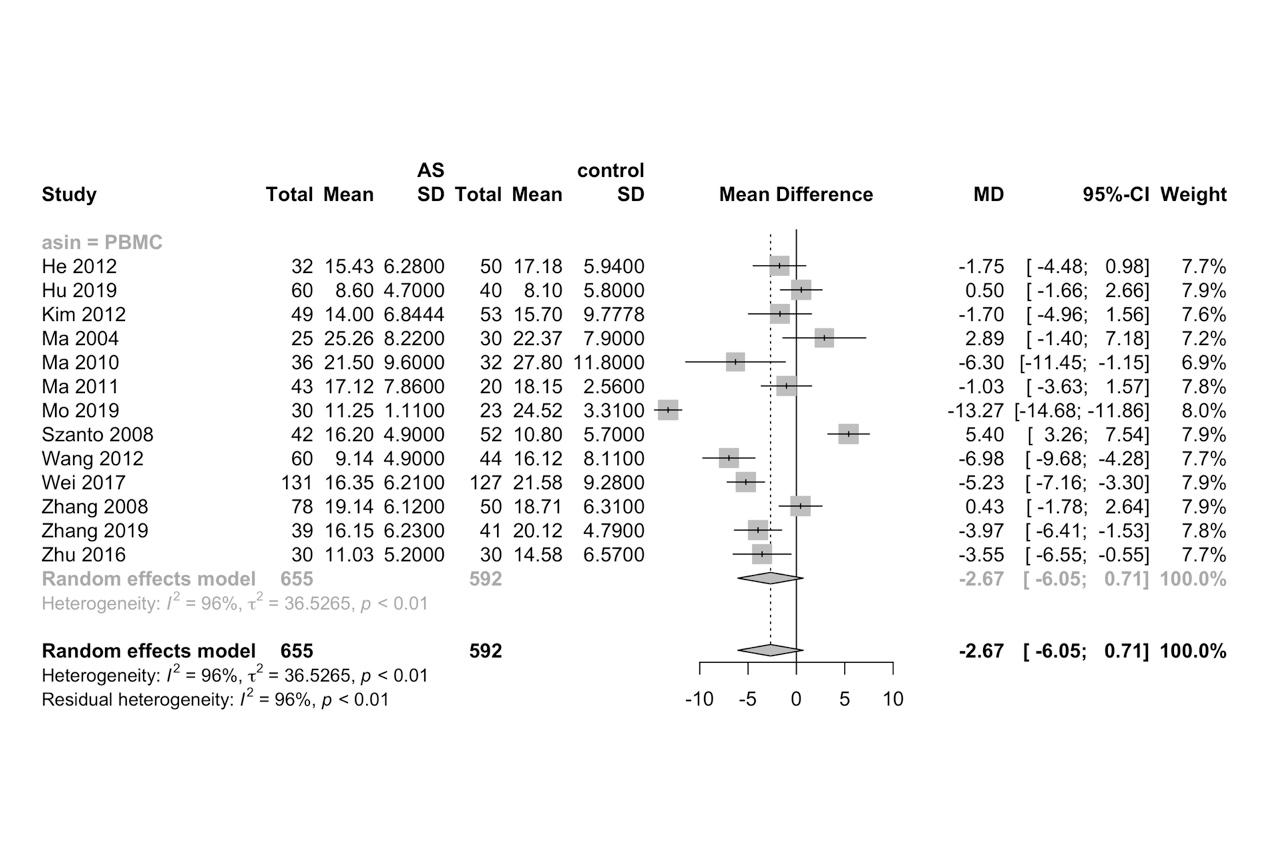


Figure 33. Proportions of NK cells in peripheral blood in AS patients


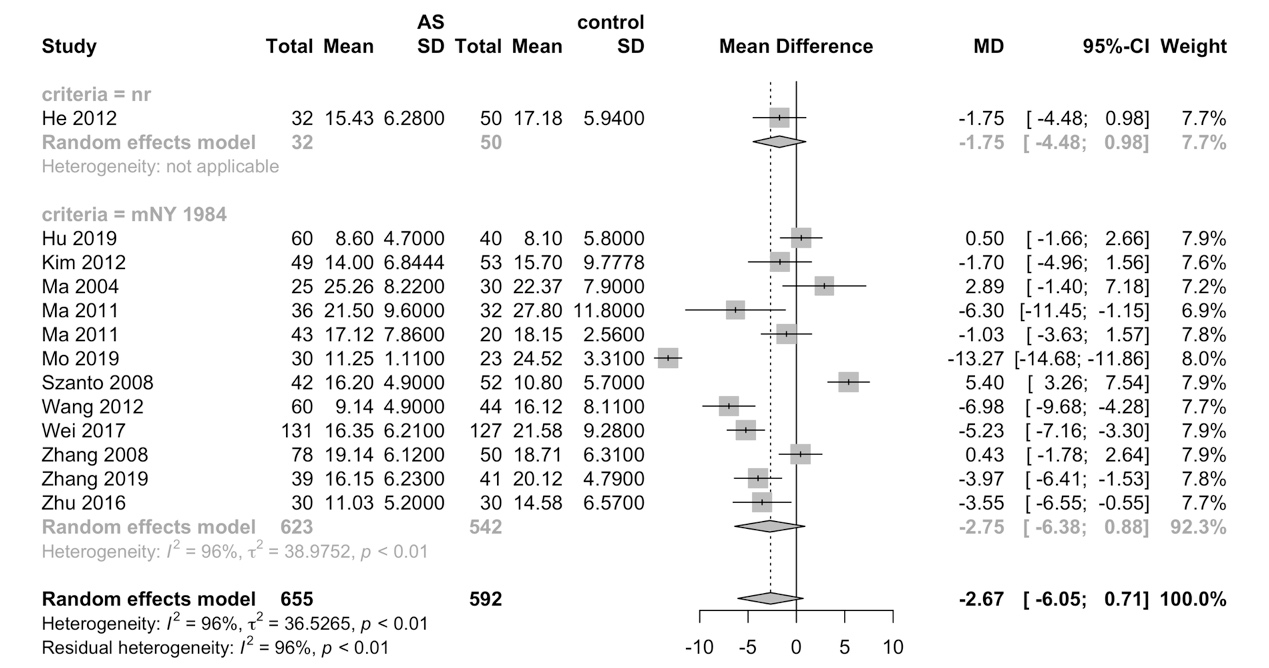


Figure 34. Subgroup analysis of proportions of NK cells in peripheral blood in AS patients by classification criteria


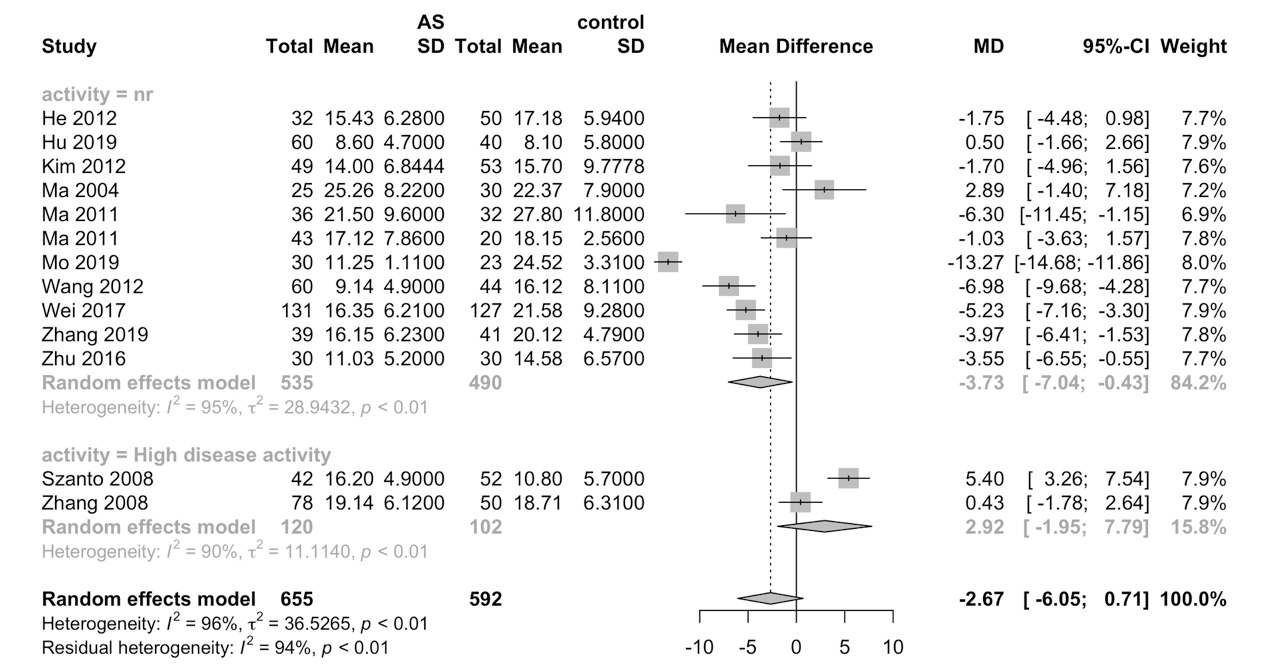


Figure 35. Subgroup analysis of proportions of NK cells in peripheral blood in AS patients by disease activity


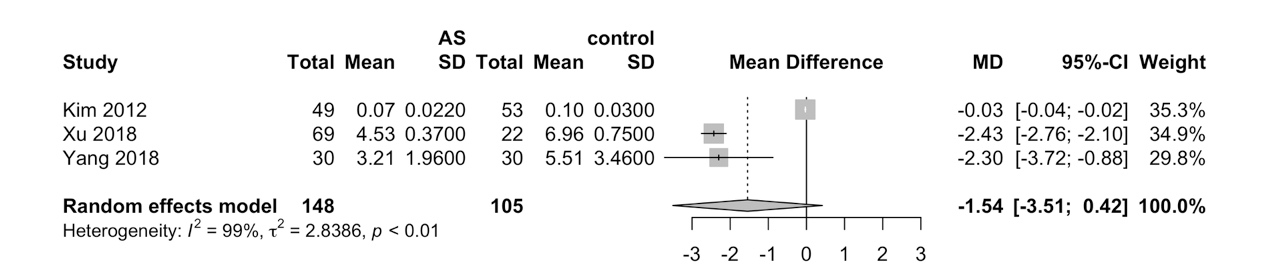


Figure 36. Proportions of NKT cells in peripheral blood in AS patients


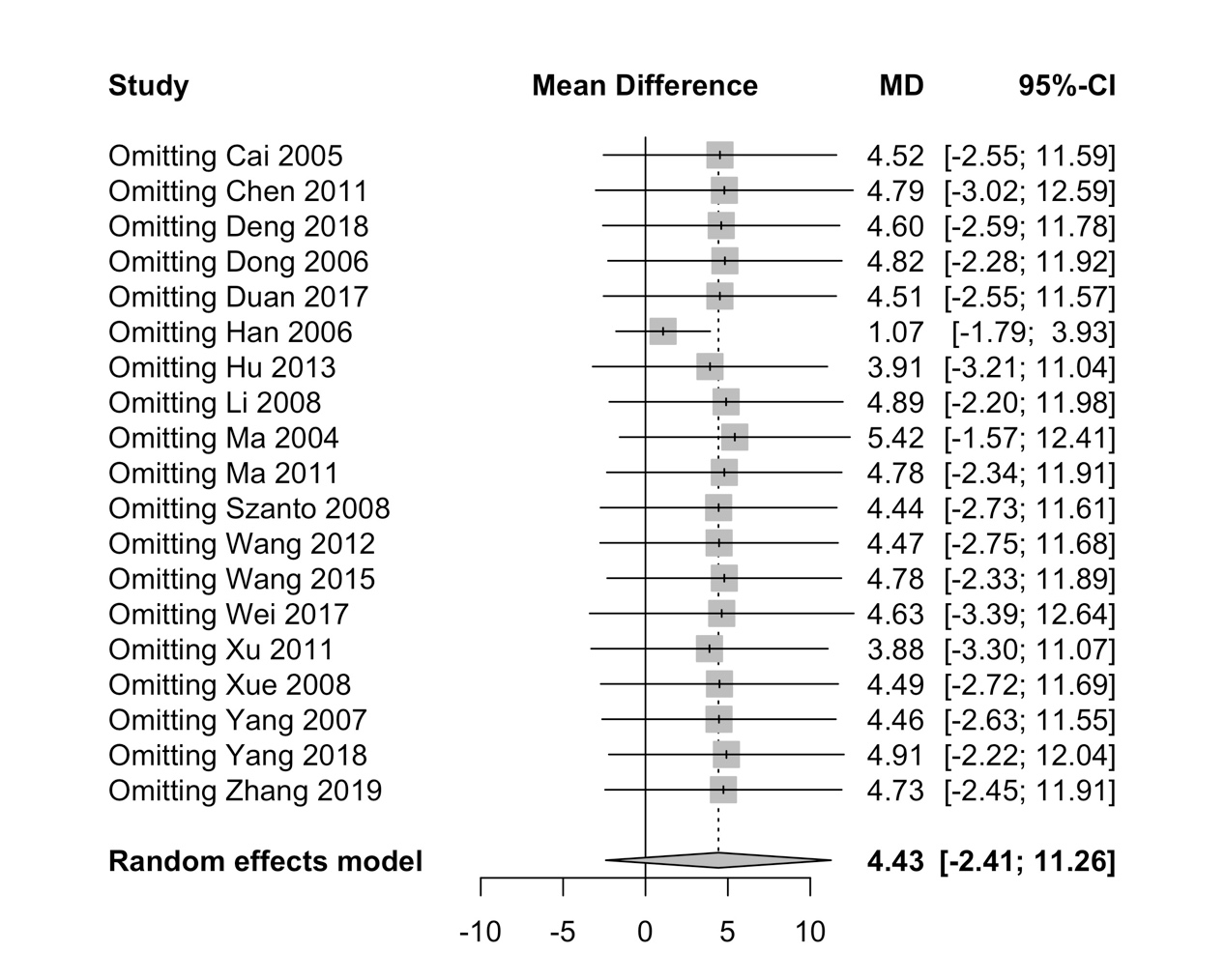
 Figure 37. Sensitivity analysis of T cells in peripheral blood in AS patients


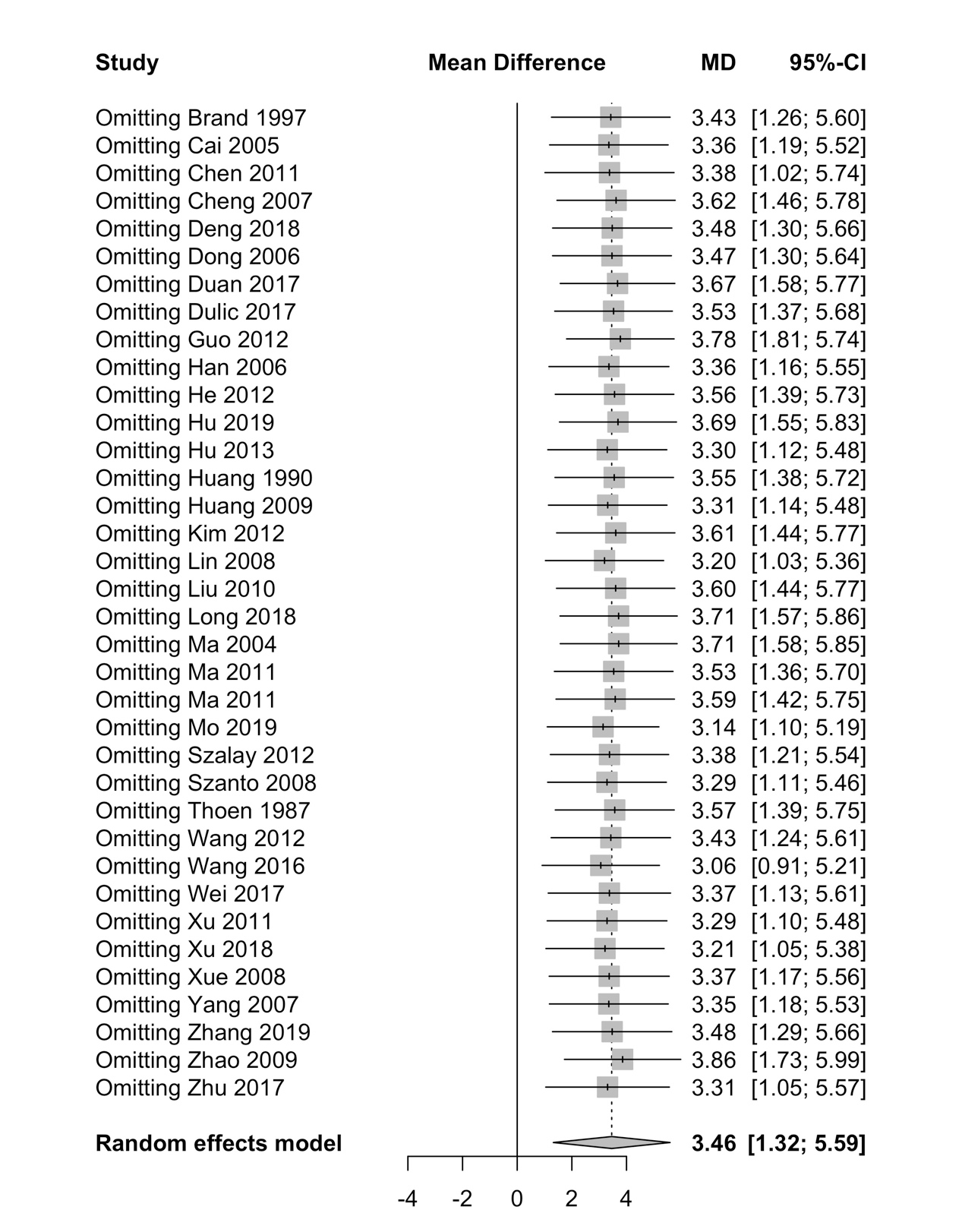


Figure 38. Sensitivity analysis of CD4+ T cells in peripheral blood in AS patients


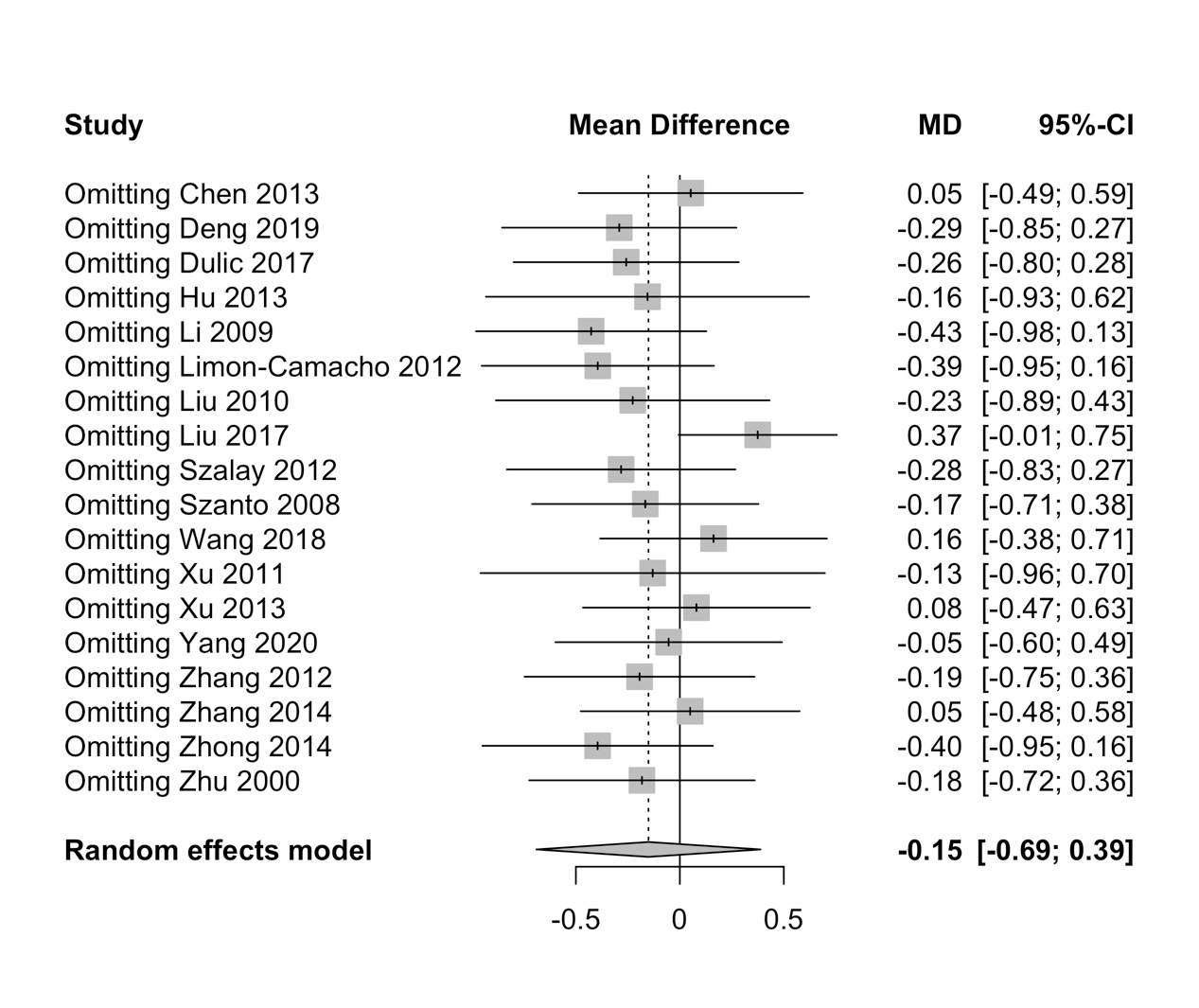


Figure 39. Sensitivity analysis of Th1 cells in peripheral blood in AS patients


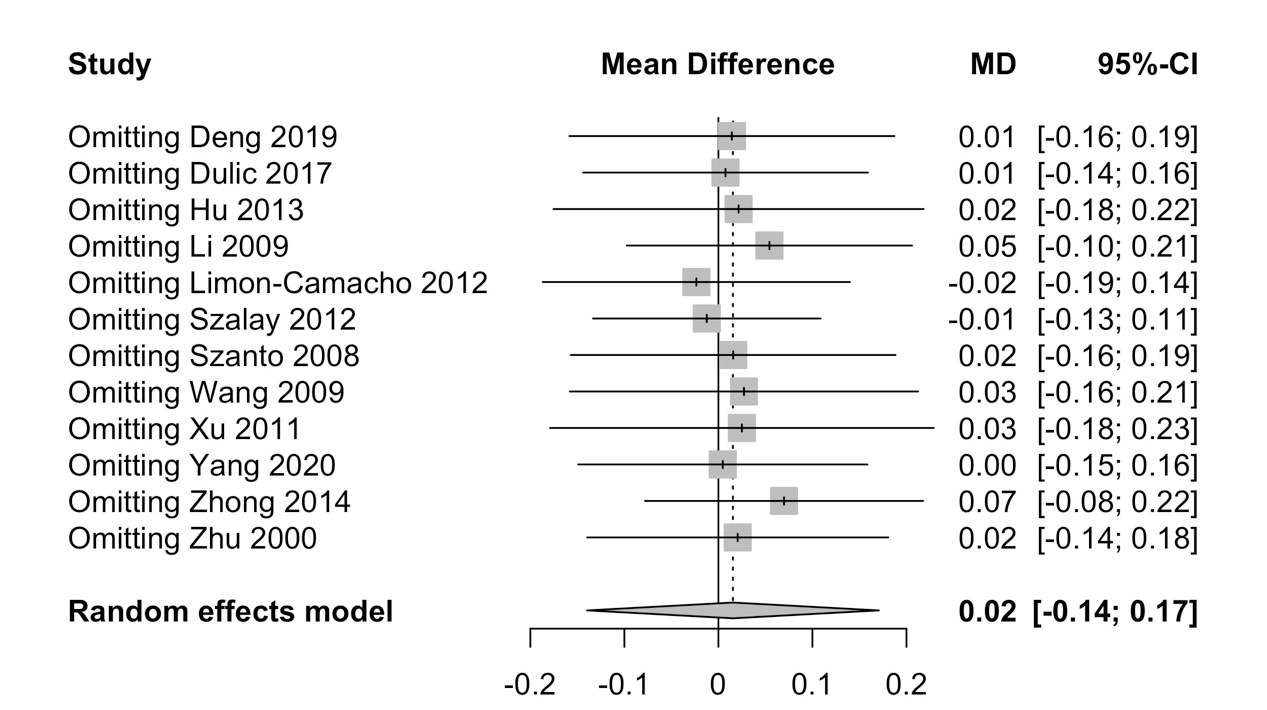


Figure 40. Sensitivity analysis of Th2 cells in peripheral blood in AS patients


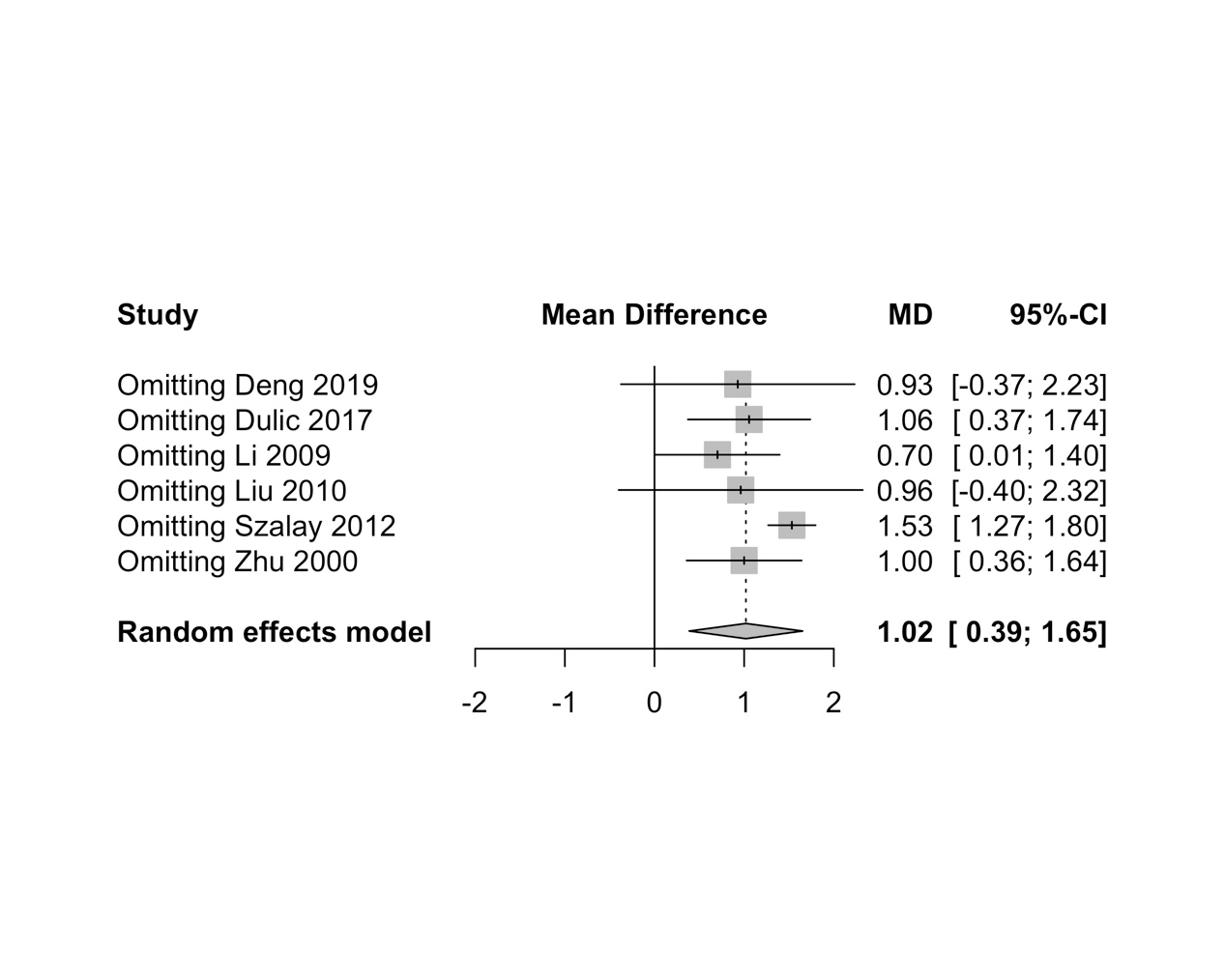


Figure 41. Sensitivity analysis of Th1/Th2 ratio in peripheral blood in AS patients


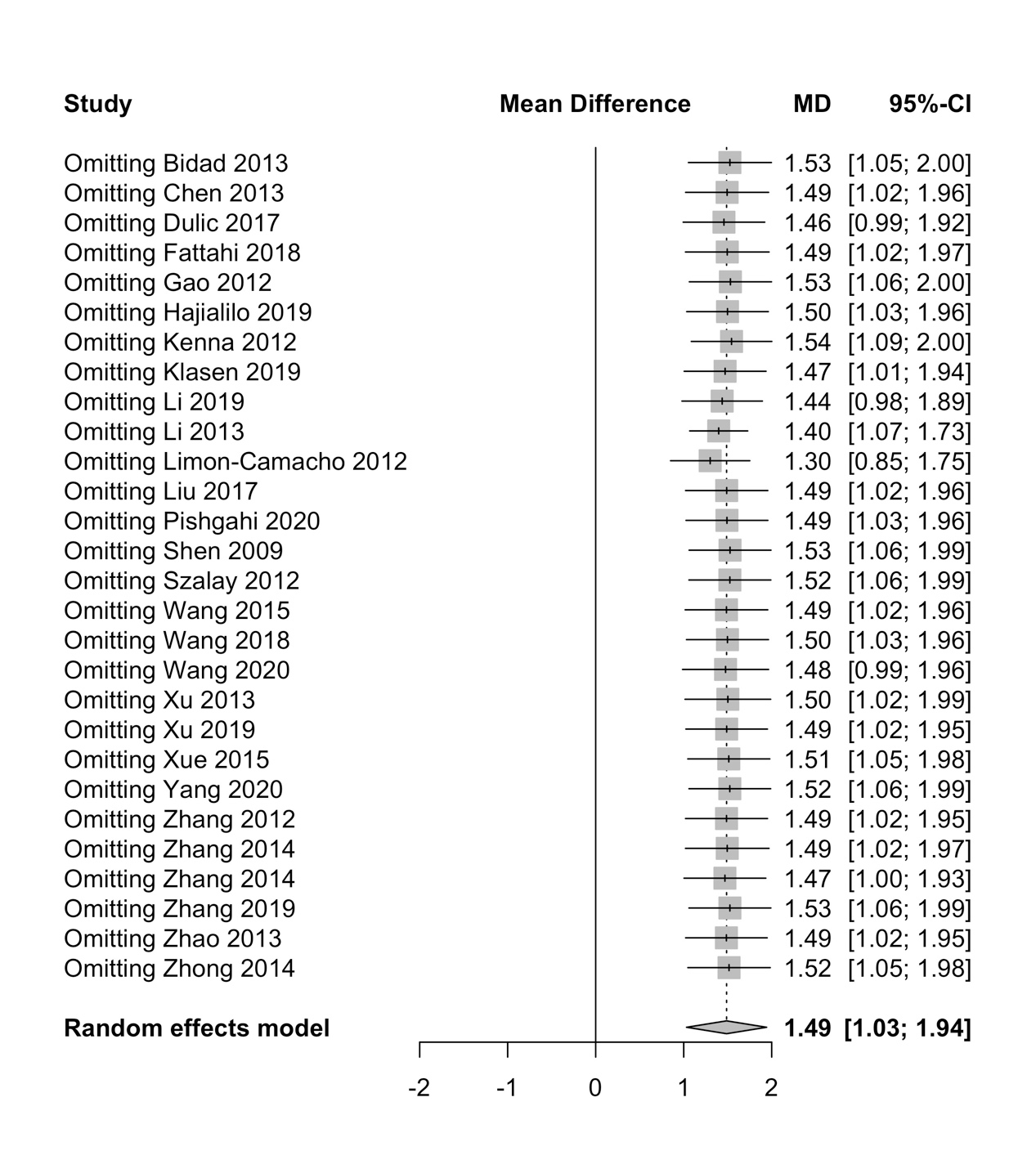


Figure 42. Sensitivity analysis of Th17 cells in peripheral blood in AS patients


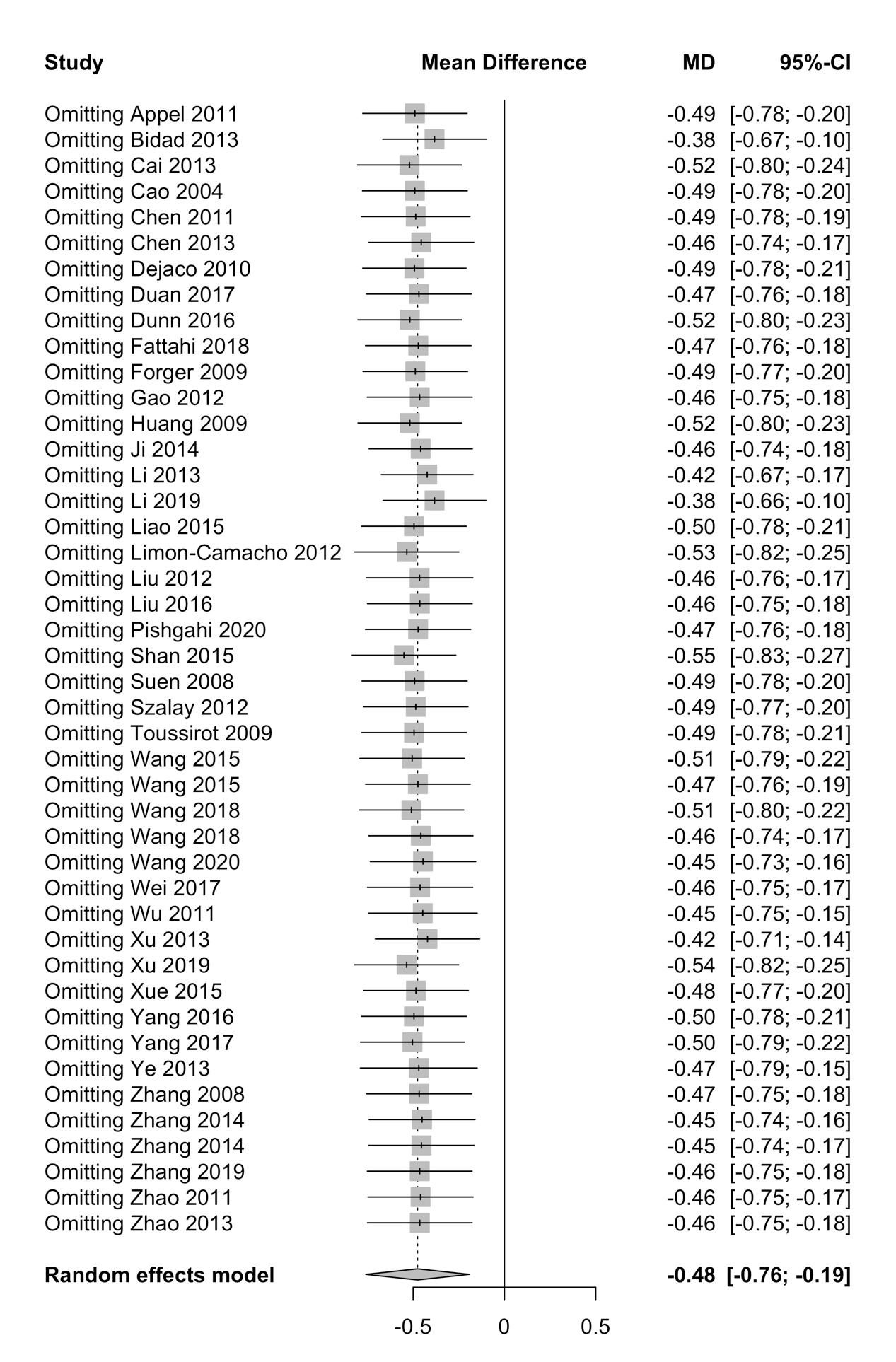


Figure 43. Sensitivity analysis of Tregs in peripheral blood in AS patients


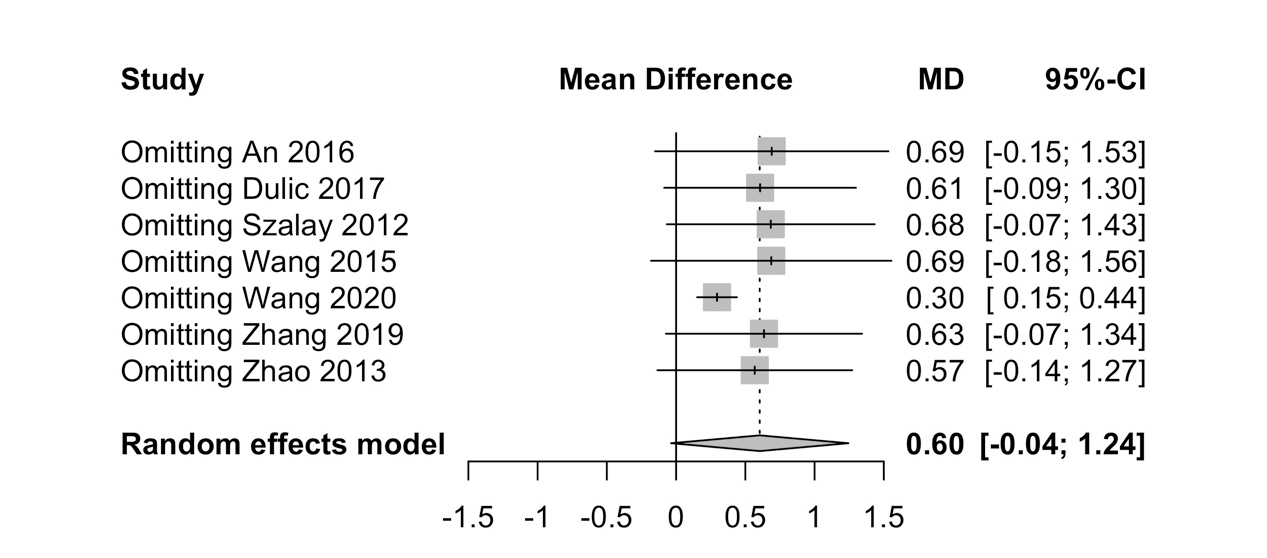


Figure 44. Sensitivity analysis of Th17/Tregs ratio in peripheral blood in AS patients


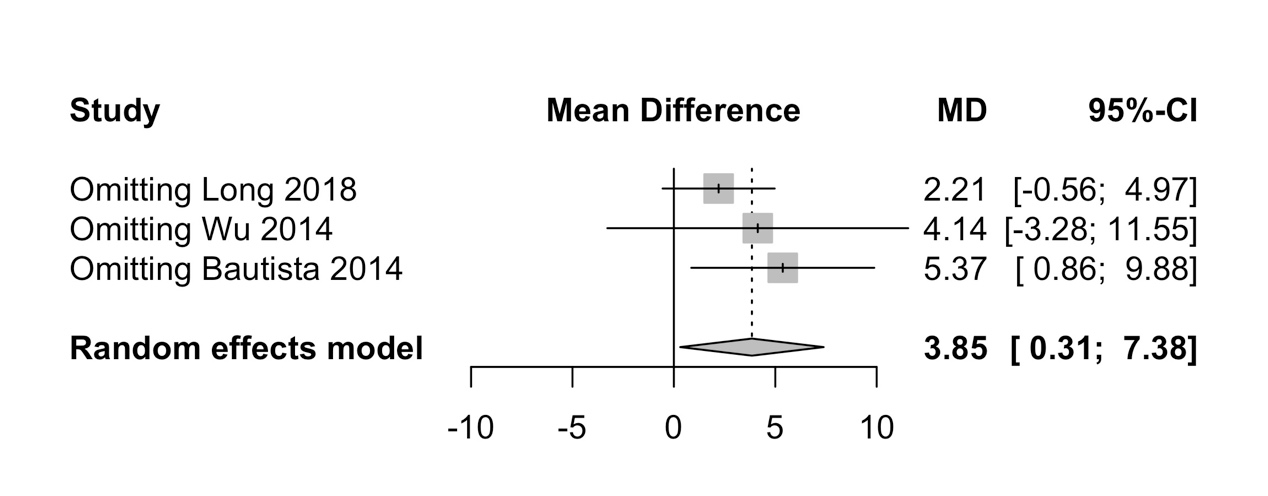


Figure 45. Sensitivity analysis of Tfh cells in peripheral blood in AS patients


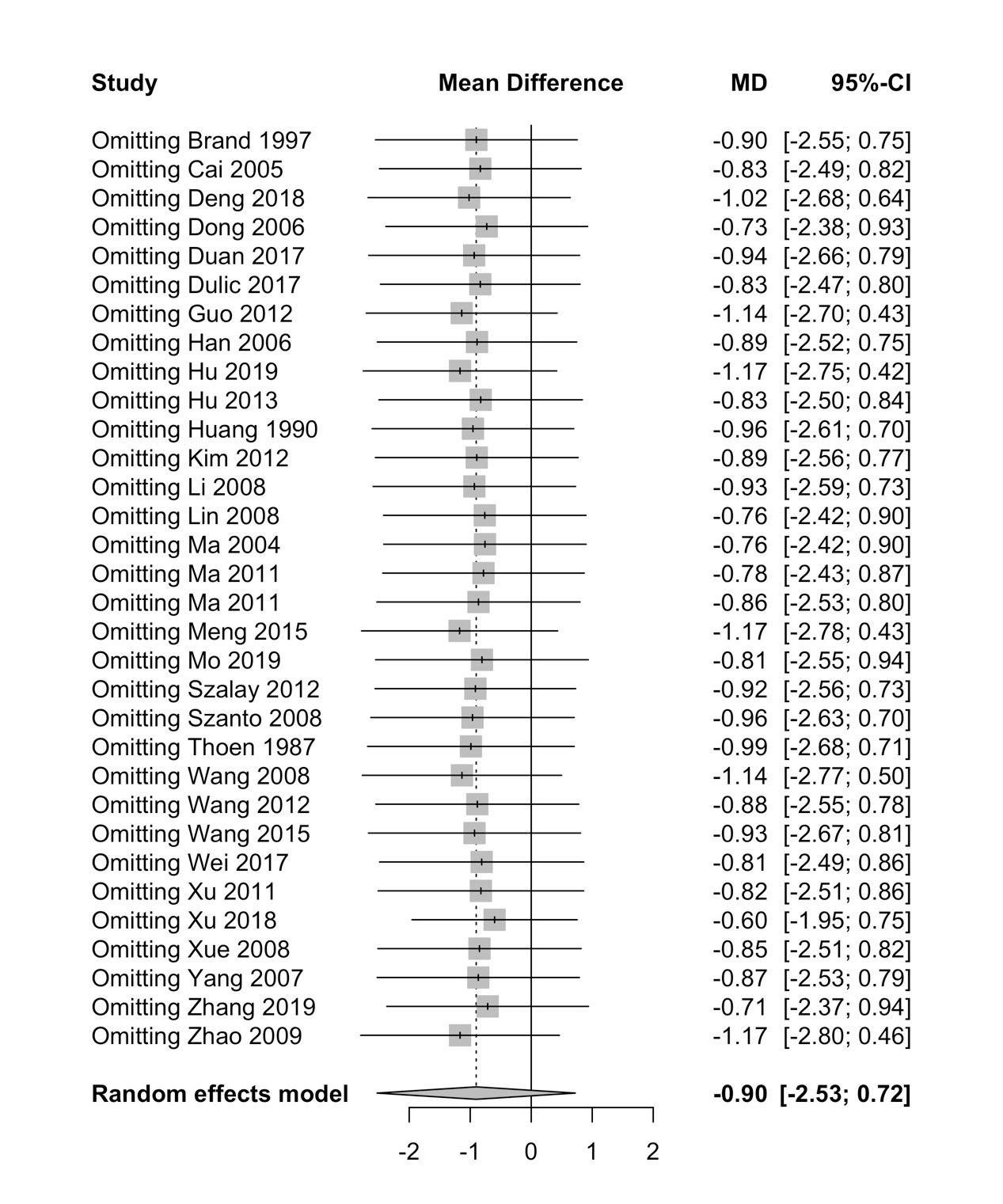


Figure 46. Sensitivity analysis of CD8+ T cells in peripheral blood in AS patients


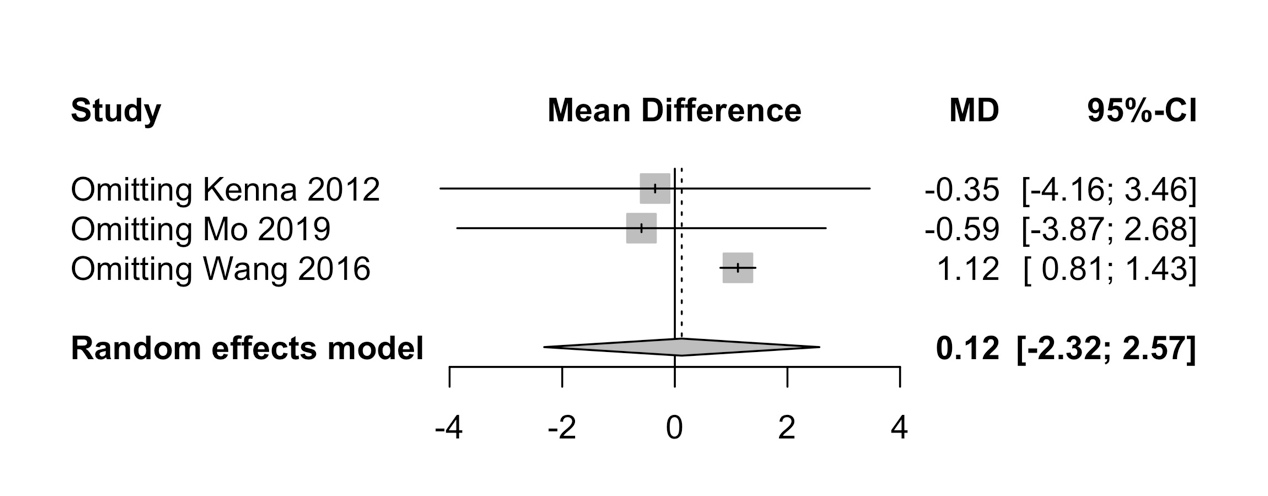


Figure 47. Sensitivity analysis of γδ T cells in peripheral blood in AS patients


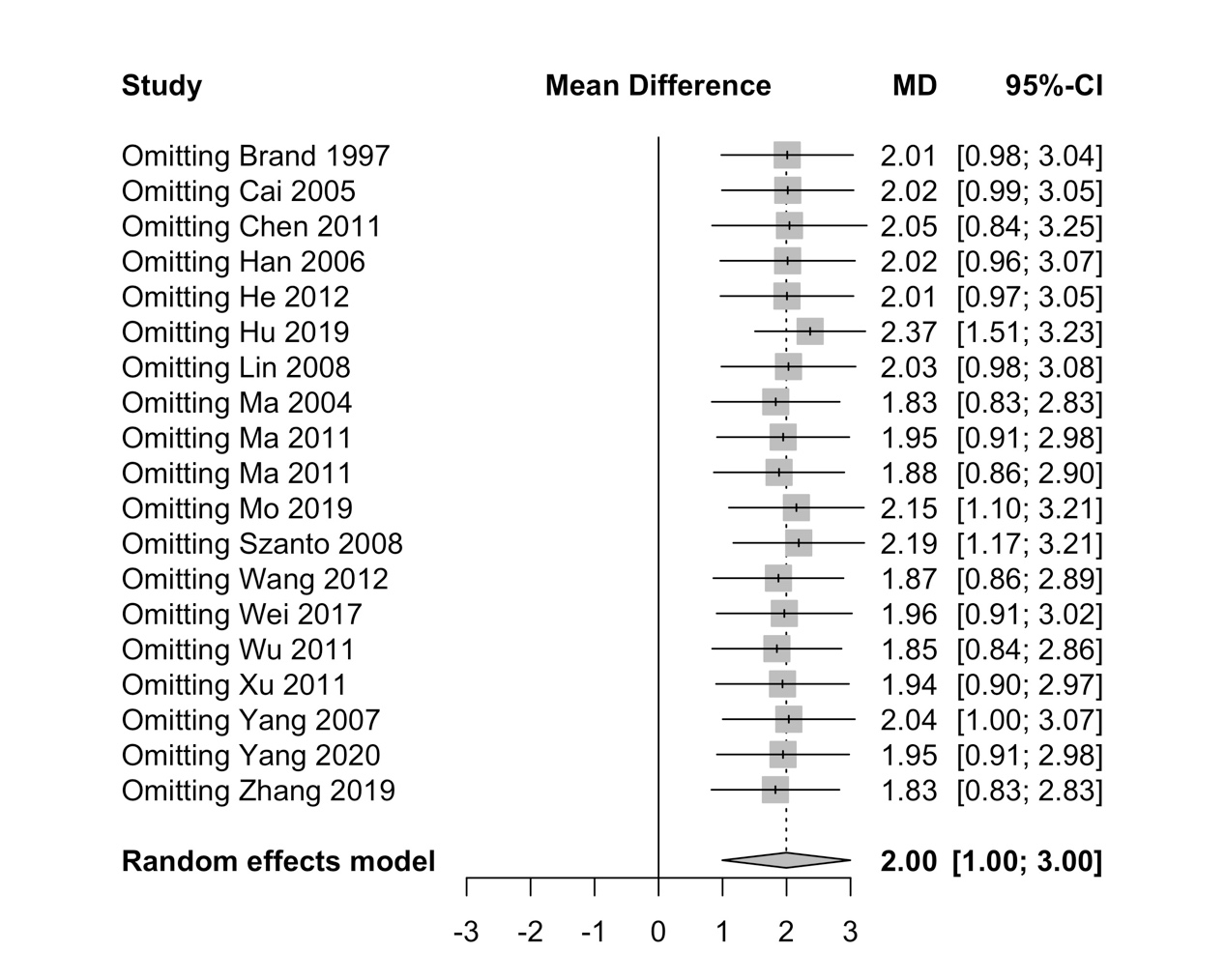


Figure 48. Sensitivity analysis of B cells in peripheral blood in AS patients


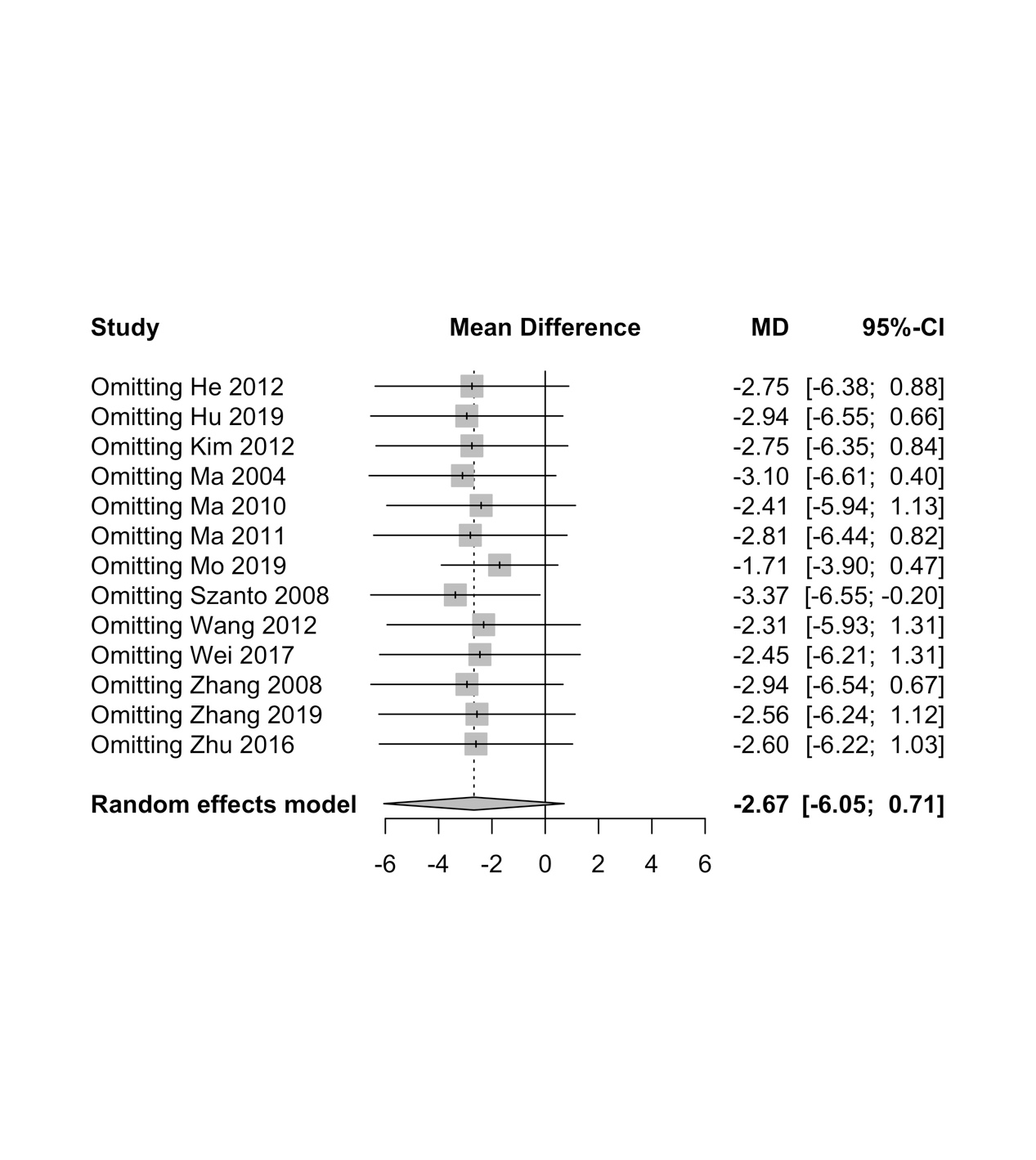


Figure 49. Sensitivity analysis of NK cells in peripheral blood in AS patients


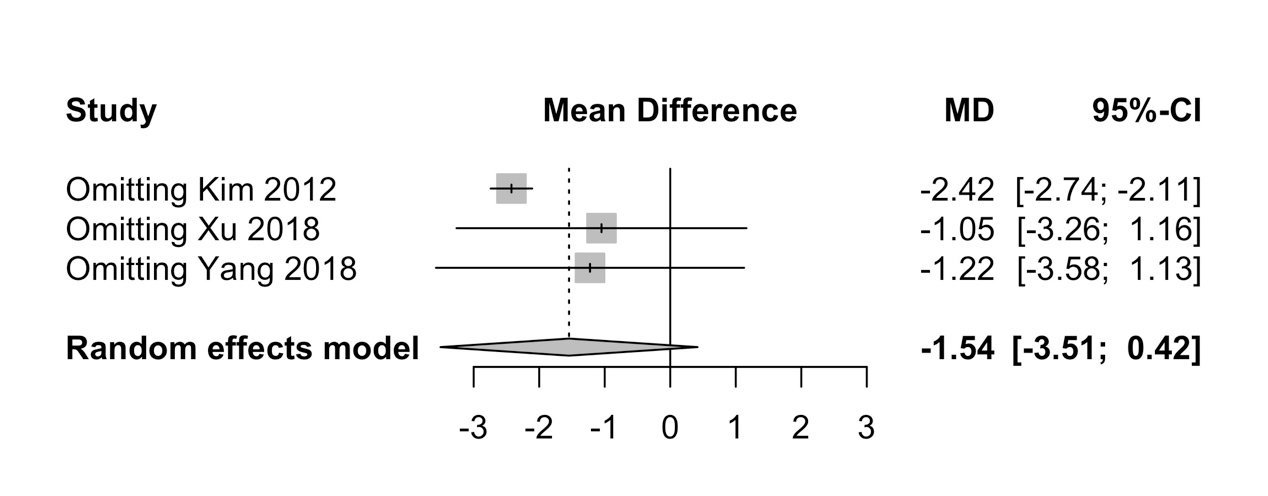


Figure 50. Sensitivity analysis of NKT cells in peripheral blood in AS patients
