# Supplementary material for: Imbalance of Peripheral Lymphocyte Subsets in Patients With Ankylosing Spondylitis: A Meta-Analysis
Source: Front Immunol. 2021 Jul 6;12:696973. doi: 10.3389/fimmu.2021.696973 (PMC8291033; doi:10.3389/fimmu.2021.696973)
Supplement: Supplementary file 2 [file Table_1.docx]

| Table 1. Characteristics of 95 individual studies included in the meta-analysis | | | | | | |
| --- | --- | --- | --- | --- | --- | --- |
| Author (ref.) | Publish year | Case numbers (AS/HC) | Lymphocyte subset | Lymphocyte's definition | Lymphocyte Proportions in AS | Lymphocyte Proportions in HC |
| An | 2016 | 73/85 | Th17/Treg ratio | / | 0.34±0.29 | 0.25±0.15 |
| Appel | 2011 | 19/20 | Treg in CD4+T | CD4+FoxP3+ | 5.55±2.45 | 5.18±1.99 |
| Bautista | 2014 | 25/50 | Tfh in CD4+T | CD4+CXCR5+ | 18.6±7.6 | 18.3±5.8 |
| Bidad | 2013 | 18/18 | Th17 in CD4+T | IL-17+ IFNγ- | 1.2±0.1 | 0.7±0.07 |
|  |  |  | Treg | CD4+FOXP3+RORγt-Tbet- | 9.7±1.2 | 16.1±3 |
| Brand | 1997 | 21/29 | B | CD19+ | 11.7±5.6 | 10±3.3 |
|  |  |  | CD4+T | CD4+ | 49.7±9.2 | 45±7.3 |
|  |  |  | CD8+T | CD8+ | 23.5±7.7 | 24.5±7.5 |
| Cai | 2013 | 40/20 | Treg in CD4+T | CD4+CD25hi | 11.41±3.69 | 8.78±2.84 |
| Cai | 2005 | 30/20 | B | CD19+ | 11.9±5.26 | 10.4±4.64 |
|  |  |  | CD4+T | CD3+CD4+ | 44.9±9.6 | 37.5±8.9 |
|  |  |  | CD8+T | CD3+CD8+ | 25.7±8.6 | 28.9±6.8 |
|  |  |  | T | CD3+ | 72.6±12.2 | 69.9±8.1 |
| Cao | 2004 | 10/29 | Treg | CD4+CD25bright | 1.31±0.68 | 1.23±0.64 |
| Chen | 2013 | 61/36 | Th1 in CD4+T | CD4+/IFNγ+ | 3.98±0.72 | 6.85±1.36 |
|  |  |  | Th17 in CD4+T | CD4+IL-17+ | 1.987±0.58 | 0.647±0.33 |
|  |  |  | Treg in CD4+T | CD4+CD25+FoxP3+ | 0.94±0.38 | 2.13±0.75 |
| Chen | 2011 | 23/25 | B | CD19+ | 8.5±1.01 | 7±0.45 |
|  |  |  | CD4+T | CD4+ | 37.53±1.65 | 31.55±0.92 |
|  |  |  | T | CD3+ | 64.66±2.3 | 66.69±1.91 |
|  |  |  | Treg | CD4+CD25HI | 2.18±0.11 | 2.16±0.1 |
| Cheng | 2007 | 25/21 | CD4+T | CD3+CD4+ | 34±8.3 | 36.4±7.2 |
| Dejaco | 2010 | 22/17 | Treg | CD4+CD25hi | 13.54±16.55 | 3.08±2.48 |
| Deng | 2019 | 49/100 | Th1 | CD4+/IFNγ+ | 2.29±1.21 | 0.68±0.24 |
|  |  |  | Th1/Th2 ratio | / | 1.97±0.53 | 0.49±0.24 |
|  |  |  | Th2 | CD4+/IL-4+ | 1.16±0.57 | 1.12±0.58 |
| Deng | 2018 | 91/50 | CD4+T | CD3+CD4+CD8- | 35.95±7.81 | 33.16±7.75 |
|  |  |  | CD8+T | CD3+CD4-CD8+ | 26.02±6.98 | 23.6±5.74 |
|  |  |  | T | CD3+ | 68.33±9.39 | 66.96±8.57 |
| Dong | 2006 | 30/30 | CD4+T | CD4+ | 43.4±9.9 | 40.3±4.1 |
|  |  |  | CD8+T | CD8+ | 20.2±6.7 | 26.2±2.9 |
|  |  |  | T | CD3+ | 58.8±10.4 | 61.5±5.2 |
| Duan | 2017 | 21/16 | CD4+T | CD4+ | 40.33±2.53 | 43.76±1.72 |
|  |  |  | CD8+T | CD8+ | 25.18±1.37 | 25.29±1.59 |
|  |  |  | T | CD3+ | 63.21±7,46 | 60.42±10.52 |
|  |  |  | Treg | CD4+CD25+CD127lo | 2.7±0.8 | 3.47±0.83 |
| Dulic | 2017 | 22/10 | Th17 in CD4+T | CD4+CCR4+CCR6+ | 3.33±1.39 | 1±0.17 |
|  |  |  | Treg in CD4+T | CD4+CD25+CD127- | 5.708±2.05 | 5.715±0.79 |
|  |  | 7/10 | CD4+T in T cells | CD4+ | 39.39±16.02 | 40.56±7.835 |
|  |  |  | CD8+T in T cells | CD8+ | 16.59±13.73 | 22.56±8.073 |
|  |  |  | Th1 in CD4+T | CD4+/IFNγ+ | 18.07±6.07 | 9.412±0.8024 |
|  |  |  | Th1/Th2 ratio | / | 2.435±1.526 | 1.656±0.437 |
|  |  |  | Th17/Treg ratio | / | 0.765±0.599 | 0.178±0.036 |
|  |  |  | Th2 in CD4+T | CD4+/IL-4+ | 9.766±4.653 | 5.937±1.155 |
| Fattahi | 2018 | 30/15 | Th17 | nr | 2.4±0.21 | 1.1±0.26 |
|  |  |  | Treg | CD4+CD25+FoxP3+ | 2.7±0.23 | 3.3±0.47 |
| Forger | 2009 | 15/18 | Treg | CD4+CD25hi | 2.22±1.47 | 2.12±1.42 |
| Gao | 2012 | 40/37 | Th17 in T cells | CD3+CD8-IL-17+ | 1.02±0.43 | 0.68±0.29 |
|  |  |  | Treg in CD4+T | CD4+CD25+FoxP3+ | 3.77±0.81 | 4.69±1.23 |
| Guo | 2012 | 98/76 | CD4+T | CD4+ | 31.87±3.19 | 38.45±5.26 |
|  |  |  | CD8+T | CD8+ | 28.02±4.23 | 22.3±4.65 |
| Hajialilo | 2019 | 24/35 | Th17 | CD4+IL-17+ | 4.51±1.3 | 3.32±1.32 |
| Han | 2006 | 69/50 | B | CD19+ | 12.5±3.89 | 10.8±3.47 |
|  |  |  | CD4+T | CD3+CD4+ | 47.1±6.43 | 40.3±5.33 |
|  |  |  | CD8+T | CD3+CD8+ | 27.5±4.75 | 29.8±50.8 |
|  |  |  | T | CD3+ | 75.4±8.12 | 10.7±7.16 |
| He | 2012 | 32/50 | B | CD3-CD19+ | 11.84±4.7 | 10.01±3.21 |
|  |  |  | CD4+T | CD3+CD4+ | 35.69±6.92 | 35.81±7.53 |
|  |  |  | NK | CD3-CD16+CD56+ | 15.43±6.28 | 17.18±5.94 |
| Hu | 2019 | 60/40 | B | CD19+ | 7.8±2.4 | 12.3±3.5 |
|  |  |  | CD4+T | CD3+CD4+ | 28.5±5.7 | 32.7±6.1 |
|  |  |  | CD8+T | CD3+CD8+ | 31.8±4.6 | 25.2±4.2 |
|  |  |  | NK | CD16+CD56+ | 8.6±4.7 | 8.1±5.8 |
| Hu | 2013 | 32/30 | CD4+T | CD3+CD4+ | 48.09±6.27 | 39.15±5.42 |
|  |  |  | CD8+T | CD3+CD8+ | 24.68±4.63 | 27.74±4.58 |
|  |  |  | T | CD3+ | 75.31±6.13 | 61.72±5.98 |
|  |  |  | Th1 | CD3+CD4+INFγ+ | 1.87±0.13 | 0.45±0.06 |
|  |  |  | Th2 | CD3+CD4+IL-4+ | 1.12±0.19 | 1.07±0.18 |
| Huang | 2009 | 20/9 | CD4+T | CD3+CD4+ | 44.25±8.63 | 35.38±4.29 |
|  |  |  | Treg in CD4+T | CD4+CD25+CD127lo/- | 12.67±5.26 | 8.31±1.63 |
| Huang | 1990 | 9/9 | CD4+T | CD4+ | 33.9±6.3 | 33.8±2.1 |
|  |  |  | CD8+T | CD8+ | 29±6 | 28.1±2.7 |
| Ji | 2014 | 20/20 | Treg | CD4+CD25+CD127lo | 40.1±17.5 | 58.6±10.2 |
| Kenna | 2012 | 17/20 | Th17 | CD4+IL-17+ | 0.41±0.22 | 0.42±0.1 |
|  |  |  | γδT | CD3+TCRγ+ | 1.53±0.34 | 0.46±0.17 |
| Kim | 2012 | 49/53 | CD4+T | CD3+CD4+ | 35.5±9.11 | 37.3±10.81 |
|  |  |  | CD8+T | CD3+CD8+ | 22.3±6.96 | 23.5±7.93 |
|  |  |  | NK | CD3-CD56+ | 14±6.84 | 15.7±9.78 |
|  |  |  | NKT | CD3+6B11+ | 0.07±0.022 | 0.1±0.03 |
| Klasen | 2019 | 14/5 | Th17 | CD4+CD45RO+RA−IL-17+ | 2.34±0.56 | 0.4±0.2 |
| Li | 2019 | 64/60 | Th17 | CD4+IL-17A+ | 5.37±0.28 | 2.56±0.41 |
|  |  |  | Treg | CD4+CD25+CD127lo | 5.11±0.49 | 9.26±2.03 |
| Li | 2013 | 222/68 | Th17 in CD4+T | CD4+IL-17+ | 3.84±0.62 | 0.24±0.05 |
|  |  |  | Treg in CD4+T | CD4+CD25+FoxP3+ | 2.14±0.44 | 4.99±0.49 |
| Li | 2009 | 30/10 | Th1 | CD3+CD8-INFγ+ | 6.85±2.55 | 2.6±0.95 |
|  |  |  | Th1/Th2 ratio | / | 3.3±1.33 | 0.97±0.43 |
|  |  |  | Th2 | CD3+CD8-IL-4+ | 2.16±0.66 | 2.92±0.79 |
| Li | 2008 | 50/21 | CD8+T | CD3+CD8+ | 32±6 | 32±6 |
|  |  |  | T | CD3+ | 65±9 | 69±8 |
| Liao | 2015 | 69/30 | Treg | CD4+CD25+FoxP3+ | 1.73±1.08 | 1.51±0.48 |
| Limon-Camacho | 2012 | 39/25 | Th1 in CD4+T | CD3+CD4+IFNγ+ | 4±1.3 | 1.1±0.3 |
|  |  |  | Th17 in CD4+T | CD3+CD4+IL-17A+ | 7.4±1.8 | 0.7±0.2 |
|  |  |  | Th2 in CD4+T | CD3+CD4+IL-4+ | 1.3±0.4 | 1±0.4 |
|  |  |  | Treg in CD4+T | CD3+CD4+FoxP3+ | 7.3±1.3 | 5.3±1.7 |
| Lin | 2009 | 66/30 | B | CD19+ | 9.61±4.71 | 8.16±2.61 |
| Lin | 2008 | 66/30 | CD4+T | CD3+CD4+ | 57±10 | 44±10 |
|  |  |  | CD8+T | CD3+CD8+ | 37±8 | 42±4 |
| Liu | 2017 | 38/38 | Th1 | CD4+/IFNγ+ | 4.75±0.68 | 11.85±0.96 |
|  |  |  | Th17 | CD4+IL-17+ | 2.38±0.78 | 0.92±0.24 |
| Liu | 2016 | 60/20 | Treg | CD4+CD25+CD127- | 5.68±1.36 | 6.71±1.75 |
| Liu | 2012 | 60/30 | Treg | CD4+CD25+CD127- | 1.51±0.26 | 2.3±0.38 |
| Liu | 2010 | 30/20 | Th1/Th2 ratio | / | 1.81±0.3 | 0.447±0.09 |
| Long | 2018 | 65/20 | CD4+T | CD3+CD4+ | 30.73±8.01 | 35.91±5.35 |
|  |  |  | Tfh in CD4+T | CD4+CXCR5+ | 23.45±8.82 | 15.58±4.49 |
| Ma | 2011 | 43/20 | B | CD19+ | 13.48±5.34 | 9.4±2.3 |
|  |  |  | CD4+T | CD3+CD4+ | 37.58±8.35 | 38.65±6.33 |
|  |  |  | CD8+T | CD3+CD8+ | 27.98±7.55 | 30±2.72 |
|  |  |  | NK | CD16+CD56+ | 17.12±7.86 | 18.15±2.56 |
|  |  |  | T | CD3+ | 67.51±9.52 | 69.5±4.93 |
| Ma | 2011 | 36/32 | B | CD3-CD19+ | 11.8±4.3 | 8.9±3.1 |
|  |  |  | CD4+T | CD3+CD4+ | 36.4±5.5 | 35.5±8.8 |
|  |  |  | CD8+T | CD3+CD8+ | 39±9 | 43.9±8.4 |
|  |  |  | NK | CD3-CD16+CD56+ | 21.5±9.6 | 27.8±11.8 |
| Ma | 2004 | 25/30 | B | CD3-CD19+ | 14.25±2.98 | 9.42±3.04 |
|  |  |  | CD4+T | CD3+CD4+ | 29.46±3.25 | 34.6±6.56 |
|  |  |  | CD8+T | CD3+CD8+ | 22.78±3.88 | 27.89±5.4 |
|  |  |  | NK | CD3-CD16+CD56+ | 25.26±8.22 | 22.37±7.9 |
|  |  |  | T | CD3+ | 53.56±6.31 | 66.93±4.54 |
| Meng | 2015 | 42/20 | CD8+T | CD3+CD8+ | 21.22±4.27 | 14.23±4.15 |
| Mo | 2019 | 30/23 | B | CD19+ | 9.94±0.94 | 10.33±0.79 |
|  |  |  | CD4+T | CD3+CD4+ | 44.44±1.94 | 30.76±1.62 |
|  |  |  | CD8+T | CD3+CD8+ | 24.75±1.21 | 28.39±1.73 |
|  |  |  | NK | CD16+/CD56+ | 11.25±1.11 | 24.52±3.31 |
|  |  |  | γδT | CD3+TCRγ+ | 11.8±1.97 | 10.18±1.7 |
| Pishgahi | 2020 | 31/35 | Th17 | CD4+IL-17+ | 4.68±0.91 | 3.3±0.89 |
|  |  |  | Treg | CD4+CD25+CD127- | 3.44±0.72 | 4.02±0.76 |
| Shan | 2015 | 20/10 | Treg | CD4+FOXP3+CXCR5+ | 5.57±1.28 | 3.08±0.59 |
| Shen | 2009 | 20/16 | Th17 | CD4+IL-17+ | 0.94±0.49 | 0.5±0.34 |
| Suen | 2008 | 23/26 | Treg in CD4+T | CD4+CD25hiFoxP3+ | 0.97±0.33 | 0.86±0.39 |
| Szalay | 2012 | 13/9 | CD4+T | CD4+ | 42.7±7.63 | 35.8±8.533 |
|  |  |  | CD8+T | CD8+ | 17.7±5.037 | 18±10.1 |
|  |  |  | Th1 | CD4+CXCR3+ | 12.9±1.519 | 9.81±2.652 |
|  |  |  | Th1/Th2 ratio | / | 1.31±0.585 | 2.38±0.511 |
|  |  |  | Th17 | CD4+CCR4+CCR6+ | 1.18±0.496 | 0.69±0.126 |
|  |  |  | Th17/Treg ratio | / | 0.27±0.193 | 0.14±0.059 |
|  |  |  | Th2 | CD4+CCR4+ | 9.18±3.059 | 4.54±0.481 |
|  |  |  | Treg | CD4+CD25+CD127- | 4.45±1.437 | 4.42±1.348 |
| Szanto | 2008 | 42/52 | B | CD19+ | 10.2±4.2 | 11.7±4.1 |
|  |  |  | CD4+T | CD4+ | 54.8±9.9 | 45.3±7.7 |
|  |  |  | CD8+T | CD8+ | 20.4±4.5 | 19.6±5.9 |
|  |  |  | NK | CD56+ | 16.2±4.9 | 10.8±5.7 |
|  |  |  | T | CD3+ | 74.7±8.3 | 70.5±7.3 |
|  |  |  | Th1 | CD4+/IFNγ+ | 25.5±8.2 | 24.9±7.9 |
|  |  |  | Th2 | CD4+/IL-4+ | 0.48±0.55 | 0.45±0.39 |
| Thoen | 1987 | 31/15 | CD4+T in T cells | CD3+CD4+ | 41.7±2 | 41.9±2.8 |
|  |  |  | CD8+T in T cells | CD3+CD8+ | 27.2±1.5 | 25.8±1.6 |
| Toussirot | 2009 | 32/15 | Treg in CD4+T | CD4+CD25+FoxP3+ | 8.2±0.61 | 7.94±1.04 |
| Wang | 2020 | 90/90 | Th17 | nr | 2.73±0.35 | 0.91±0.29 |
|  |  |  | Th17/Treg ratio | / | 2.65±0.14 | 0.58±0.36 |
|  |  |  | Treg | nr | 1.19±0.41 | 2.7±0.17 |
| Wang | 2018 | 30/30 | Th1 | CD4+/IFNγ+ | 11.69±1.53 | 17.27±3.01 |
|  |  |  | Th17 | CD4+IL-17+ | 2.05±0.88 | 0.83±0.29 |
|  |  |  | Treg | CD4+CD25+CD127lo/- | 6.84±2.59 | 8.29±2.25 |
| Wang | 2018 | 26/26 | Treg in CD4+T | CD4+CD25+FoxP3+CD127- | 6.32±1.5 | 5.44±1.02 |
| Wang | 2016 | 50/50 | CD4+T | CD4+ | 49.8±11.63 | 32.1±8.04 |
|  |  |  | γδT | nr | 2.54±1.12 | 4.81±1.33 |
| Wang | 2015 | 78/30 | Th17/Treg ratio | / | 0.22±0.13 | 0.11±0.05 |
|  |  |  | Treg | CD4+CD25+CD127lo | 7.59±1.97 | 8.16±2.16 |
| Wang | 2015 | 45/20 | Th17 | CD4+IL-17+ | 2.07±0.63 | 0.54±0.23 |
|  |  |  | Treg | CD4+CD25+FoxP3+ | 1.81±0.81 | 1.23±0.52 |
|  |  | 22/20 | CD8+T | CD3+CD8+ | 22.03±1.69 | 22.31±1.36 |
|  |  |  | T | CD3+ | 59.61±8.2 | 61.6±4.61 |
| Wang | 2012 | 60/44 | B | CD19+ | 15.07±6.53 | 10.79±3.35 |
|  |  |  | CD4+T | CD3+CD4+ | 38.33±7.21 | 33.79±6.65 |
|  |  |  | CD8+T | CD3+CD8+ | 24.07±8.52 | 25.54±5.89 |
|  |  |  | NK | CD16+CD56+ | 9.14±4.9 | 16.12±8.11 |
|  |  |  | T | CD3+ | 65.83±8.5 | 62.13±6.04 |
| Wang | 2008 | 30/20 | CD4+T | CD3+CD4+ | 33.65±5.62 | 35.1±4.29 |
|  |  |  | CD8+T | CD3+CD8+ | 23.74±6.81 | 17.55±3.89 |
|  |  |  | Th1 | CD3+CD4+INFγ+ | 1.98±0.17 | 0.48±0.07 |
|  |  |  | Th2 | CD3+CD4+IL-4+ | 1.09±0.2 | 1.11±0.21 |
| Wei | 2017 | 131/127 | B | CD19+ | 11.67±4.51 | 9.12±3.3 |
|  |  |  | CD4+T | CD3+CD4+ | 41.51±5.92 | 35.33±4.63 |
|  |  |  | CD8+T | CD3+CD8+ | 25.18±8.11 | 28.65±7.21 |
|  |  |  | NK | CD16+/CD56+ | 16.35±6.21 | 21.58±9.28 |
|  |  |  | T | CD3+ | 70.1±5.02 | 69.3±4.36 |
|  |  |  | Treg | CD4+CD25hi+CD127lo | 1.99±1.2 | 2.96±1.25 |
| Wu | 2014 | 60/60 | Tfh in CD4+T | CD4+CXCR5+ICOS+ | 0.27±0.2 | 0.21±0.14 |
|  |  |  | Tfh in CD4+T | CD4+CXCR5+ | 17.53±4.1 | 14.28±3.05 |
| Wu | 2011 | 51/49 | Treg | CD4+CD25+FoxP3+ | 1.23±0.13 | 2.56±0.16 |
| Wu | 2011 | 24/30 | B | CD19+ | 16.7±5.2 | 11.7±3.1 |
| Xu | 2019 | 18/9 | Th17 in CD4+T | CD4+IL-17A+ | 2.08±1.46 | 0.62±0.1 |
|  |  |  | Treg in CD4+T | CD4+CD25+ | 3.39±2.03 | 1.14±0.11 |
| Xu | 2018 | 69/22 | CD4+T | CD3+CD4+ | 59.26±1.44 | 47.88±1.93 |
|  |  |  | CD8+T | CD3+CD8+ | 31.74±0.99 | 39.94±1.72 |
|  |  |  | NKT | CD3+CD16+CD56+ | 4.53±0.37 | 6.96±0.75 |
| Xu | 2013 | 24/22 | Th1 | CD4+/IFNγ+ | 3.94±1.01 | 7.32±1.35 |
|  |  |  | Th17 | CD4+IL-17+ | 1.53±0.21 | 0.4±0.15 |
|  |  |  | Treg | CD4+CD25+CD127lo | 4.23±0.98 | 6.87±1.03 |
| Xu | 2011 | 78/50 | B | CD19+ | 14.2±3.61 | 11.2±3.56 |
|  |  |  | CD4+T | CD3+CD4+ | 47.3±6.24 | 38.2±5.13 |
|  |  |  | CD8+T | CD3+CD8+ | 24.5±4.54 | 27.6±4.26 |
|  |  |  | T | CD3+ | 76.4±6.24 | 62.3±6.32 |
|  |  |  | Th1 | CD3+CD4+INFγ+ | 1.85±0.15 | 0.43±0.06 |
|  |  |  | Th2 | CD3+CD4+IL-4+ | 1.13±0.22 | 1.08±0.21 |
| Xue | 2015 | 38/30 | Th17 | CD3+CD8-IL17+ | 1.79±0.79 | 0.97±0.46 |
|  |  |  | Treg | CD4+CD25hi | 2.66±1.01 | 2.8±1.22 |
| Xue | 2008 | 89/42 | CD4+T | CD3+CD4+ | 43.8±6.4 | 37.3±5.3 |
|  |  |  | CD8+T | CD3+CD8+ | 24.7±6.3 | 27.2±7.1 |
|  |  |  | T | CD3+ | 68.3±7.1 | 65±8.2 |
| Yang | 2020 | 67/50 | B | CD3-CD19+ | 12.86±5.53 | 9.92±4.36 |
|  |  |  | Th1 in CD4+T | CD3+CD4+CXCR5-CXCR3+CCR4- | 14.06±6.57 | 18.08±8.5 |
|  |  |  | Th17 in CD4+T | CD4+IL-17+ | 1.55±1.48 | 1.06±0.89 |
|  |  |  | Th2 in CD4+T | CD3+CD4+CXCR5-CXCR3-CCR4+ | 14.4±5.84 | 12.8±4.78 |
| Yang | 2018 | 30/30 | NKT | CD3+CD56+ | 3.21±1.96 | 5.51±3.46 |
|  |  |  | T | CD3+ | 62.88±5.99 | 67.14±6.7 |
| Yang | 2017 | 40/40 | Treg in CD4+T | CD4+CD25+ | 30.05±5.73 | 27.4±5.66 |
| Yang | 2016 | 38/31 | Treg in CD4+T | CD4+CD25+FoxP3+ | 3.39±0.81 | 3.15±0.87 |
| Yang | 2007 | 60/30 | B | CD19+ | 11.6±5.22 | 10.4±5.62 |
|  |  |  | CD4+T | CD3+CD4+ | 45.8±8.8 | 38.6±9 |
|  |  |  | CD8+T | CD3+CD8+ | 26.8±8.8 | 28.8±7.6 |
|  |  |  | T | CD3+ | 73.8±11.6 | 70±9.8 |
| Ye | 2013 | 21/22 | Treg | CD4+CD45RO+FoxP3hi | 0.48±0.07 | 0.73±0.07 |
| Zhang | 2019 | 60/30 | Th17 | CD4+IL-17+ | 1.07±0.96 | 0.59±0.36 |
|  |  |  | Th17/Treg ratio | / | 0.65±0.77 | 0.23±0.2 |
|  |  |  | Treg | CD4+CD25+FoxP3+ | 1.65±1.25 | 2.62±1.76 |
| Zhang | 2019 | 39/41 | B | CD3-CD19+ | 15.69±4.87 | 10.69±2.69 |
|  |  |  | CD4+T | CD3+CD4+ | 39.01±6.99 | 36.12±4.01 |
|  |  |  | CD8+T | CD3+CD8+ | 23.63±5.41 | 29.94±4.76 |
|  |  |  | NK | CD16+CD56+ | 16.15±6.23 | 20.12±4.79 |
|  |  |  | T | CD3+ | 68.15±7.9 | 69.14±5.24 |
| Zhang | 2014 | 60/60 | Th1 | CD4+/IFNγ+ | 3.98±0.78 | 6.82±1.28 |
|  |  |  | Th17 | CD4+IL17+ | 1.986±0.48 | 0.678±0.38 |
|  |  |  | Treg | CD4+CD25+ | 0.98±0.32 | 2.19±0.78 |
| Zhang | 2014 | 10/10 | Th17 | CD4+CCR4+CCR6+ | 4.14±0.46 | 2.14±0.39 |
|  |  |  | Treg | CD4+CD25+FoxP3+ | 1.13±0.17 | 2.44±0.16 |
| Zhang | 2012 | 32/20 | Th1 | CD4+/IFNγ+ | 11.05±3.41 | 10.37±2 |
|  |  |  | Th17 | CD4+INFγ-IL17+ | 2.58±0.86 | 1.07±0.26 |
| Zhang | 2008 | 78/50 | NK | CD3-CD56+ | 19.14±6.12 | 18.71±6.31 |
|  |  |  | Treg in CD4+T | CD4+CD25+CD127lo/- | 4.18±1.21 | 4.99±1.23 |
| Zhao | 2013 | 21/20 | Th17 in CD4+T | CD4+IL-17+ | 2.89±1.77 | 1.34±0.28 |
|  |  |  | Th17/Treg ratio | / | 1.24±0.45 | 0.42±0.24 |
|  |  |  | Treg in CD4+T | CD4+CD25+FoxP3+ | 3.9±1.2 | 4.9±1.2 |
| Zhao | 2011 | 14/18 | Treg | CD4+CD25hiCD127lo/- | 0.57±0.29 | 1.65±0.75 |
| Zhao | 2009 | 30/30 | CD4+T | CD4+ | 29.24±9.22 | 40.09±6.86 |
|  |  |  | CD8+T | CD8+ | 32.91±6.86 | 25.6±5.97 |
| Zhong | 2014 | 78/30 | Th1 | CD3+CD8-INFγ+ | 15.86±3.3 | 12.05±2.35 |
|  |  |  | Th17 | CD3+CD8-IL-17+ | 1.57±0.78 | 0.82±0.35 |
|  |  |  | Th2 | CD3+CD8-IL-4+ | 1.51±0.51 | 1.98±0.6 |
| Zhu | 2017 | 42/42 | CD4+T | CD4+ | 54.14±1.92 | 45.79±2.68 |
| Zhu | 2016 | 30/30 | NK | CD3-CD16+CD56+ | 11.03±5.2 | 14.58±6.57 |
| Zhu | 2000 | 14/7 | Th1 | CD4+/IFNγ+ | 10.25±6.89 | 7.94±3.38 |
|  |  |  | Th1/Th2 ratio | / | 6.54±6.3 | 4.6±3.43 |
|  |  |  | Th2 | CD4+IL-4+ | 1.98±0.73 | 2.07±0.7 |

Lymphocyte proportions: percentage of each lymphocyte subset in peripheral blood mononuclear cells (PBMC), or in T cells or CD4+ T cells if stated specifically, provided in the form of “mean±sd”.
